# Biochar for agronomy, animal farming, anaerobic digestion, composting, water treatment, soil remediation, construction, energy storage, and carbon sequestration: a review

**DOI:** 10.1007/s10311-022-01424-x

**Published:** 2022-05-07

**Authors:** Ahmed I. Osman, Samer Fawzy, Mohamed Farghali, Marwa El-Azazy, Ahmed M. Elgarahy, Ramy Amer Fahim, M. I. A. Abdel Maksoud, Abbas Abdullah Ajlan, Mahmoud Yousry, Yasmeen Saleem, David W. Rooney

**Affiliations:** 1 School of Chemistry and Chemical Engineering, Queen’s University Belfast, David Keir Building, Stranmillis Road, Belfast, BT9 5AG Northern Ireland UK; 2 Graduate School of Animal and Food Hygiene, Obihiro University of Agriculture and Veterinary Medicine, Obihiro, Hokkaido 080-8555 Japan; 3 Department of Animal and Poultry Hygiene and Environmental Sanitation, Faculty of Veterinary Medicine, Assiut University, Assiut, 71526 Egypt; 4 Department of Chemistry, Department of Chemistry and Earth Sciences, College of Arts and Sciences, Qatar University, 2713 Doha, Qatar; 5 Environmental Science Department, Faculty of Science, Port Said University, Port Said, Egypt; 6 Egyptian Propylene and Polypropylene Company (EPPC), Port-Said, Egypt; 7 National Center for Radiation Research and Technology (NCRRT), Egyptian Atomic Energy Authority (EAEA), Cairo, Egypt; 8 Department of Chemistry -Faculty of Applied Science, Taiz University, P.O.Box 6803, Taiz, Yemen; 9 Faculty of Engineering, Al-Azhar University, Cairo, 11651 Egypt; 10 Cemart for Building Materials and Insulation, postcode 11765, Cairo, Egypt; 11 Institute of Food and Agricultural Sciences, Soil and Water Science, The University of Florida, Gainesville, FL 32611 USA

**Keywords:** Climate change mitigation, Biochar applications, Carbon sink, Biochar-based fertilisers, Environmental remediation, Energy storage, Biochar in construction

## Abstract

In the context of climate change and the circular economy, biochar has recently found many applications in various sectors as a versatile and recycled material. Here, we review application of biochar-based for carbon sink, covering agronomy, animal farming, anaerobic digestion, composting, environmental remediation, construction, and energy storage. The ultimate storage reservoirs for biochar are soils, civil infrastructure, and landfills. Biochar-based fertilisers, which combine traditional fertilisers with biochar as a nutrient carrier, are promising in agronomy. The use of biochar as a feed additive for animals shows benefits in terms of animal growth, gut microbiota, reduced enteric methane production, egg yield, and endo-toxicant mitigation. Biochar enhances anaerobic digestion operations, primarily for biogas generation and upgrading, performance and sustainability, and the mitigation of inhibitory impurities. In composts, biochar controls the release of greenhouse gases and enhances microbial activity. Co-composted biochar improves soil properties and enhances crop productivity. Pristine and engineered biochar can also be employed for water and soil remediation to remove pollutants. In construction, biochar can be added to cement or asphalt, thus conferring structural and functional advantages. Incorporating biochar in biocomposites improves insulation, electromagnetic radiation protection and moisture control. Finally, synthesising biochar-based materials for energy storage applications requires additional functionalisation.

## Introduction

Carbon sequestration is the process of capturing atmospheric carbon and storing it in a stable form for extended periods. In comparison with the other carbon removal technologies addressed in the literature, biochar has demonstrated great promise in various ways. This encompasses the technological feasibility, scalability possibilities, carbon removal costs, carbon stability and permanence, verification and monitoring, as well as the benefits associated with the various possible carbon sink applications (Fawzy et al. 2020). Carbon capture and storage via biochar production is technologically viable and has the potential to be commercially feasible, particularly given the current state of the carbon sink economy (Osman et al. 2020).

Carbon sequestration via biochar production is a relatively simple concept. During plant growth, plants take up atmospheric carbon via photosynthesis, and the carbon is stored within the plant structure for as long as the plant exists. When the plant dies, however, the natural decay process returns the carbon to the atmosphere, thus completing the natural carbon cycle. The synthesis of biochar disrupts the carbon cycle by converting it to a stable form that can withstand degradation, hence avoiding emissions of greenhouse gases back to the atmosphere (Qambrani et al. 2017; Brassard et al. 2016). Combining photosynthesis and pyrolytic conversion enables the creation of an effective carbon removal system. Biochar production on a large scale should eventually affect the atmospheric carbon balance by lowering atmospheric carbon concentrations.

To produce stable biochar that is resistant to degradation and can remain stable in potential reservoirs for hundreds or thousands of years, it is necessary to select feedstocks carefully and optimise processing conditions in order to meet reservoir-specific requirements while achieving the highest attainable stability (Fawzy et al. 2021). Additionally, this must be achieved in the most sustainable way possible. While biomass waste is a priority to be utilised for reducing potential emissions and boosting the circular economy, the value and impact of fast-growing speciality crops should not be underestimated, provided they are grown sustainably. If specialised feedstocks are cultivated, the land, water, and nutrient resources used should not directly conflict with food production systems. In general, eligibility of feedstocks must be determined for certification purposes. Additionally, the thermochemical conversion process should be energy efficient, and any pyrolytic gases or waste heat generated should be re-used within the process to minimise emissions (EBC 2012a). Fossil-based energy should be excluded from the manufacturing process and used sparingly in agriculture and transportation, where necessary.

Furthermore, the final application of the biochar is critical to its sustainability as a carbon sink and should be undertaken as sustainably as possible while following regulatory and technical requirements. Biochar can be used as a carbon sink in various applications if they are not related to energy production. Furthermore, the biochar must not be subjected to thermal degradation or oxidisation during its service life or at termination (Schmidt et al. 2019a). Herein, we critically review and evaluate the academic literature on various biochar-based carbon sink applications, covering agronomy, animal farming, biological process stimulation such as anaerobic digestion and composting, environmental remediation, civil infrastructure, and finally, energy storage, where the main objective is to promote atmospheric carbon removal while facilitating enhanced utilisation opportunities and secured carbon storage. While biochar can be used in a variety of applications and cascaded value chains, the ultimate storage reservoirs for biochar are soils, civil infrastructure, and landfills. However, despite optimising biochar production to satisfy application-specific requirements, carbon stability should continue to be the most essential attribute for biochar to fulfil its sequestration purpose once finally applied to such reservoirs for extended periods.

## Agronomy

The literature has a wealth of information on the long-term storage of biochar in the terrestrial carbon pool via agricultural and forest soils. The soil is the largest terrestrial carbon sink, and the impact of biochar application on soils has gained considerable academic and commercial interest over the last two decades. It has been reported that if biochar is produced under the proper conditions to achieve carbon stability, it can be safely stored in soils for centuries.

Numerous studies have reported the persistence of carbon in soils. Wang et al. conducted a meta-analysis of 24 studies to determine the stability of biochar in soil. The researchers conducted a meta-analysis of biochar decomposition and calculated its mean residence time using 128 observations. The degradation rates varied significantly according to the feedstock type, processing conditions, duration of the experiment, and soil clay content. The results demonstrated that the labile carbon pool has a mean residence time of 108 days, and the stable carbon pool has a mean residence time of 556 years, with each pool accounting for 3 and 97% of the total carbon, respectively. This clearly shows that only a small percentage of biochar is bioavailable and that a substantial portion contributes to long-term carbon sequestration. When applied to soils, biochar is subjected to biotic, abiotic, and indirect stresses, all of which affect the rate of mineralisation (Wang et al. 2016).

Highly stable carbon should withstand such stresses, and as previously discussed, this depends on the feedstock and processing conditions. Apart from its potential for carbon sequestration, biochar has been reported to have many agronomic benefits. Biochar application, in general, is argued to have a significant effect on soil quality and fertility. Additionally, improvements in nutrient cycling have been reported, as well as an increase in water and nutrient retention. In theory, the proper application of biochar can significantly impact crop productivity, water and nutrient efficiency, and soil health. Additionally, it has been reported that biochar application can aid in the reduction of greenhouse gas emissions such as carbon dioxide, methane and nitrous oxide from soils (Fawzy et al. 2020), though the reported results are inconsistent (Semida et al. 2019; Xiao et al. 2019). The benefits outlined above are due to the biochar's effect on soil physical, chemical and biological properties such as porosity and bulk density, soil water dynamics, acidification, interaction with soil organic matter and inhibition of priming effect, and stimulation of soil microbial activity and dynamics (Oni et al. 2019; Dai et al. 2020; Tenic et al. 2020).

**Fig. 1:**
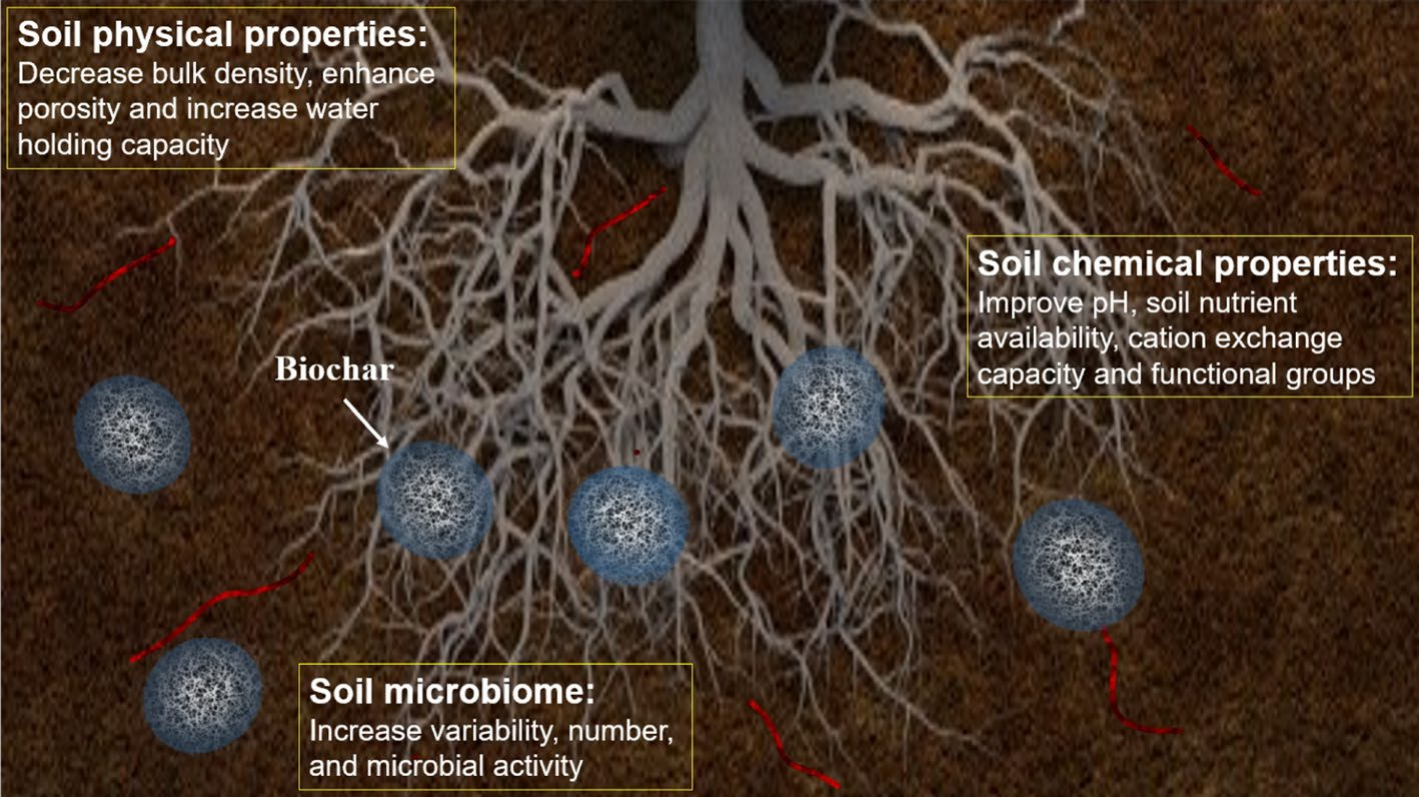
Biochar has a significant role in improving the chemical, physical, and microbiological properties of soil. Among the chemical properties of soil that can be improved are pH, nutrient availability, cation-exchange capacity and functional groups. Additionally, soil physical properties such as bulk density, porosity, and water holding capacity properties can be improved. Moreover, soil biological properties are enhanced by the addition of a significant amount of bioavailable nutrients, which improve the variety, number, and activity of soil microorganisms

Figure 1 depicts the impact of biochar application on soil physical, chemical and biological properties. While the results generally indicate positive effects, there have been instances where biochar application resulted in negative outcomes. In general, the results reported in the literature are dependent on the type of feedstock used and the production conditions, the amount of biochar applied, the type of soil used, the specific cropping system, and cultivation management techniques deployed (Fawzy et al. 2020; Fawzy et al. 2021; Maraseni 2010; Purakayastha et al. 2019).

### Biochar-based fertilisers

There is disagreement in the literature regarding the use of biochar as a fertiliser, with some arguing for biochar as a fertiliser (Kumar et al. 2021a), while others downplay its utility for this purpose (Gelardi and Parikh 2021; Ippolito et al. 2020). As previously discussed, the discrepancy could be explained by the difference in climate and soil conditions, the feedstocks used for biochar production, or by the processing conditions employed, as it is well established that the nutrient content of biochar is dependent on the raw materials used and the conditions of heat treatment (Tomczyk et al. 2020).

In general, biochar contains a small amount of key nutrients (Ippolito et al. 2020). This would require large amounts of biochar to be added to the soil, between 10 and 50 t ha^−1^, depending on the soil and biochar characteristics. This imposes an economic burden on the farmer, thereby limiting its use (Chunxue et al. 2015; Jin et al. 2019). Additionally, biochar additions greater than 50 t ha^−1^ have a detrimental effect on the soil microbial community, impairing its fertility. Moreover, it inhibits plant germination and early growth in the soil when applied at such high rates (Joseph et al. 2021). Furthermore, nutrients are released into the soil during the initial days following the addition of biochar (Zhao et al. 2016), which reduces the plant's efficiency in utilising these nutrients, thereby affecting the crop's productivity and quality.

Thus, biochar is frequently used as a soil amendment rather than a fertiliser, to develop the physical and chemical properties of the soil, while low addition rates (1 t ha^−1^) have been used as a nutrient carrier to increase the efficiency of fertiliser use and reduce nutrient losses (Joseph et al. 2021). If, however, biochar is used as a fertiliser, its nutrient content is typically supplemented with a source of nutrients such as chemical or organic fertilisers. Additionally, as discussed in the following section, biochar can be coated with a variety of materials to facilitate the slow release of these nutrients.

Chemical fertilisers are critical in modern agriculture, and their importance grows as the population continues to expand. Nonetheless, the plant's efficiency in using it is low, usually around 30–35%, resulting in economic and environmental consequences (Li et al. 2019a; Liu et al. 2019a). As a result, scientists are working to develop new ways to maximise the benefits of chemical fertilisers while minimising their adverse effects on the environment and financial costs to farms. In this context, biochar-based fertilisers, a process that combines traditional fertilisers and biochar as a carrier, have emerged as an important topic of agricultural research (Li et al. 2019a; Liu et al. 2019b). The following sections explore the potential for using biochar as a nutrient carrier, preparation techniques, and discusses the impact of using biochar-based fertilisers on agriculture and the environment.

### Biochar-based fertiliser preparation techniques

Numerous techniques for preparing biochar-based fertilisers have been discussed in the literature, including mixing, impregnation, co-pyrolysis, and encapsulation/coating. This section will explore each of these approaches.

#### Mixing

Biochar can be mixed with conventional fertilisers such as compound fertilisers containing nitrogen, phosphorus, and potassium, urea fertilisers, poultry manure, compost, and organic fertilisers prior to or during crop cultivation. Additionally, other additives, such as starch, clay, and alginate, which act as binders, can be used in predetermined proportions to create biochar-based fertilisers (Adekiya et al. 2020; Kulczycki et al. 2020; Puga et al. 2020a). After mixing, the nutrient-rich biochar can be ground to a consistent particle size and then granulated or pelletised. This is to minimise transportation costs, facilitate handling, and prevent the loss of biochar powder due to strong winds or heavy rain (Yu et al. 2021a; Shin and Park 2018).

#### Impregnation

Impregnation is the process of adding nutrients in solution form to biochar over a specified period and at a specified rate. This facilitates the sorption of such nutrients onto the surface and pores of the biochar, and the nutrient-enriched biochar is then dried (Sim et al. 2021). Common nutrients in this process are nitrogen (Liu et al. 2019b; Chen et al. 2018a)**,** phosphorous (Gwenzi et al. 2018), and potassium (Chandra et al. 2020; Khan et al. 2021a). A recent study reported that after undergoing pyrolysis using agricultural waste, the synthesised biochar was impregnated with macro and micronutrients (Lateef et al. 2019). The results indicated that the biochar nanocomposite had a neutral pH and a porous structure of 55.9 nm, indicating that it was capable of nutrient adsorption. Additionally, salt index, slow-release column, water absorbance, and retention studies indicated that the synthesised biochar nanocomposite has a high potential for use as a nano-fertiliser.

#### Co-pyrolysis

Previously, biochar was treated with a source of nutrients, either by mixing it into the biochar or by adding the nutrient solution directly to the biochar. However, in this case, nutrients are added to the biomass in the form of a solid substance (Zhao et al. 2016; Lustosa Filho et al. 2017) or a solution (Chen et al. 2020a) and the biomass is then pyrolysed in the presence of such nutrients, a process referred to as co-pyrolysis. Co-pyrolysis of biomass with phosphorous sources has been proposed as a solution to developing improved biochar-based fertilisers with a stable form of carbon and a slow nutrient release mechanism (Zhao et al. 2016; Lustosa Filho et al. 2020). Furthermore, An et al. (2020) reported co-pyrolysis of biomass (cotton straw), with nutrients such as potassium phosphate and bentonite under microwave irradiation, as a novel strategy for synthesising improved quality biochar-based slow-release fertilisers.

#### Coating and encapsulation

While biochar-based fertilisers have the potential to reduce nutrient loss and increase soil water retention, scientists are focusing their efforts on promoting sustainable nutrient release. Recently, three methods for enhancing the sustained release mechanism have been developed, with the primary goal of reducing nutrient leaching and increasing nitrogen use efficiency. The first approach, either biochar alone or in combination with another material, is used to coat conventional fertilisers such as urea (Jia et al. 2020; Mikos-Szymańska et al. 2019; Chen et al. 2018b). The second technique involves coating the biochar with another material, such as organic matter. Hagemann et al. (2017a) stated that they coated the inner (pores) and outer surfaces of biochar with nutrient-dense organic matter, which resulted in extraordinary nutrient and water retention.

The third method is referred to as integrated co-pyrolysis and coating. In this scenario, biochar is co-pyrolysed with chemical fertilisers and then coated with another material such as a polymer or biodegradable biofilm (An et al. 2021a, b). In the coating methods described, the more hydrophobic groups in the membrane and the greater its thickness, the fewer nutrients are released into the soil, resulting in less fixation, leaching, or volatilisation in the soil. Thus, the appropriate amount of hydrophobic substance and the appropriate membrane thickness must be determined to ensure that the plant receives an adequate supply of nutrients (An et al. 2021b; Azeem et al. 2016).

### Biochar engineering methods for enhanced performance

Pristine biochar has some limitations due to its physicochemical properties, such as a negatively charged surface, a small specific surface area, and a deficiency of acidic functional groups (Yang et al. 2019a), which confines its application to adsorbing anionic nutrients, such as nitrate and phosphate. Therefore, biochar is modified to overcome the barriers that prevent it from being used for a particular purpose. Currently, research on biochar modification for fertiliser production is insufficient. However, it is possible to benefit from biochar modification methods for other purposes such as pollutant removal and adapt these methods to suit the modification of biochar for biochar-based fertiliser production.

Biochar-based fertiliser production requires the development of certain characteristics of pristine biochar for it to be suitable for use as a fertiliser. For example, increasing the amount of potassium and ammonium carried by the biochar, introducing new types of nutrients such as phosphorous and nitrate anions, and making the surface of the biochar more hydrophilic to facilitate the adsorption of nutrients and the subsequent exchange with the soil aqueous solution when added to the soil. Additionally, the pores in the biochar must be larger than the size of the nutrient ions and charged appropriately in order for them to adsorb on the biochar. These desired biochar properties must be developed through modifications to the material's physical properties, such as its specific surface area and pore structure characteristics, as well as its chemical properties, such as its surface functional groups. Physical and chemical methods are generally employed to modify biochar. Tables 1 and 2 illustrate the biochar modification methods proposed for producing engineered biochar-based fertilisers, where such methods can effectively enhance biochar properties to produce an effective fertiliser. However, it should be noted that practical experiments must be conducted to verify the feasibility of producing plant-nutrient-compatible fertilisers.

**Table 1:** Physical techniques of biochar modification include steam or gas activation, ultrasonic treatment and ball milling

Modification type	Method of modification	Modification agent	Process of modification	Key findings	References
Physical	Steam/gas activation	Steam	Biochar was heated from 25 °C under nitrogen flow and preheated 500–800 °C steam	Surface area increased from181 to 322 m^2^ g^−1^	Shim et al. (2015)
		Steam	For 45 min, biochar samples were treated with 5 mL min^−1^ of steam	Reduced equilibrium time from > 24 h to ∼ 4 h Improved adsorption capacities of sulphamethazine up to 98%	Rajapaksha et al. (2016)
		Carbon dioxide	Biochar was treated to a system that ran on carbon dioxide (CO_2_) at a rate of 0.15 L min^−1^ The temperature was raised at a rate of 10 °C min^−1^ until 900 °C, then held at 900 °C for 60 min	Created a consistent pore distribution Developed total pore volume of 0.0014–0.0468 cm^3^ g^−1^ Increase surface area from 0.2 to 80.5 m^2^ g^−1^	Franciski et al. (2018)
		Carbon dioxide and ammonia	The biochar was heated gradually to 500–900 °C, using a heating rate of 10 °C min^−1^, with nitrogen (99.99%); at 400 ml min^−1^ purging After the temperature reached the desired value, nitrogen was replaced by carbon dioxide (99.999%); 100 ml min^−1^, or ammonia (99.999%); 80 ml min^−1^ or CO_2_–ammonia (CO_2_ = 100 ml min^−1^ and ammonia = 80 ml min^−1^)	Increased surface area significantly to about 627.15 m^2^ g^−1^ from 224.12 m^2^ g^−1^ Introduced nitrogen-containing groups into the biochar	Zhang et al. (2014a)
	Ultrasonic treatment	Ultrasonic sonicator	Water bamboo biochar was exposed to ultrasound (frequency of 20 kHz and electric power of 65 W) for different irradiation times Air saturated de-ionised water was employed in all tests A water bath was employed to maintain the water temperature at 25 °C	The surface area and pore volume increased from 56.3 to 141.2 m^2^ g^−1^ and 0.013 to 0.039 cm^3^ g^−1^, respectively Ability of adsorption increased upon 3.486 mg g^−1^ of reactive black 5	Nguyen et al. (2021)
		Low-frequency ultrasound	Modification of biochar was achieved under 20 kHz low power ultrasound irradiation Radiation exposure periods are 30 s, 1, and 3 min	Exfoliated the biochar’s graphitic clusters Created new micropores Opened the blocked pores Enhanced the functionalisation efficiency Boosted the adsorption capacity	Chatterjee et al. (2019a) Chatterjee et al. (2018)
		A probe-type ultrasonic vibrator	Biochar dispersion was prepared by vibrating 3 g of biochar in 80 mL water in a 100 mL glass beaker, placed in a cooling bath After sonication, the aqueous suspension was passed through a 500 μm sieve The sieved suspension was then centrifuged to keep the nanosized particles suspended in the supernatant based on Stokes Law The suspension was placed in a 50 mL glass centrifuge tube, centrifuged at 3500 g for 24 min, and then repeated to extract biochar-NPs	Increased the surface area of biochar from 0.76 m^2^ g^−1^ and its nano- form showed the largest surface area of 36.39 m^2^ g^−1^ Pore volume was significantly lower than their macro- counterparts	Oleszczuk et al. (2016)
	Ball milling	Planetary ball mill machine	Biochar was placed in a planetary ball mill machine (PQ-N2, Across International, New Jersey, USA) within agate jars (500 mL) with balls (diameter of 6 mm, 180 g in each jar) and operated at 300 rpm in ambient air and with rotation direction altered every 0.5 h	The specific surface areas of the ball milled-biochar were all greater than that of unmilled biochar	Lyu et al. (2018a)

The surface area, total pore volume, introduced nitrogen-containing functional groups, and adsorption are the most important properties that are enhanced via physical modification. As a result of these modifications, biochar's ability to function as a fertiliser is improved

**Table 2:** Chemical techniques of biochar modification include acid and alkali treatments

Modification type	Modification method	Modification agent	Process of modification	Key findings	References
Chemical modification	Acid treatment	Phosphoric acid Nitric acid	Biochar samples were modified by adding concentrated phosphoric acid or concentrated nitric acid or phosphoric acid and nitric acid Acid equivalence corresponded to 0.25 cmol Kg^−1^	Water solubility of biochar increased Nitrate and phosphate groups increased Improved plant growth in calcareous soils	Sahin et al. (2017)
		Sulphuric acid Nitric acid Hydrochloric acid	Tea-waste biochar samples were modified by the addition of 10% sulphuric acid, 69% nitric acid, 5 M hydrochloric acid to 10 g of Tea-waste biochar at (50–60 °C)	Oxygenated surface functional groups have a notable contribution to cation-exchange capacity, especially at high pH At basic pH, the functional moieties are deprotonated, and surface complexation is promoted For biochar-300, the highest cation-exchange capacity was observed when it was subjected to nitric acid modification, and for biochar-500, the highest value was observed in hydrochloric acid modification Low point of zero charge values allow for the sorption of cationic species under mildly acidic conditions through electrostatic interactions	Peiris et al. (2019)
		Hydrochloric acid Sulphuric acid Hydrogen peroxide	The biochar modification was performed with a Soxhlet extraction apparatus in which 200 cm^3^ of liquid was used. The modifiers were added into a 250 cm^3^ round bottom pyrex flask with 10 g of biochar and reacted at room temperature with a water condenser for 24 h – The modified biochar was washed with de-ionised water and dried in an oven at 105 °C	Provided a relatively high ammonium cation sorption capacity Oxygen-containing groups on the biochar surface increased The inorganic minerals were washed Polarity increased Aromaticity decreased	Wang et al. (2020a)
	Alkali treatment	Potassium hydroxide Potassium carbonate Sodium hydroxide Sodium carbonate	The biochar was impregnated with several alkali agents at a mass ratio of 3:1 (alkali chemical/biochar) The chemically activated biochar was then heated under nitrogen at different temperatures 600, 700, 800 and 900 °C for 3 h Afterwards, the biochar sample was cooled, washed with hydrochloric acid and distilled water and oven-dried at 105 °C	The introduction of oxygenated groups on biochar surface An increase in the surface basicity The temperature to beyond 700 °C was not effective in improving the nitric oxide uptake capacity The use of alkali agents improved the nitric oxide adsorption capacity of the modified biochar by 3.3–3.5 times that of the parent biochar	Anthonysamy et al. (2021)
		Potassium hydroxide	Biochar was soaked in 30% potassium hydroxide at room temperature for 8 h Furthermore, it was washed with purified water to neutrality and dried at 105 °C	Pore size was mainly mesoporous Surface area increased from 194.77 to 712.07 m^2^ g^−1^	Liu et al. (2020a)

The most important properties of biochar that are enhanced via chemical modification include oxygen-containing functional groups, cation-exchange capacity, pore size, and surface area. As a result of these modifications, biochar's ability to function as a fertiliser is enhanced

Additionally, it is worth considering the trade-off between activating biochar and achieving long-term carbon sequestration. The issue related to physical activation through thermal treatment is the excessive loss of carbon as well as high energy consumption during high-temperature activation, which compromises the carbon sequestration potential of the modified biochar. Furthermore, the chemical activation route may impose an environmental burden due to the use of chemicals. In conclusion, we recommend further research on biochar activation in order to establish the optimal conditions for biochar production and activation that balance carbon stability and applicability within a broad range of agricultural and environmental applications. Additionally, conducting life-cycle analysis to determine the impact of biochar modification on the environment and carbon sequestration potential is critical.

#### Physical modification methods

In general, the most frequently used physical methods for biochar modification are steam/gas activation, ultrasonication, and ball milling. Physical modification increases the surface area and total pore volume of the biochar, as well as facilitates the incorporation of nitrogen-containing groups and enhances the adsorption capacity. As a result of these modifications, biochar's ability to function as a fertiliser is enhanced.

The impact of steam/gas activation on biochar properties is influenced by the activation temperature, the mass ratio of steam or gas to biochar, and the duration of the activation. In general, as more carbon atoms are removed from the surface of biochar, the volume/radius of pores and surface area increase in line with the steam temperature and treatment time (Sajjadi et al. 2019a). Furthermore, despite the fact that steam is a weak oxidant, steam/gas activation is used to augment biochar surfaces with oxygen-containing functional groups (e.g. carboxylic, carbonyl, ether, and phenolic hydroxyl groups), thereby increasing the surfaces' hydrophilicity (Ahmed et al. 2016). While steam/gas activation is simple, it consumes a lot of energy due to the high temperature required. As a result, microwave activation, ultrasonication, plasma treatment, and electrochemical modification strategies have recently grown in popularity as low-cost methods for increasing biochar adsorption capacity (Bushra and Remya 2020).

Ultrasonic treatment of biochar results in beneficial chemical and physical changes, including carboxylation, hydrogenation, increased reaction rates and increased internal surface area (Sajjadi et al. 2019a). This type of treatment requires significantly less energy than conventional activation methods. Cavitation induced by ultrasound waves exfoliates and disintegrates the regular shape of the biochar's graphitic oxide layers, cleans smooth surfaces, and increases the porosity and permeability of the carbonaceous structure of the biochar (Sajjadi et al. 2019b). The literature reports that biochar had become nanoscale after being exposed to ultrasound waves, resulting in a significant increase in surface area (Oleszczuk et al. 2016). However, increasing its frequency for an extended period may result in the destruction of the biochar structure and pores. As a result, proper conditions must be adopted for this strategy to be effective (Nguyen et al. 2021).

Ball milling is a simple and common approach used for enhancing the properties of biochar. Ball milling is a highly effective method for reducing the size of biochar to a nanoscale (less than 100 nm in size), which is referred to as nanobiochar (Wang et al. 2017a; Liu et al. 2018a). Nanobiochar has gained popularity in recent years due to its ability to combine the benefits of nanotechnology and biochar technology, as well as its beneficial chemical and physical properties. The rotational speed, ball-to-power mass ratio, and milling duration all have an effect on the final nanobiochar's particle size and surface energy (Ramanayaka et al. 2020).

Nanobiochar is distinguished from pristine biochar by its substantially larger surface area, graphitic character and significantly negative zeta potential (Oleszczuk et al. 2016). Additionally, nanobiochar produced at low temperatures, such as 300 and 400 °C, followed by ball milling, has a surface area range of 5.6–47.2 m^2^ g^−1^, whereas nanobiochar produced at high temperatures, such as 450 and 600 °C, has a much larger surface area range of 342–430 m^2^ g^−1^ (Ramanayaka et al. 2020; Lyu et al. 2018a). Ball-milled biochars had finer particle sizes of 140–250 nm vs 0.5–1 mm for unmilled biochar, and a higher concentration of oxygen-containing functional groups of 2.2–4.4 mmol g^−1^ vs 0.8–2.9 mmol g^−1^ for unmilled biochar (Lyu et al. 2019). However, due to the mobility of nanobiochar, off-site migration of pesticides and other pollutants along the soil profile may pose a risk to groundwater (Wang et al. 2017a).

#### Chemical modification methods

Chemical modification is becoming more prevalent, most likely because it increases the surface area and enhances porosity of the biochar (Sahin et al. 2017). As a result, numerous chemical methods have been proposed to modify biochar, including acid and alkali treatment. Increasing oxygen-containing functional groups, enhancing cation-exchange capacity, pore size, and surface area are the most important properties of biochar that are modified chemically. As a result of these modifications, biochar's ability to function as a fertiliser is enhanced.

Acid treatment of biochar is one of the most used chemical strategies for removing impurities such as metals, increasing the number of mesopores, and increasing oxygen-containing functional groups such as hydroxyl, carboxyl, and others, thereby increasing its hydrophilicity and, ultimately, its adsorption capacity for polar adsorbates. Acids such as sulphuric, hydrochloric, nitric, and phosphoric acid, as well as weak acids such as oxalic and citric acid, are frequently used in acid treatment to modify biochar (Yang et al. 2019a; Deng et al. 2020; Wang and Wang 2019). Alkali modification or chemical reduction refers to the process of activating the surface of biochar with reducing agents such as sodium hydroxide, sodium carbonate, potassium carbonate (Anthonysamy et al. 2021), and potassium hydroxide (Anthonysamy et al. 2021; Liu et al. 2020a)**.** Furthermore, alkaline materials containing hydroxide ions or an amino group react with the functional groups on the surface of biochar, enhancing the sorption of negatively charged species (Ahmed et al. 2016), such as nitrate and phosphate ions, which are critical in the field of plant nutrition. Additionally, alkali treatment significantly alters the specific surface area and porosity of biochar (Liu et al. 2020a; Kumar et al. 2021b).

### Mineral–biochar composite fertilisers

**Fig. 2:**
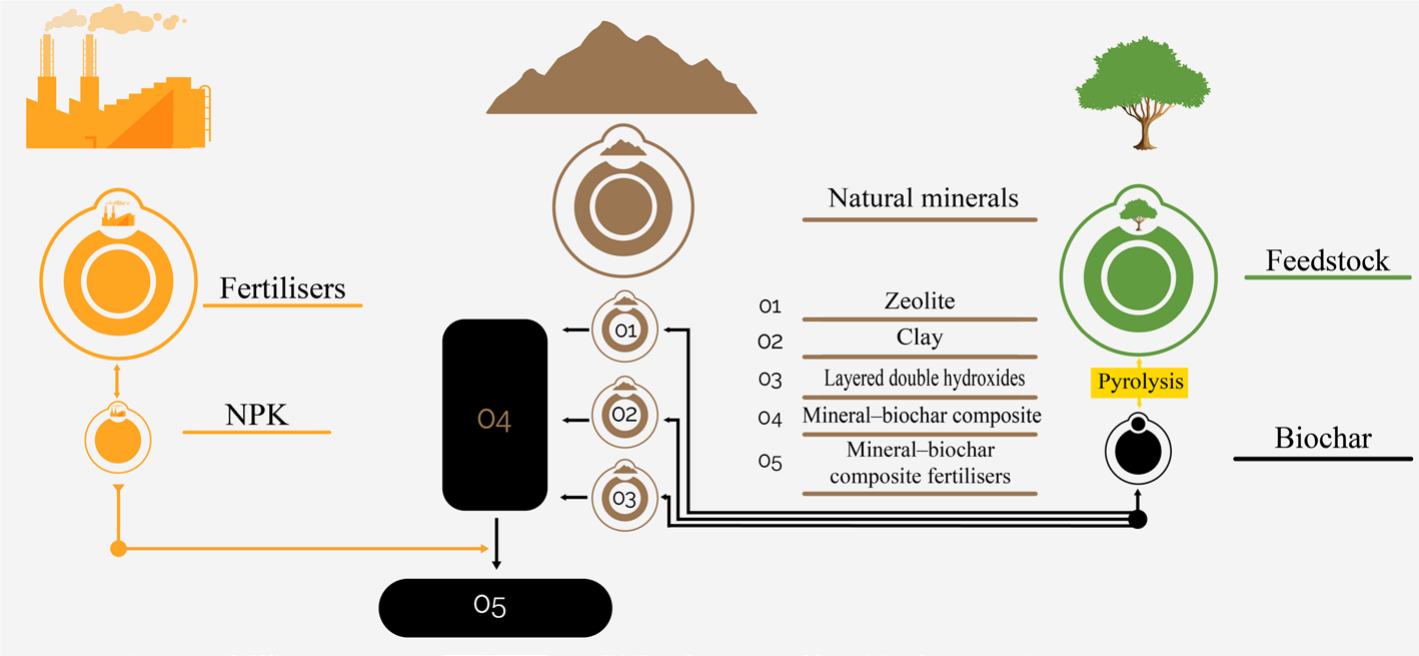
Steps of the production of a mineral-biochar composite fertilisers. Mineral-biochar composites are initially created by combining minerals like zeolite (O1), clay (O2), and layered double hydroxide (O3) with biochar. Additionally, chemical fertilisers are combined with the synthesised mineral-biochar composites (O4) to form mineral-biochar composite fertilisers (O5)

Mineral–biochar composites are produced by combining minerals such as zeolite, clay, and layered double hydroxide minerals with biochar to form composites with unique and useful properties. Then, as shown in Fig. 2, chemical fertilisers are added with the mineral–biochar composite to create mineral–biochar composite fertilisers (Wang et al. 2021a; Zhao et al. 2021a; Premarathna et al. 2019; Lesbani et al. 2021; Azimzadeh et al. 2021).

#### Zeolite-biochar composite fertilisers

Zeolites are crystalline hydrated aluminosilicates with pores comparable to those of molecules in frameworks based on extensive three-dimensional oxygen ion networks (Matsuda 2018). Zeolites exhibit extraordinary physical and chemical properties, including mechanical strength and high adsorptive ability. These attributes make them extremely useful in various applications, including agriculture and environmental protection. As a result, scientists developed zeolite–biochar composites to combine the benefits of zeolite and biochar, where biochar is more biologically and chemically stable compared to the original biomass resources used, in addition to serving as a source of long-term stored carbon in soil (Zhao et al. 2021a; Pavelic et al. 2018; Hina et al. 2015). According to Hina et al. (2015), biochar made from pine chips is a more effective ammonium sorbent than pine bark for wastewater treatment but is approximately 40% less effective than commercially available zeolite. This demonstrates the importance of using a zeolite–biochar composite as a carrier for chemical fertilisers. Another study reported that following a 90-day incubation period, the application of zeolite–biochar composites increased the soil pH from 5.60 to 8.33 and cation-exchange capacity from 6.5 to 61.28 cmol kg^−1^, indicating the possibility of using this composite as a fertiliser in combination with chemical fertilisers, in addition to stabilising pollutant metals such as cadmium, lead, and arsenic (Zheng et al. 2020).

#### Clay-biochar composite fertilisers

Clay minerals are members of the hydrous layer aluminosilicate family of minerals, which are distinguished by their layered structures composed of polymeric silica tetrahedral sheets connected to octahedral sheets (Bibi et al. 2016). Additionally, clay is distinguished by its ability to be modified to customise its properties for specific applications such as nutrient or pollutant adsorption (Abbas et al. 2017a). Furthermore, clay minerals are characterised by their small particle size and high surface-to-volume ratio. Moreover, they are readily available and inexpensive materials with exceptional chemical and mechanical stability (Wal et al. 2021). As a result, clay and biochar can be integrated to produce a composite that achieves the desirable properties of both materials.

In a study conducted by Liu et al. (2019b), a clay-biochar composite fertiliser was prepared via incorporating urea, bentonite and polyvinyl alcohols into biochar through hydrothermal synthesis. This synthesis resulted in interactions between biochar, urea, bentonite, and polyvinyl alcohols, contributing to the final products' water retention and controlled release properties. The cumulative release of nitrogen was reported to be 61.3% within 28 days when incubated in water and 54.6% within 98 days when incubated in soil, demonstrating the favourable controlled-release properties of the clay-biochar composite fertiliser. This behaviour could be explained by the fact that nutrients interact with composites in various ways. For example, ammonium can bind to biochar through Van der Waals adsorption, electrostatic attraction, the reaction of ammonium with acidic functional groups to form amides and amines, and π–π electron donor–acceptor interactions. Another study found that adding clay and iron oxide to biochar increases infiltration and the formation of clay and metal oxide nanostructures within the pores of the biochar, which promotes redox reactions that can have a significant effect on nutrient availability and uptake in plants, as well as the growth of beneficial microorganisms that improve the rhizosphere's health (Rawal et al. 2016).

According to Chen et al. (2017a), an effective composite of montmorillonite and biochar was produced. Both biochar and montmorillonite contributed to the texture and structure of the surface, resulting in a varied surface and an array of adsorptive sites. Ammonium adsorption was attributed in part to ammonium surface adsorption on montmorillonite and biochar, and in part to ammonium intercalation within the montmorillonite interlayer space. Phosphate adsorption, on the other hand, was governed by electrostatic attraction and ionic bonding on the montmorillonite-biochar composite.

#### Layered double hydroxide-biochar composite fertilisers

Environmentally friendly and biocompatible layered double hydroxide clays, also known as anionic clays, have stimulated researchers' interest due to their superior ion exchange capacity and ability to intercalate anions, as well as their high adsorption capacity and surface area characteristics (Chatterjee et al. 2019b). These characteristics are critical because certain nutrients, such as nitrates and phosphates, are available to plants as anions. Zhang et al. stated that through weak interactions, layered double hydroxide–biochar composites adsorbed nutrients such as ammonium and nitrate (i.e. van der Waals force electrostatic attraction and hydrogen bonding). As a result, nutrients are gradually released, while immobilised pollutants such as heavy metals are strongly bound via more compact mechanisms such as ionic and coordinate bonding (Zhang et al. 2018a). According to Azimzadeh et al. (2021), phosphorous-loaded layered double hydroxide-biochar significantly increased available phosphorous, corn shoot and root dry matter, and phosphorous uptake when compared to control.

### Application methods and rates

Biochar can be used in a single application and will provide benefits for many years due to its stability. Additionally, because biochar matures in soil and its interaction with soil varies over time, it is unnecessary to use biochar at each crop sowing. However, field data are currently unavailable to determine whether applying a large dose of biochar initially is more beneficial or whether yearly administrations at lower rates are preferable (Oelbermann et al. 2020).

Researchers identified a variety of techniques for applying biochar and fertilisers to the soil, including spot, ring, broadcast and incorporate (Yeboah et al. 2020). In addition to these methods, biochar can be applied directly or in combination with agricultural residues, compost, manure, and seed (Murtaza et al. 2021). Numerous studies have discovered that biochar application methods significantly affect soil characteristics. For instance, of the three distinct biochar application techniques (spot, ring, and broadcasting), the spot and ring approaches achieved the greatest improvement in observed parameters when compared to the control. The spot was the most effective method, followed by the ring, and finally the broadcasting method. As a result, when using biochar to enhance the agronomic performance of cowpea in a moderately acidic sandy soil, the application technique is critical for achieving benefits such as increased growth and yield, as well as soil fertility (Yeboah et al. 2020).

Another study reported that adding biochar and compost to the soil as a spot treatment reduced broomrape attacks on fava bean plants. Not only was a suppressive effect against broomrape achieved, but also the economic yield and protein content of fava bean were maintained, demonstrating the robust effect of spot placement of biochar and compost on fava bean sustainability in broomrape-infested areas (Saudy et al. 2021). Xia et al. (2022) stated that the optimal treatment was 100 mg kg^−1^ nitrogen fertiliser applied with biochar in deep placement modes, with a maximum nitrogen use efficiency of 46.23%. Additionally, biochar may facilitate the growth of corn in acidic soils by improving poor soil (low pH and low fertility).

### Impact on agriculture and the environment

In addition to being a source of plant nutrients, biochar-based fertilisers can be critical in amending and enhancing certain soil physical and chemical properties. This is particularly applicable in soils with unfavourable characteristics for agriculture, such as sandy soils and highly weathered soils, such as those found in the tropics, as illustrated in Table 3, as well as having an effect on microbial biomass (Li et al. 2020a).

**Table 3:** Effects of biochar application on soil properties

Properties of soil	Type of soil	Period of field experiment	Type of feedstock	Suitable application rate of biochar	Effect of biochar application	References
Bulk density	Sandy loam Alfisol	2 years	Hardwood	30 t ha^−1^	Decreased by 74.7%	Aruna et al. (2020)
Porosity	Chinese black soil	3 years	Corn straw	31.5 t ha^−1^	Increased	Jin et al. (2020)
Moisture content	Haplic Luvisol	3 years	Pinewood	50 t ha^−1^	Increased	Medyńska-Juraszek et al. (2021)
pH	Acid soil	120 days	Wood	20 t ha^−1^	Decreasing soil acidity	Shetty and Prakash (2020)
Cation-exchange capacity	RedLatosol (clayey texture) Red-Yellow Latosol (medium texture)	9 months	Coffee husk (Pyrolysed at 350 °C)	20%	Doubled the soil cation-exchange capacity (from 19.1 to 40.4 cmolc kg^−1^) Increased the soil cation-exchange capacity by ten-fold from 2.3 to 23.1 cmolc kg^−1^	Domingues et al. (2020)
Erosion resistance	Highly weathered soil	105 days	Waste wood of white lead trees	5% (wt/wt)	Reduced soil loss by 64%	Jien and Wang (2013)

This includes bulk density, porosity, moisture content, pH, cation-exchange capacity and erosion resistance. The results demonstrate the critical role of soil type, biochar type, and application rate in improving these properties

They can also benefit the environment, particularly slow-release biochar-based fertilisers, by reducing nutrient leaching and the high need for access to water sources, as well as by reducing greenhouse gas emissions, which have a detrimental effect on the ecosystem and human health. However, some studies (Knoblauch et al. 2021; Kavitha et al. 2018) have indicated that adding pristine biochar may negatively affect nutrient availability and crop productivity, which must be considered when preparing these fertilisers. These are largely determined by the properties of the biochar used (Gonzaga et al. 2018), and the characteristics and location of the soil (Puga et al. 2020b; Jeffery et al. 2017), as well as the rate at which the biochar has been added (Chrysargyris et al. 2020; Yooyen et al. 2015). As a result, applying biochar and biochar-based fertilisers to agricultural soils requires a thorough assessment of their effects on soil characteristics and crop production, which should ideally be conducted under field conditions with actual application rates.

Biochar-based fertilisers are distinguished from conventional fertilisers by their ability to gradually release nutrients into the soil, resulting in a significant reduction in nutrient loss due to leaching or volatilisation and an increase in nutrient utilisation efficiency (Puga et al. 2020b; Ndoung et al. 2021). Researchers reported a 12% increase in nitrogen utilisation efficiency when biochar-based nitrogen fertilisers were compared to urea alone in a study evaluating the effect of biochar-based nitrogen fertilisers on tropical soils. As a result, the average yield of corn increased by approximately 26%. The slow release of nitrogen from the biochar-based fertiliser was associated with an increase in nitrogen use efficiency and corn production (Puga et al. 2020b). Liao et al. demonstrated that biochar-based controlled-release nitrogen fertilisers successfully increased oilseed rape yield (16.6%) and nitrogen-use efficiency (58.79%) by gradually releasing nitrogen and modulating the abundance of functional microbes via increased soil nitrification and decreased denitrification when compared to a urea-only treatment. Biochar-based controlled-release nitrogen fertilisers increased soil nitrate, which increased rape nitrogen absorption and utilisation efficiency, thereby enhancing oilseed rape development and grain production (Liao et al. 2020).

Another study found that when biochar was enriched with phosphorus in a 50:50 (w/w) ratio using both the hot and cold methods, there was a significant increase in soil extractable phosphorus and total nitrogen, as well as high organic content, crop growth, yield, and modulation, when compared to the control and the other treatments (Wali et al. 2020). Abbas et al. (2017b) stated that biochar was applied at a rate ranging from 1 to 10% of the urea fertiliser weight in each treatment. The results indicated that using 10% biochar in combination with the recommended dose of urea increased plant height, spike length, number of tillers, number of spikelets per spike, grain yields, biomass yield, harvesting index, nitrogen concentration and uptake in grain and straw, and nitrogen agronomic efficiency by 6, 11.1, 32, 55.3, 5.4, 38, 19, 9, 19, and 26%, respectively, in comparison with the treatment using the recommended rate of nitrogen without the addition of biochar.

On the other hand, several field studies demonstrated that applying biochar to agricultural soil significantly reduced nitrate, potassium, phosphorous, magnesium, sodium, and calcium leaching (Vijay et al. 2021). Li et al. (2019a) reported that tobacco stems were pyrolysed to produce biochar. It was then added to the compound fertiliser at four concentrations of 0%, 3%, 9%, and 15% (w/w). In comparison with the control, the leaching loss of total nitrogen from the soil decreased by 8.36, 6.72, and 6.45%, respectively, and the loss of total potassium from the soil decreased by 9.18, 9.31, and 11.82% in the 3, 9, and 15% biochar with fertiliser treatments, respectively. However, because phosphorus has a low mobility in the soil profile, biochar-based fertilisers had little effect on phosphorus leaching. Additionally, biochar-based fertilisers increased ammonium, available phosphorous, and available potassium immobilisation in the soil profile.

Nitrous oxide has been estimated to have a global warming potential of 298 and 11.9 times that of carbon dioxide and methane, respectively (Domeignoz‐Horta et al. 2018). Additionally, nitrous oxide is a persistent greenhouse gas that can remain in the atmosphere for 100 years, with atmospheric nitrous oxide concentration increasing by 0.2–0.3% annually (Grutzmacher et al. 2018). Nitrous oxide is produced during the incomplete conversion (nitrification) of ammonium to nitrate by ammonium-based nitrogen fertilisers (Dawar et al. 2021).

Recently, biochar has been proposed as a means of mitigating climate change by reducing nitrous oxide emissions. In this regard, Grutzmacher et al. (2018) stated that when ammonium nitrate was used in combination with biochar, nitrous oxide emissions were reduced sevenfold in sewage sludge biochar treatment. Additionally, the fertiliser emission factor decreased with biochar amendments, ranging from 0.01 to 0.08% of the nitrogen emitted as nitrous oxide, demonstrating biochar's potential to reduce fertiliser-induced nitrous oxide emissions, with sewage sludge biochar mitigating 87% of soil-fertiliser emissions. According to another study, nitrous oxide emissions were significantly reduced by 31.4–39.9% when a fertiliser was applied in combination with biochar, compared to chemical fertiliser application alone at 200 kg nitrogen ha^−1^, and the nitrous oxide emission factor of the applied nitrogen was reduced from 1.36% when only chemical fertiliser was applied to 0.71–0.85% when fertiliser plus biochar application was used. These findings suggest that applying nitrogen fertiliser and biochar at an appropriate rate are viable strategies for reducing field-scale nitrous oxide emissions (Niu et al. 2017).

### Summary

This section demonstrated the potential utilisation of biochar within agronomic applications. Agriculture is the most common biochar-based carbon sink application discussed in the literature, where a variety of benefits can be extracted in addition to serving as a long-term carbon reservoir. However, the impact on soil and crops is inconsistent and generally depends on the type of feedstock used and processing conditions employed for biochar production, the specific cropping system, and management practices. While biochar is not a source of nutrients in and of itself, its use as a nutrient carrier has garnered substantial attention in the scientific literature.

Overall, our analysis shows that biochar-based fertilisers, a process that combines traditional fertilisers with biochar as a nutrient carrier, are a very promising and value-adding route for biochar utilisation within agronomy. Several preparation strategies were explored, where various modification treatments were proposed for enhancing the biochar’s performance. It is critical to understand the impact of such treatments on the carbon stability of the biochar as well as the overall environmental footprint, and thus, detailed life cycle assessments need to be carried out to adequately determine the biochar’s carbon removal potential. In general, the utilisation of biochar as a nutrient carrier is a highly recognised strategy, which can facilitate many agronomic benefits while allowing for long-term storage in soils. However, proficiency in biochar preparation is essential for synthesising a product that can be successfully used.

## Animal farming

Biochar value can be maximised if applied within animal farming prior to being used in various cascaded applications and final long-term storage in soils. This section will explore the various areas where biochar can be utilised in the animal farming industry and will critically assess the merits and challenges highlighted in the literature. Furthermore, the technical requirements specific to this application will be presented.

### Utilisation of biochar as a feed additive

Recently, several scholars have investigated the effects of co-feeding biochar with animal diets on cattle, poultry, pigs, and fish (Abakari et al. 2020; Al-Azzawi et al. 2021; Schubert et al. 2021; Goiri et al. 2021; Kalus et al. 2020a). Co-feeding with biochar increased milk production by 3.43% and protein–fat content by 2.63–6.32%, respectively, and reduced enteric methanogens in Australian dairy cattle by 30% (Al-Azzawi et al. 2021). Additionally, co-feeding with 2.5% biochar increased daily feed intake of laying hens, improved laying performance by 6%, and enhanced shell solidity and thickness by 10% and 6%, respectively (Kalus et al. 2020a).

Similarly, Goiri et al. discovered that co-feeding broilers with biochar at a 30 g kg^−1^ diet concentration increased daily weight gain, average body weight, and reduced feed conversion ratios compared to non-co-fed groups (Goiri et al. 2021). Furthermore, it has been shown to increase the survival and growth of aquatic fish (Mabe et al. 2018). Moreover, co-feeding with biochar reduced *Gallibacterium anatis* and *Campylobacter hepaticus* pathogens in poultry as a result of biochar's inherent adsorption properties (Willson et al. 2019). Consequently, biochar may be a viable alternative to antibiotics in the animal husbandry sector (Man et al. 2021). The unique properties of biochar may help maintain gut microbiota (methanogens) within its porous structure, thereby reducing greenhouse gas emissions from ruminants (Al-Azzawi et al. 2021; Eger et al. 2018; Mirheidari et al. 2020), which are the primary source of agricultural greenhouse gaseous emissions, and thus positively influencing global climate change. Additionally, farm production may be increased as a result of decreased chemical fertiliser use, which arises from improved animal excreta that maximise soil fertilisation quality (Joseph et al. 2015; Kalus et al. 2019).

**Fig. 3:**
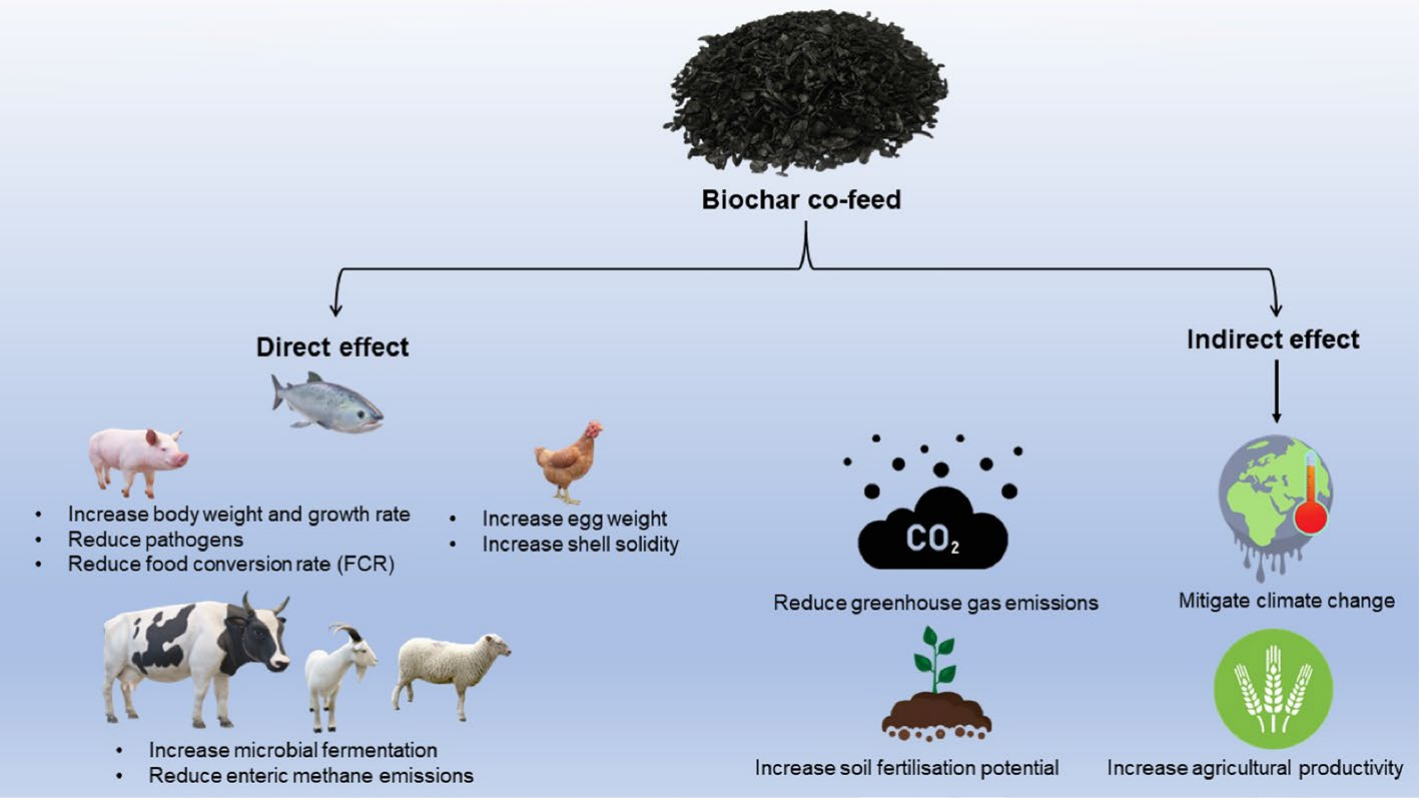
Advantages of co-feeding animals with biochar. The direct effects of adding biochar to animal feed include increased body weight and growth rate, improved microbial fermentation, pathogen reduction, and decreased enteric methane emissions. Additionally, an indirect positive impact can be generated by improving the quality of fertilisation and reducing greenhouse gas emissions, which would help to mitigate climate change

In conclusion, research indicates that co-feeding with biochar can improve animals' production, growth, immunity, and blood profile; reduce pathogens and enteric methane generation by accelerating microbial fermentation, and improve overall agricultural productivity; however, research in this field is scarce. As such, this section focusses on the effects of various biochar substrates used as supplements (biochar co-feed) on farm animals, fish, and poultry, while also highlighting scientific gaps and areas for future research. The overall effects of co-feeding biochar are depicted in Fig. 3.

#### Regulations for adopting biochar as a feed supplement

The feedstock used to produce biochar for co-feed supplements undergoes pyrolysis at temperatures ranging from 350 to 1100 °C (Man et al. 2021; Das et al. 2021). This thermal treatment, combined with the specific properties of the underlying biomass feedstock, results in biochar with a unique physicochemical structure, such as an exceptionally large surface area capable of capturing and holding minute particles, that serves as a habitat for microorganisms, as well as adsorbs undesirable compounds. Additionally, the produced biochar contains a variety of surface functional sites and mineral components that have a variety of agricultural and environmental applications (Das et al. 2020a, b). As previously discussed, the overall effect of the produced biochar is entirely dependent on the biomass type, temperature, and residence time. However, biochar used for feed applications must comply with certain requirements issued by official authorities. For instance, the European food safety authority regulates certain specifications for feed-grade biochar pursuant to specific feed regulations [Commission regulation (EC) No 178/2002 and 834/2007] (EBC 2012b).

The European biochar certification foundation, commonly referred to as EBC, is a well-known non-governmental organisation that establishes standards for sustainable biochar production in Europe. In general, various classes of biochar are promoted, based on their intended use, the unique characteristics of the produced biochar, and its toxicant content. It is critical to note that certified biochar that is permitted for use as a feed additive must be made from untreated and natural biomass (EBC, 2012b). Table 4 summarises the specific characteristics required for certification of feed-grade biochar following European biochar certification (EBC, 2012b).

**Table 4:** Biochar standards for obtaining the European certificate of feed-grade biochar

Criteria	Biochar co-feed criteria values
Polycyclic aromatic hydrocarbons	Less than 4 mg kg^−1^
Carbon content	More than 80%
Heavy metals	Arsenic: Less than 2 mg kg^−1^
	Lead: Less than 10 mg kg^−1^
	Cadmium: Less than 1 mg kg^−1^
	Mercury: Less than 0.1 mg kg^−1^
Benzo-a-pyrene	Less than 25 µg kg^−1^
The dioxin-like polychlorinated biphenyls	A start value of 0.35 ng TE kg^−1^
Polychlorinated dibenzo-p-dioxins and dibenzofurans along with dioxin-like polychlorinated biphenyls	Less than 1.25 ng TE kg^−1^
Polychlorinated dibenzo-para-dioxin/ Polychlorinated dibenzofurans	A limit of 0.75 ng TE kg^−1^
Fluor	Less than 150 mg kg^−1^
Dry matter, insoluble-, and crude- ashes	Same as standard values imposed by EU regulations for feed
Crude protein, crude fat, and crude fibre	0 g kg^−1^ due to pyrolysis effect
Paint and solvents	Free
Contaminants such as plastics, rubber, and electronic scrap	Free

All parameter values are calculated at 88% (dry matter basis). The various criteria and their values are summarised

#### Effects of co-feeding biochar on the performance of animals

Food additives, including amino acids, organic minerals, fatty acids, vitamins, and antibiotics, are frequently used in animal farming to enhance the growth operation, productivity, immune status, and protein intake of the animals. Around 90% of biochar produced in Europe is used in agro-environmental activities such as animal husbandry (cattle and poultry farming), crop production, and environmental remediation (Gerlach and Schmidt 2012). Biochar is primarily used as a feed supplement in the animal husbandry industry (Kammann et al. 2017). Biochar's annual use in agriculture is expected to increase by 12.5% over the next 5 years (Man et al. 2021). Feed-grade biochar is primarily added to feed at a rate of 0.1–4.0% of the daily feed intake (Man et al. 2021; O’Toole et al. 2016). As shown in Table 5, adding biochar to feed has the potential to increase feed intake and weight gain (Mirheidari et al. 2020; Sivilai et al. 2018); improve animal health (Bolan et al. 2021; Yıldızlı et al. 2021); facilitate toxin and contaminant detoxification, increase nutrient intake, decrease antibiotic residues, and decrease enteric methane release (Man et al. 2021; Schmidt et al. 2019b; Toth et al. 2016).

**Table 5:** Utilisation of biochar as a co-feed in animal production, including a summary of biochar's potential for improving the performance of cattle, sheep, goats, pigs, poultry, and fish, as well as the conditions under which biochar is produced and the raw substrates used

Animals	Raw substrates		Generation condition	Amount of biochar used	Main influence	References
Cattle	Rice husks		Gasification > 400 °C	0.6% of daily feed dry matter	Increased body weight Reduced methane release	Leng et al. (2012a)
				1% of daily feed dry matter with 4% rice distiller’s by-product	Increased weight gain by 15% Reduced feed conversion ratio	Phongphanith and Preston (2018)
				2–8%	Increased weight gain Reduced feed conversion ratio	Saroeun et al.(2018)
	Pine trees			0.8 and 3% of dry feed matter	0.8% improved organic matter and fibre digestibility and reduced methane generation	Winders et al. (2019)
Goats	Rice husks		Gasification > 400 °C	1% of daily feed dry matter	No influence on digestibility, feed intake, and nitrogen content	Phongpanith et al. (2013)
	Walnut shell and chicken manure		Pyrolysis at 550 °C for 3 h	0.5, 1, and 1.5% of diet dry matter	1% walnut shell and 1.5% chicken manure biochar increased digestibility, milk yield, and methane emissions	Mirheidari et al. (2019)
	Fibrous biomass		Gasification > 400 °C	1.1% of daily feed dry matter	Increased body gain Reduced feed conversion ratio Increased dry matter digestibility Increased nitrogen retention	Silivong and Preston (2015)
Sheep	Lodgepole pine and quaking aspen		Pyrolysis > 600 °C	2% of dry feed matter	Improved dry matter digestibility and intake and volatile acetate production	McAvoy et al. (2020)
	Pistachio by-product, walnut shell, and chicken manure		Pyrolysis temperatures (above 550 °C) for 3 h	1–1.5%	Improved feed conversion ratio and average daily gain No influence on dry matter intake, volatile fatty acid productions, rumen pH, and rumen protozoa	Mirheidari et al. (2020)
Pigs	Bamboo		Pyrolysis > 600 °C	0.3% of dry feed matter	Improved weight gain to 17.5% Improved the quality of marketable meat	Chu et al. (2013a)
	Spruce larch, beech, and oak-based biochar and oak		Not mentioned	2% spruce larch, beech, and oak-based biochar and 2% oak	Improved dry, organic matter, and fibre digestibility	Schubert et al. (2021)
Poultry						
Chicken	Woody green waste		Pyrolysis at 550 °C	1, 2 and 4% in daily diet	Increased egg weight by 1, 5 and 4%, respectively – Improved feed conversion ratio by 9, 14 and 12%, respectively	Prasai et al. (2018)
	Eucalyptus hardwood				Lowered poultry pathogens (*Campylobacter hepaticus* and *Gallibacterium anatis)*	Willson et al. (2019)
	Rice husks		Gasification > 400 °C	1% in daily diet	– Reduced coliforms and *E. coli* in litter and faeces – Reduced plasma triglycerides	Hien et al. (2018)
	Woody waste		Pyrolysis at 550 °C	4% in daily diet	– Decreased *Campylobacter jejuni* – Increased egg weight by 3% – Improved feed conversion ratio by 8%	Prasai et al. (2016)
	Broiler litter		Gasification	2–4%	No impact on weight gain and feed conversion ratio	Evans et al. (2017)
	Beechwood		Pyrolysis at 550 °C	2 and 4%	– Increased feed conversion ratio by 8% – Increased average body weight by 7%	Kalus et al. (2020b)
	Poultry litter		Gasification > 400 °C	5 g kg^−1^	– Increased serum albumin – Decreased serum uric acid, and restored body weight	Rashidi et al. (2020)
	Pine shaving		Not mentioned	10–20% per litter	No influence on performance, health, or litter nutrient content – Improved litter quality – Improved water absorption	Linhoss et al. (2019)
Laying hen	Beechwood biochar		Pyrolysis at 550 °C	1 and 2%	– Increased daily feed intake 6% increase in laying performance – Increased average egg mass – Increased in shell resistance to crushing and shell thickness – 1.5 and 3% biochar–aluminosilicates–glycerine mixtures reduced and increased daily feed intake, respectively	Kalus et al. (2020a)
	Biochar–aluminosilicates–glycerin mixture			1.5 and 3%		
Turkey	*Miscanthus* grass		Anaerobic conditions at 400 °C	0, 5, 10, or 20% of bedding	– Increased body weight – Lowered feed intake – Increased body weight gain – Improved litter quality	Flores et al. (2021)
	Poultry litter		Gasification > 400 °C	6.2 or 6.9% of the diet	– Improved pellet quality – Decreased live weight gain – Increased bone mineralisation	Evans et al. (2017)
Fish						
Japanese flounder *(Paralichthys olivaceus*)		Bamboo		0.25, 0.5, 1, 2, and 4% in daily diet	– Increased weight gain – Increased growth rate – Reduced feed conversion ratio – Increased protein efficiency ratio	Thu et al. (2010)
African catfish (*Clarias gariepinus)*	Palm kernel shell		Microwave pyrolysis	0 g, 150 g, 300 g, 450 g per tank	– Decreased ammonia (67%) – Decreased total suspended solids (68%) – Increased nitrogen uptake – Higher growth in lettuce	Su et al. (2020)
Tilapia (*Oreochromis mossambicus)*	Water hyacinth		Pyrolysis conditions at 300 °C for 30 min	0.5 and 1%	Increased fish weight and length (optimum at fishes fed with 1% biochar mixed diet)	Najmudeen et al. (2019)

Several scholars have examined the effect of co-feeding with biochar. For example, Castillo-González et al. concluded that adding biochar to ruminal fluid under *in vitro* conditions improved substrate digestibility (Castillo-González et al. 2014). Similarly, pine biochar was found to improve *in vitro* nutrient utilisation, protein synthesis, and ruminal fermentation while decreasing methane production (Saleem et al. 2018). Co-feeding with 0.6% biochar made from rice husk enhanced the weight of cattle by 25% when compared to those receiving no diet supplementation (Leng et al. 2012a). Phongphanith and Preston (2018) reported that adding 1% rice husk biochar with probiotic yeast additives to cattle feed improved the diet conversion ratio significantly from 11.5 to 7.9 and increased animal weight by 60%.

Additionally, supplementing 2–8% biochar with urea and molasses improved the feed conversion rate of cattle from 16.4 to 10.7% and increased weight by 43% (Saroeun et al. 2018). Winders et al. (2019) demonstrated that supplementing cattle diets with 0.8% biochar (on a dry matter basis) increased organic matter and fibre digestibility during the growing stage, while quadrupling dry matter intake during the finishing stage, as compared to non-supplemented diets. Furthermore, the authors discovered a decrease in enteric methane generation of 10.7 and 9.6% g d^−1^ during the growing and finishing stages, respectively. However, adding biochar to an artificial rumen system did not affect total gas and methane production, nutrient disappearance, protein synthesis, rumen microbiota composition, or rumen fermentation (Tamayao et al. 2021).

Similarly, adding pine biochar to the diet of heifers did not affect dry matter intake, nitrogen balance, tract digestibility, or methane production, but reduced protozoa counts and ammonia nitrogen by 0.5 and 1.0%, respectively, when compared to the control diet (Terry et al. 2019). Teoh et al. investigated the effect of daily additions of 400 and 800 mg of hardwood biochar to an *in vitro* artificial rumen. The addition of biochar had no effect on digestibility, total gas production, propionate, acetate, butyrate, rumen pH, or the rumen bacteria, archaea, or fungal biota. Using 800 mg biochar, on the other hand, reduced methane emissions (Teoh et al. 2019). In conclusion, biochar feed additives have the potential to adsorb gases, contribute to the modification of redox reactions, and act as a habitat for the colonisation of biofilms and the proliferation of microbiota. As a result, biochar co-feeding has the potential to alter rumen fermentation characteristics and reduce enteric methane generation.

Mirheidari et al. investigated the effect of supplementing the diet dry matter with 0.5, 1, and 1.5% walnut shell biochar and chicken manure biochar on rumen fermentation traits in dairy ewes. In comparison with the control, adding 1% walnut shell biochar and 1.5% chicken manure biochar to the *in vitro* study reduced methane emissions, total volatile fatty acid generation, and ammonia N, while raising pH. In *in vivo* research, supplementing dairy ewes with biochar at higher concentrations enhanced milk production, milk protein, solids non-fat portion, blood glucose, dry matter, and fibre digestibility rates, and decreased gas emissions (Mirheidari et al. 2019). Silivong and Preston determined that supplementing goats diets with 1.1% biochar increased their weight by 8.9%, improved their feed conversion ratio from 15.5 to 11.4, and improved their digestibility (Silivong and Preston 2015). McAvoy et al. (2020) investigated the effect of co-feeding quaking aspen and lodgepole pine biochar on the digestibility of the diet and overall sheep performance. They discovered that supplementing lambs' diets with biochar boosted digestibility and intake, as well as acetate generation. However, these positive results did not imply considerable improvements in body weight gain or feed conversion efficiency. Similarly, supplementing fattening lambs' diets with 1% pistachio by-product, 1% walnut shell, and 1.5% chicken manure biochar enhanced feed conversion ratio and average daily gain compared to the control. The additives, on the other hand, had no effect on dry matter intake, volatile fatty acid synthesis, rumen pH, or rumen protozoa (Mirheidari et al. 2020).

Schubert et al. investigated diets supplemented with 2% spruce, larch, beech, and oak-based biochar and 2% oak biochar on the performance of growing pigs. The authors discovered that adding biochar to the diet improved dry matter, organic matter, and fibre digestibility, with a maximal increase of 19.8 and 23.8% in crude fibre digestibility, respectively, as compared to the control (Schubert et al. 2021). Sivilai et al. (2018) demonstrated that co-feeding with 1% rice husk biochar enhanced the weight of pigs by 20.1% and the feed conversion ratio by 10.6%. Similarly, it was reported that co-feeding bamboo biochar enhanced unsaturated fatty acids and decreased saturated fatty acids, as well as improved the quality of swine carcasses (Chu et al. 2013b). Co-feeding pigs' diets with 0.3% biochar increased faecal microflora, particularly lactic acid and anaerobic bacteria, while decreasing *Salmonella spp.* and pathogenic coliform bacteria (Chu et al. 2013c). Chu et al. (2013a) demonstrated that adding 0.3 and 0.6% of bamboo biochar to pig feed decreased triglyceride, blood urea nitrogen, and lactate dehydrogenase levels in the blood while increasing immunoglobulin G, total serum antibody concentration, and *Lactobacillus spp.* Levels, as compared to the control.

Reducing feed costs, increasing poultry growth, and minimising the adverse environmental impact are recent challenges affecting the poultry industry's sustainability. Kalus et al. (2020b) investigated the effects of co-feeding broiler chickens with biochar on their weight gain and feed conversion ratio. Beechwood biochar (2–4%) and a mixture of biochar, glycerin, and aluminosilicates (3–6%) were added to the broiler's diet. Biochar co-feed reduced ammonia emissions by 17%, raised feed conversion ratio by 8%, and only the lowest doses of biochar increased body weight, whereas larger concentrations had a marginal effect. In the same context, the researchers examined the effects of the same biochar/biochar mixtures on the performance of laying hens. Biochar addition enhanced daily feed intake by 6%, and increased shell resistance to crushing and shell thickness by 10 and 6%, respectively (Kalus et al. 2020a). By adding pine shavings biochar to the turkey's diet by 0, 5, 10, or 20%, body weight increased from 16.72 to 17.0 kg, feed intake decreased from 48.1 to 45.6 kg, and feed conversion ratio improved to 2.20 from 2.31 in the control (Flores et al. 2021). By incorporating chicken litter biochar at a rate of 6.2 or 6.9% into poultry diets, pellet quality was enhanced, feed conversion ratio was decreased, and bone mineralisation was raised due to increased phosphorus and calcium bioavailability (Evans et al. 2015).

Evans et al. reported that supplementing chick diets with 2% broiler litter biochar had no adverse effect on their performance and exhibited superior granulation characteristics and moisture retention capacity (almost 90%) as compared to those formulated with zeolite or bentonite (Evans et al. 2017). Co-feeding with wood biochar increased egg weights and feed conversion ratios in egg-laying poultry as compared to those fed without biochar (Prasai et al. 2017). Biochar's high surface area and porosity make it ideal for pathogen and toxicant control. Prasai et al. (2016) demonstrated that supplementing layer meals with 4% biochar made from wood reduced pathogenic *Campylobacter jejuni* in the birds' intestine and increased egg weight and feed conversion ratio by 3.0 and 11.7%, respectively. Additionally, co-feeding with biochar can help eliminate toxins from the bird's gut and improve the intestinal flora and its vitality (Gerlach and Schmidt 2012). By feeding chicken with 1% rice husk biochar, plasma triglycerides were diminished and omega-3 fatty acids were increased (Hien et al. 2018).

Co-feeding with biochar in aquaculture has received relatively less attention. It was observed that feeding 0.5% bamboo biochar to *Paralichthys olivaceus* (juvenile Japanese flounder) enhanced growth rate, weight gain, and feed and protein conversion ratios (Thu et al. 2010). Several authors reported increased body weight, growth rate, and feed conversion ratios following co-feeding with biochar at concentrations ranging from 0.004 to 4% (Moe et al. 2009; Lan et al. 2016; Quaiyum et al. 2014). Najmudeen et al. (2019) examined the effects of co-feeding with 0.5 and 1% water hyacinth biochar on *Oreochromis mossambicus* fish aquaculture and discovered an increase in fish weight and length, with the greatest increase occurring at 1% biochar content. Abakari et al. (2020) investigated the influence of biochar on the tilapia bio-floc technology system. The researchers discovered no adverse effect on fish growth or performance and observed an improvement in water quality parameters such as total suspended solids when biochar treated fish were used compared to the control.

#### Biochar functions as a co-feeding additive

##### Mitigation of enteric methane

Methane, carbon dioxide, fluorinated gases, and nitrous oxides are the primary contributors to greenhouse gas emissions. Around 4 billion ruminants are raised worldwide, including 1.7 billion cattle and buffalos and 2.2 billion sheep and goats (Searchinger et al. 2021). Ruminants have a rumen, which is capable of using cellulose by microbial communities. Through the action of anaerobic bacteria, "enteric methane" is released from the rumen. Enteric methane emissions from domestic animals were estimated to reach 100 million tonnes of carbon dioxide-equivalent in 2018, accounting for more than a quarter of agriculture-related emissions, and is projected to increase by 50% by 2050 (Searchinger et al. 2021). Cattle and buffalos account for over 85% of global emissions, whereas sheep and goats account for 12% (Searchinger et al. 2021; FAO, 2019). Methane is primarily released via eructation, with trace amounts absorbed into the bloodstream and exhaled via the lungs (Danielsson et al. 2017).

There are two types of methane-producing/utilising microbes in the rumen: methanogenic and methanotrophic archaea, which are responsible for enteric methane production. The addition of biochar promotes the growth of methanotrophs, which offers a habitat for methane oxidation within the gut, hence lowering enteric methane emissions in ruminants (Leng et al. 2012b). Another significant factor in lowering enteric methane production is the biochar's ability to adsorb and absorb gases (Danielsson et al. 2017; Pereira et al. 2014). Thus, regular co-feeding of biochar to animals can be an effective strategy for mitigating enteric methane emissions (Leng 2018).

Incubation of biochar with ruminal fluid resulted in a 15% reduction in methane emissions (Leng et al. 2012b). Similarly, enteric methane generation was reduced by 11–17% when 9% biochar (w/w) was added (Hansen et al. 2012). *In vivo* experiments demonstrated that adding 1% biochar (w/w) to cattle diets reduced methane release by 11–13% (Leng et al. 2012a). Another study reported that adding biochar and combining it with nitrate reduced methane release by 22 and 41%, respectively (Leng et al. 2012b). Additionally, co-feeding cattle with 3.8% biochar (w/w) resulted in a reduction of 12.6 L of methane per animal per day (Khoa et al. 2018). Winders et al. (2019) revealed that adding 0.8% biochar to cattle's diet reduced enteric methane generation by 9.5% and 18.4% g kg^−1^ dry matter intake, respectively, throughout the growth and finishing stages. Saleem et al. (2018) concluded that adding 0.5% biochar to an *in vitro* rumen experiment resulted in a 25% reduction in methane production (mg d^−1^). In conclusion, biochar co-feeding has been shown to effectively reduce enteric methane release from rumens in both *in vivo* and *in vitro* experiments.

##### Elimination of contaminants in livestock

Biochar can remove various pollutants from the environment, including heavy metals, antibiotics, organic chemicals, and microplastics (Gopinath et al. 2021; Wang et al. 2019a). Animals are mostly contaminated by pollutants in their feed and water, such as insects, environmental contaminants, and diverse microbial activity. Rashidi et al. (2020) showed that diets supplemented with 5 g kg^−1^ biochar made from poultry litter adsorb and restore the body weight of broiler chickens suffering from aflatoxicosis, as well as increase the bird's performance. Co-feeding of layers with 2% biochar (w/w) significantly decreased the incidence of *Gallibacterium anatis* and *Campylobacter hepaticus* compared to non-supplemented control diets (Willson et al. 2019)*.* Similar reductions in *Campylobacter jejuni* were reported in pullet guts co-fed with biochar (Prasai et al. 2016). Biochar's adsorption capacity for contaminants is primarily determined by its specific surface area, functional groups, and sorption characteristics (Oh and Seo 2016).

### Utilisation of biochar as a litter amendment

Bedding is essential for animal health and performance, particularly in the poultry industry, but it can also have a significant environmental impact after its intended use, such as an alternative energy source or a soil amendment agent. Biochar can be added to the animal's diet and the impact on litter evaluated, or it can be directly applied to the litter or animal bedding.

Linhoss et al. investigated the effect of pine shavings biochar (0.97 kg m^2^) on litter quality and broiler performance. The authors reported that adding biochar to the litter had no negative impact on the health and performance of the birds; however, biochar at 10–20% increased the water holding capacity of the litter by 21.6 and 32.2%, respectively, when compared to litter without biochar, which was attributed to the biochar's water retention capability (Linhoss et al. 2019). A similar study indicated that treating turkey litter with 20% biochar improved the bird's performance and health (Flores et al. 2021). The essential aspect that impacts overall adsorption capacity is the biochar's specific properties, particularly large surface area (Linhoss et al. 2019; Liu et al. 2017a). Biochar addition to the litter, on the other hand, may have little effect on the pen's environmental conditions, such as ammonia (Flores et al. 2021; Ritz et al. 2011). Increasing the amount of biochar in the litter, however, may increase nitrogen adsorption (Flores et al. 2021).

Agyarko-Mintah et al. studied the effect of co-composting poultry litter and straw with biochar made from poultry litter and biochar made from green waste. The *in situ* greenhouse gas analysis demonstrated a decrease in nitrous oxide emissions of 5.0 and 4.2 g N_2_O–N kg^−1^ of total nitrogen, respectively, for the poultry litter-biochar and green-waste-biochar-amended beddings, compared with 14.0 g N_2_O–N kg^−1^ of total nitrogen for the non-amended bedding. Similarly, the methane emissions were reduced to 18- and 12-mg CH_4_–C kg^−1^ of total carbon, respectively, compared with 80 CH_4_–C kg^−1^ of total carbon for the control. Total greenhouse gas emissions over the entire process were 63, 50, and 183 kg carbon dioxide-eq t^−1^ (dry weight basis) for poultry litter-biochar-amended bedding, green waste-biochar-amended bedding, and the control, respectively. The enhanced removal capacity of biochar-amended bedding was attributed to the interaction/adsorption of nitrogen and organic materials with abundant functional groups on the biochar’s surface (Agyarko-Mintah et al. 2017).

### Utilisation of biochar for aquatic wastewater treatment

Aquaculture is typically associated with contaminated wastewater discharge, making it one of the most polluting industries. Nutrients (nitrate, ammonia, phosphate, and organic compounds), antibiotic residues and resistance genes, and pathogens are typically found in aquaculture water (Abdel-Tawwab et al. 2019; Mahari et al. 2022). Pollution, eutrophication, algal blooms, and severe changes in the ecosystem are common outcomes of discharged compounds (Granada et al. 2016). In an aquaponic tank containing African catfish, Su et al. investigated the ability of biochar as a biofilm niche for nitrifying bacteria to remove water contaminants. The researchers reported a 67 and 68% in ammonia and total suspended particles, respectively. Biochar addition also increased lettuce growth by 0.0562% per day by raising the pH of the system to 6.8, assisting in the conversion of ammonia to nitrate, and improving nitrogen utilisation by the lettuce. Furthermore, the biochar ensured that the catfish survival rate remained optimal at 100%. As a result, biochar application in aquaponics could be a viable approach for toxicant mitigation (Su et al. 2020).

In a similar study, Khiari et al. (2020) used biochar made from bamboo to reduce turbidity and suspended particles in tilapia grown in an aquaponics system. To absorb chromium, Mahmoud et al. combined biochar made from shrimp shells with graphene oxide gel (VI). At lower pH of 1, the greatest sorption capacity of 350.42 mg g^−1^ was achieved. The adsorption of chromium by biochar was driven by the biochar’s specific characteristics, such as electrostatic adsorption, porosity, and abundant functional units, which resulted in removal rates of 98, 99.2, and 99.8% from wastewater, saltwater, and tap water, respectively (Mahmoud et al. 2021). Similarly, Chen et al. (2020b) employed crab shell biochar in combination with iron oxide nanoparticles to remove lead(II) and arsenic(III) from wastewater media and found that the contaminants adsorb at rates of 62.4 mg g^−1^ and 15.8 mg g^−1^, respectively. Removal of antibiotics, such as tetracycline, chloramphenicol, and sulphamethazine from water using biochar, was also verified in many reports (Gu et al. 2021; Krasucka et al. 2021; Hoslett et al. 2021).

### Summary

Co-feeding (the combination of biochar and animal feed) is a promising incorporation technology. This section discussed the most important aspects of using biochar as a co-feeding material for animals like cattle, poultry, pigs, and fish. The effects of co-feeding with biochar on growth, gut microbiota, enteric methane production, egg yield, and endo-toxicant mitigations, as well as biochar's potential use as a litter amendment and for aquatic wastewater treatment, were thoroughly investigated. To conclude, our analysis demonstrated that significant value could be extracted through the use of biochar in animal farming applications, where the utilised biochar can then be further applied to soils for long-term storage while extracting additional agronomic value. The following key points, however, should be considered as potential topics for further research:

- There is an immediate need for research into the long-term toxicity of biochar in animals.
- Further research into the mechanisms of health improvement and toxicant/pathogen elimination by biochar co-feeding is required.
- Incorporating liquid manure storage with biochar can minimise greenhouse gas emissions, but more research is needed.
- The use of biochar as a litter and bedding-amendment agent requires more research before and after it is released into the environment.
- Full meta-analysis studies of biochar's application in livestock farms are necessary.

## Anaerobic digestion

The utilisation of biochar within the anaerobic digestion process prior to long-term storage, potentially in soils, is another value enhancement strategy where various technical benefits can be extracted. Anaerobic digestion, a promising bioprocess for converting organic feedstocks into biomethane-rich gas, has been used to manage biomass and produce biogas (Zhao et al. 2021b). However, several challenges have limited the widespread adoption of this technology. Low methane efficiency, impurities such as hydrogen sulphide, high carbon dioxide release, operational instability, and unsatisfactory substrate degradation, for example, all negatively affect biogas recovery potential. As a result, operating efficiency must therefore be improved, and biogas production must be upgraded and maximised.

Interestingly, biochar addition has been proposed as an effective and promising strategy for improving the treatment efficacy and operational stability of the anaerobic digestion process (Chiappero et al. 2020). Biochar supplementation has been demonstrated to alleviate inhibitors, enhance microbial activity, shorten the operational lag phase, and improve electron transmission between acetogens and methanogens (Chiappero et al. 2020; Pan et al. 2019). Biochar supplements, in particular, enhanced biogas generation by 22–40% and reduced the lag time by 28–64%. In addition, the abundance of methanogens and electro-trophic microorganisms were increased by 24.6–43.8% (Wang et al. 2018a). Compared to graphene, single-walled carbon nanotubes, and other carbon-based compounds, biochar is the most economically viable material since it can be made from waste feedstocks (Wang et al. 2021b).

Similarly, biochar properties, which include enhanced porosity, large specific surface area, an abundance of functional groups, and an exceptional electron transfer capacity, provide it with an advantage over other substances in terms of enhancing the anaerobic digestion process (Kumar et al. 2021c). The physicochemical properties of biochar could be simply optimised during its production through the optimal selection of feedstock and processing conditions, such as pyrolysis temperature and residence time (Kumar et al. 2021c).

Biochar production and subsequent use in anaerobic digestion can potentially have significant environmental benefits (Kumar et al. 2021d). Additionally, the direct addition of biochar to anaerobic digestion systems without the need for infrastructure upgrades adds another economic benefit, increasing biochar's popularity and potential in anaerobic digestion applications (Rasapoor et al. 2020). Overall, using the pyrolysis product (biochar) as an input to an anaerobic digestion system would meet zero waste goals, ensuring material flow, energy conversion and recovery, gaseous emission reduction, soil preservation, and a circular economy (Feng and Lin 2017).

This section reviews research on the utility of biochar in the anaerobic digestion process. The specific objectives of this section are to (i) investigate the physicochemical properties of biochar for use in the anaerobic digestion process, (ii) recognise the potential for biochar addition to anaerobic digestion operations to improve stability and upgrade biomethane production, (iii) assess the potential for biochar addition to remove certain contaminants and impurities from anaerobic digestion systems, and (iv) introduce future work opportunities for biochar adoption and utilisation in anaerobic digestion systems.

### Biochar characteristics and unique capabilities for anaerobic digestion

#### Adsorption

The type of biomass used, and the pyrolysis conditions significantly affect the porosity, surface area, and internal structure of biochar, all of which influence its immobilisation and sorption capabilities (Zhao et al. 2021b; Cantrell et al. 2012). The specific surface area correlates directly with pyrolysis temperature; as such, a higher pyrolysis temperature increases the specific surface area of biochar (Pandey et al. 2020). The adsorption and immobilisation capabilities are therefore enhanced as a result of the high specific surface areas and extensive porosity. The specific surface area of biochar can be significantly enhanced (Windeatt et al. 2014), for example, increasing from 0.92 m^2^ g^−1^ of manure-derived biochar at 350 °C to 187 m^2^ g^−1^ at 700 °C (Lü et al. 2018).

**Fig. 4:**
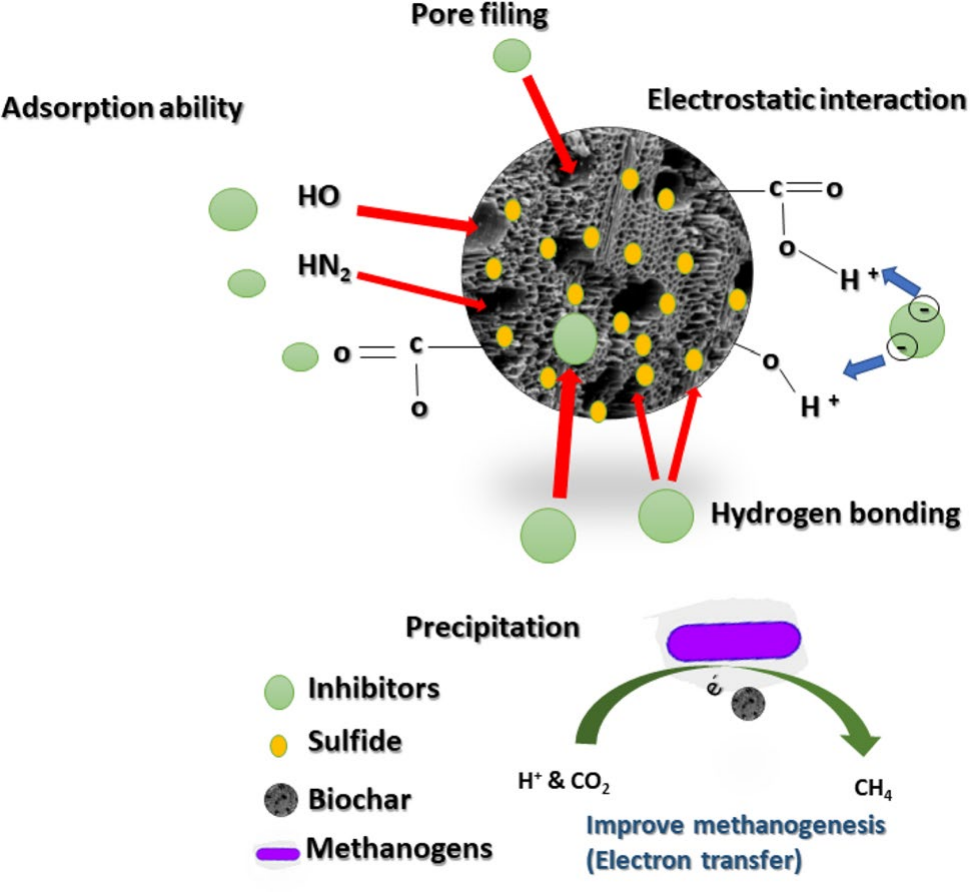
Biochar's unique properties, including adsorption, precipitation, bonding, electrostatic interaction, and pore filling, enable biochar to remove digestion inhibitors such as ammonia and sulphide

The exceptional sorption capacity of biochar enables the mitigation of contaminants such as ammonia, sulphide, and other inhibitors (Fig. 4), which inhibit anaerobic digestion methanogenesis (Antonangelo et al. 2021). Such inhibitors are adsorbed onto the surface of biochar via precipitation, electrostatic attraction, or ion exchange (Ambaye et al. 2021), where the hydroxyl, carbonyl, carboxylic, and amine groups present in the generated biochar are essential for the inhibitor to be removed (Ambaye et al. 2021). Thus, frequent biochar application promotes adsorption of the generated metabolites, thereby mitigating their unfavourable effect and improving the performance and stability of anaerobic digestion operations.

Additionally, the immobilisation value of biochar benefits the survival of entire microbes; for example, it was demonstrated that each biochar pore could contain between 10 and 100 methanogens (Lü et al. 2016); thus, a linear relationship between biochar addition and optimal methane production during anaerobic digestion was established(Qin et al. 2020). Numerous studies have established that methanogenic biota can easily persist in biochar pores (Pytlak et al. 2020). Sequencing analysis revealed that their variety was greater in biochar-added bioreactors than in control bioreactors (Chen et al. 2021a), enhancing gas generation potential.

#### Buffering

The feedstock properties and the pyrolysis conditions are the primary factors affecting the alkalinity and pH of biochar (Li et al. 2019b). Almost all biochar produced exhibited an alkaline characteristic; however, certain biomass, such as sawdust, presented neutrally to acidic pH biochar (Nzediegwu et al. 2021). By increasing the pyrolysis temperature, the pH and alkalinity of the resulting biochar are increased simultaneously (Fidel et al. 2017). This occurs due to the disintegration of fibre components (hemicellulose, cellulose, and lignin) into alkaline minerals, such as inorganic alkalis and carbonates (Panahi et al. 2020). Additionally, the higher the heating rate of pyrolysis, the more alkaline the biochar (Nzediegwu et al. 2021).

In this context, biochar's great buffering capacity has been demonstrated to overcome acidic and/or alkaline conditions often encountered during anaerobic digestion operations due to the high concentration of acidic and/or alkaline functional groups and metal ions. The fundamental rational for the ability of biochar to counteract acidic and/or alkaline disorder is due to functional groups involving carboxylic, amine, and phenolic groups being formed during the pyrolysis process. Additionally, the metal ions present in biochar, such as potassium and sodium, as well as other "earth" metals, particularly calcium and magnesium, contribute to the maximisation of the buffering value of biochar (Zhao et al. 2021b).

Various authors demonstrated that the addition of biochar improved the stability and neutral capacity of the anaerobic digestion system (Ma et al. 2020), which resulted in an increase in the maximum methane generation rate and a decrease in the lag time during anaerobic digestion of biowaste. For example, it was reported that the addition of 10 g L^−1^ of substrate biochar to thermophilic semi-continuous anaerobic digestion of food waste improved the pH value from 7.7 to 8.2 and mitigated the formation of total volatile fatty acids due to its buffering capacity (Lim et al. 2020). Similarly, a decreased lag phase of up to 44% and a 25% increase in methane productivity were reported as a result of the buffering effect of the 15 g L^−1^ addition (Ma et al. 2020).

In general, the buffering capacity of biochar can be determined using the process reactions presented in Eqs. (1–4), where C_x_H_y_COOH represents volatile fatty acids (Zhao et al. 2021b; Wang et al. 2017b).

1 $${\text{NH}}_{{4}}^{ + } + {\text{OH}}^{ - } = {\text{NH}}_{3} \cdot {\text{H}}_{2} {\text{O}}$$

2 $${\text{CO}}_{2} + {\text{H}}_{2} {\text{O}} = {\text{HCO}}_{3}^{ - } + {\text{H}}^{ + }$$

3 $${\text{C}}_{{\text{x}}} {\text{H}}_{{\text{y}}} {\text{COOH}} + {\text{NH}}_{3} \cdot {\text{H}}_{2} {\text{O}} = {\text{C}}_{{\text{x}}} {\text{H}}_{{\text{y}}} {\text{COO}}^{ - } + {\text{NH}}_{4}^{ + } + {\text{H}}_{2} {\text{O}}$$

4 $${\text{Ca}}\left( {{\text{Mg}}} \right){\text{CO}}_{3} + {\text{C}}_{{\text{x}}} {\text{H}}_{{\text{y}}} {\text{COOH}} \rightleftharpoons \left[ {{\text{C}}_{{\text{x}}} {\text{H}}_{{\text{y}}} {\text{COO}}} \right]_{2} {\text{Ca}}\left( {{\text{Mg}}} \right) + {\text{H}}_{2} {\text{O}} + {\text{CO}}_{2}$$

#### Electron delivery

The exceptional electron transfer ability of biochar has been linked to its electrochemical functional groups (phenazine, pyridine, phenolic, and quinone) and associated π-electron onto biochar aromatic groups (Wang et al. 2018b). Additionally, biochar was identified as a material capable of accelerating direct interspecies electron transfer (Wang et al. 2021b). Recent findings indicate that the quinone and hydroquinone functional groups contribute to the advancement of electron transfer between electron-donating acetogens and electron-accepting methanogens (Wang et al. 2021c). Additionally, biological elements, such as electron shuttles and cytochromes, may facilitate electron transmission between biochar and electro-trophic microbes.

The ability of biochar to transfer electrons is dependent on the type of feedstock and the pyrolysis temperature (Zhao et al. 2021b). Specifically, the enhancement of biochar's electron delivery capability is linearly related to the increase in pyrolysis temperature due to the following factors: (i) the formation of a conductive graphite-like network and numerous carbon sheets at a higher pyrolysis temperature increases the biochar conductivity and electron transfer capability (Chacón et al. 2020); (ii) the corresponding increase in the carbon concentration in the biochar enhances its ability for electrical conductivity (Gabhi et al. 2017); (iii) the increasing proportion of hybridised carbon molecules in biochar, combined with the development of the π-electron, accelerate electron transfer (Klupfel et al. 2014); and ultimately (iv) the formation and recoupling of oxygen-donating functional groups may improve the aromatic function and electron transfer potential with increased temperature (Zhang et al. 2019a).

However, it has been reported that disintegrating the oxygen-containing working group into carbon dioxide, carbon monoxide, and water decreased the electron transfer ability at high pyrolysis temperatures (Zhang et al. 2019a). Therefore, graphitised structures and functional groups in biochar are critical for increasing the material's electron transfer capabilities. Thus, optimising biochar pyrolysis conditions is important to enhance electron transfer potential during anaerobic digestion.

### Biogas upgrade and purification

#### Carbon dioxide removal

The raw biogas produced by anaerobic digestion is mostly composed of methane (45–70%), carbon dioxide (35–50%), hydrogen sulphide (0.1–4%), and water vapour, with trace amounts of other gases (ammonia, oxygen, and nitrogen) depending on the composition of the biomass influent (Kapoor et al. 2020; Ghosh et al. 2019). The presence of carbon dioxide, hydrogen sulphide, and other gaseous contaminants in biogas limits its industrial application for renewable energy and cooking and reduces its calorific value. For example, purified methane has a calorific potential of 55.6 × 10^3^ MJ m^−3^, compared to 21.9 MJ m^−3^ for raw biogas (Ascher et al. 2019). As a result, the application of biogas has frequently been constrained by the cost of upgrading and purifying equipment (Li et al. 2017a). After refinement, upgraded biogas can be pumped into the public gas system, used as a transportation fuel, or converted further into electricity and heat via a combined heat and power unit (Salman et al. 2019). Electricity can be sold to provide producers with additional financial benefits, depending on each country's policies (Sambusiti et al. 2013).

To maximise the efficiency of biogas upgrading, some contaminants such as hydrogen sulphide and carbon dioxide should be removed from raw biogas prior to application (Miltner et al. 2017; Sun et al. 2016). Traditionally, conventional methods such as water scrubbing, membrane separation, physicochemical absorption, and cryogenic separation have been employed to remove gaseous contaminants from biogas (Miltner et al. 2017; Sun et al. 2015). Typically, upgrading technologies incur energy and economic expenses equal to or greater than 50% of total methane generation (Petersson and Wellinger 2009; Browne et al. 2011). Biochar has been recognised as a carbon dioxide adsorbent for both in situ (biochar added during anaerobic digestion) and ex situ applications (Sethupathi et al. 2017). As shown in Table 6, the sorption capacity of carbon dioxide on biochar can reach hundreds of mg g^−1^, which is significantly greater than that of other adsorbents such as activated carbon; thus, biochar has been considered to be an effective adsorbent for carbon dioxide removal and biogas improvement due to its porosity and increased surface area. The many functional groups and alkalinity characteristics of biochar, in particular, may promote the chemical sorption of carbon dioxide (Saha and Kienbaum 2019).

**Table 6:** Potential role of biochar in biogas upgrading

Biochar biomass	Pyrolysis condition	Specific surface area (m^2^ g^−1^)	Porous size (cm^3^ g^−1^)	Impurity removed	Removal rate (mg g^−1^)	References
Rice straw	312.5 °C for 20 min	122.2	0.083	Carbon dioxide	57.5	Huang et al. (2015)
Rice husk	550 °C for 30 min	263.4	0.209	Carbon dioxide	138.2	Chiappero et al. (2021)
Sawdust	700 °C for 60 min	773	0.32	Hydrogen sulphide	54.8	Ma et al. (2021)
Maple wood	500 °C for 10 min	161	0.095	Hydrogen sulphide	219	Choudhury and Lansing (2020)
Pig manure	500 °C for 4 h	47.4	–	Hydrogen sulphide	59.6	Xu et al. (2014)
Food Waste and sludge	700 °C for 30 min	220.5	0.099	Hydrogen sulphide	66.6	Hervy et al. (2018)
Corn stover	500 °C for 10 min	23.5	0.011	Hydrogen sulphide	238	Choudhury and Lansing (2020)

Biogas can be upgraded by incorporating biochar into the anaerobic digestion system. Various kinds of biomass with varying properties can be used to produce biochar, which could then be successfully incorporated into anaerobic bioreactors. The removal efficiency of gaseous contaminants varies depending on the type of biochar utilised

Similarly, various surface modifications and combinations have been proposed to enhance the upgrading performance and carbon dioxide removal efficiency of biochar, including element loading, chemical activation, and functional group amendment (Dissanayake et al. 2020; Tan et al. 2017). For instance, the carbon dioxide adsorption efficiency of ultrasound-stimulated biochar was roughly ninefold that of untreated biochar (Dissanayake et al. 2020). Pelaez-Samaniego et al. (2018) reported that sodium carbonate-impregnated biochar increased its hydrogen sulphide scrubbing capacity by 37%. Notably, exhausted biochar can be regenerated (Farooq et al. 2018; Tan et al. 2015) or used as a valuable soil improvement material for sulphur-deficient soils (Sawatdeenarunat et al. 2016; Zhang et al. 2016a). For the purpose of atmospheric carbon removal, exhausted biochar should preferably be applied to soils for secured long-term storage, however, potential contamination should be assessed. Thermal regeneration should be avoided as to eliminate any further carbon emissions.

Additionally, biochar can significantly increase methane production and reduce the lag time associated with organic material degradation, as illustrated in Table 7. This positive effect on anaerobic digestion operations are a result of the biochar's properties and functional groups, which enable it to remove inhibitors, sustain pH, and accelerate direct interspecies electron transfer (Qi et al. 2021a). Biochar may potentially be used as a carrier for hydrogenotrophic methanogens to convert carbon dioxide to methane. Sethupathi et al. investigated the effect of four distinct biochar types derived from various biomass sources (perilla leaf, soybean stover, Korean oak, and Japanese oak) on carbon dioxide removal in synthetic biogas containing 40% carbon dioxide and 60% methane (Sethupathi et al. 2017). The researchers determined that several types of biochar effectively removed carbon dioxide with the optimal removal achieved using perilla based biochar, although methane was not removed (Sethupathi et al. 2017).

**Table 7:** Performance of anaerobic digestion systems with varying types of biochar

Biochar materials	Pyrolysis temperature	Feedstock	Biochar concentration	Anaerobic digestion performance	References
Wheat straw, fruitwood, and chicken manure	350, 450, 550 °C	Chicken manure	5%	Enhanced methane production by 69% at 550 °C	Pan et al. (2019)
Manure	350 °C	Air-dried manure	10 g L^−1^	Diminished lag phase and enhanced methane yield by 35.71%	Jang et al. (2018)
Fruitwoods	800 °C	Food waste	0.25–2.5 g per g total solids	Lowered lag phase by 36.3–54.0%; inoculum to substrate ratio at 0.8–2 improved the maximum methane generation aspect by 100–275%; Enhanced organic matter degradation	Cai et al. (2016)
Pine sawdust	650 °C	Food waste	8.3–33.3 g L^−1^	Declined lag phase by 36–41%; Increased hydrogen and methane yield by 31% and 10%, respectively; Increased volatile fatty acid consumption	Sunyoto et al. (2016)
Forest waste	450 °C	Sludge	0.5–12.0 g per g total solids	0.8 and 3.7 g per g VS improved methane generation by 192–61% during the first 16 d of digestion	Cimon et al. (2020)
Wood-pellets mixed with timber waste	800 °C	Poultry litter	100% poultry litter total solids	Decreased lag phase by 41%; Improved maximum daily methane generation by 136%	Indren et al. (2020)
Rice straw	260 °C	Dead pig carcass	2–10 g L^−1^	Improved biogas yields up to 61–91%	Xu et al. (2018)

Biochar additives derived from a variety of biomass sources have the potential to increase biogas production and reduce the lag time required to reach the peak of methane production during the anaerobic digestion process. The optimal dosage may be 10 g L^−1^ (w/w) of substrates

Direct utilisation of in situ corn stover biochar in anaerobic digestion improved performance, increased biomethane quality to more than 90% and eliminated carbon dioxide to the extent of up to 86% (Shen et al. 2015). Linville et al. (2017) reported that in situ addition of 0.96–3.83 g per gVS of fine biochar enhanced the methane content to 77.5–98.1% and eliminated 40–96% of the generated carbon dioxide when compared to non-biochar control reactors operating at different thermophilic and mesophilic temperatures, respectively. Therefore, adding biochar to anaerobic digestion systems would significantly increase process stability and performance while significantly lowering the cost of biogas upgrading.

#### Mitigating of hydrogen sulphide release

Biogas contains hydrogen sulphide in concentrations ranging from 0.1 to 2.0% (v/v) depending on the feedstock type (Shanmugam et al. 2018). Hydrogen sulphide gas is corrosive to metal pipelines and is frequently hazardous to humans (Zhao et al. 2021b). The porous nature and large surface area of biochar are critical for hydrogen sulphide adsorption (Table 6). One gram of biochar applied to an anaerobic bioreactor can lower hydrogen sulphide emissions by approximately 78% (Wang et al. 2019b). Hydrogen sulphide adsorbs onto basic biochar (Sahota et al. 2018), as demonstrated by a significant pH reduction following hydrogen sulphide adsorption (Sahota et al. 2018). Several studies observed a decrease in the surface area and porosity of biochar following hydrogen sulphide sorption, confirming that the sorption efficiency of biochar is primarily induced by physical sorption onto its porous surface and is relatively associated with chemical oxidation (Sahota et al. 2018; Xu et al. 2014; Shang et al. 2013).

Xu et al. investigated the ability of two biochars derived from sewage sludge and pig manure to mitigate hydrogen sulphide emissions. Under the same conditions, biochar derived from pig manure exhibited a greater capacity for hydrogen sulphide adsorption than biochar derived from sewage sludge (Xu et al. 2014). Kanjanarong et al. (2017) reported that adding biochar to a continuous stirred tank bioreactor eliminated more than 98% of hydrogen sulphide at a pH of 7.98. The researchers determined that H_2_S adsorption was primarily due to the hydroxide and carboxylic groups present on the biochar (Kanjanarong et al. 2017). Additionally, an experiment conducted in an aerobic environment indicated that hydrogen sulphide and oxygen could diffuse into the pores of biochar following their dissolution in the water film (Xu et al. 2014). Moreover, oxygen reacts with the dissolved hydrosulphide ions to form elemental sulphur, with further catalytic oxidation to sulphate, facilitated by the presence of certain metals such as sodium, potassium, iron, and magnesium.

### Enhancing the performance of anaerobic digestion operations

#### Alleviating volatile fatty acid accumulation and buffering potential

The stability of the anaerobic digestion system is critical to the technology's performance. Accumulations of volatile fatty acids would shift anaerobic digestion systems into an unstable state. The acetogenic and methanogenic consortiums often correct the volatile fatty acid production pattern by converting volatile fatty acids to methane and carbon dioxide (Zhang et al. 2014b). However, organic overloads of easily biodegradable feedstocks accelerate the synthesis of volatile fatty acids, resulting in acidic conditions and potentially anaerobic digestion failure due to increased hydrogen production (Li et al. 2020b; Ren et al. 2018).

Additionally, excessive synthesis of volatile fatty acids inhibits methanogenesis. Several treatment technologies and approaches have been proposed to address this issue, including constructing a bioreactor with easily biodegradable influent and pH buffering additives (Zhang et al. 2018b). To compensate for the pH drop, co-digestion with a substrate with a high buffering capacity has been employed (Gong et al. 2020). However, it remains a significant challenge to establish a stable, simple, and cost-effective strategy for developing the buffering capability of anaerobic digestion systems. Biochar is proposed as a viable option due to the following reasons: (i) it can be delivered via an environmentally friendly and cost-effective approach, and (ii) its physicochemical properties can be optimised to operational conditions (Chiappero et al. 2020; Fagbohungbe et al. 2017). Thus, biochar can be employed effectively as a promising additive to enhance the breakdown of volatile fatty acids.

Biochar's buffering capacity is primarily determined by various fundamental reasons listed in Eqs. (1–4) (Chiappero et al. 2020). First, the lower pH value caused by the formation of volatile fatty acids can be offset by some functional groups in the biochar, where the amine group adsorbs hydronium ion and acts as an electron acceptor media. Biochar's exceptional buffering capability against the formation of volatile fatty acids in anaerobic digesters operating at a higher organic loading rate was previously reported (Ma et al. 2020). Second, the inorganic metals contained in biochar, such as calcium, magnesium, potassium, sodium, silicon, iron, aluminium, and sulphur, can contribute to the biochar's alkalinity characteristics (Zhang et al. 2018b). In particular, the presence of earth elements, particularly magnesium and calcium, and other metals such as potassium and sodium, as well as active functional groups within the biochar, is a critical aspect for sustaining its buffering function (Wang et al. 2017b).

Jang et al. examined the effect of biochar produced from dairy manure on anaerobic digestion at temperatures of 20, 35, and 55 °C. The researchers observed reduced volatile fatty acid volumes and higher methane output in all investigated settings, owing to the presence of alkali metals, such as calcium and magnesium, and biochar's alkalinity potential (Jang et al. 2018). Due to the abundance of alkaline metals and functional groups in vermicompost-derived biochar, Wang et al. observed that it improved the buffering capacity of the anaerobic digestion system fed with high organic loads of chicken manure and kitchen waste (Wang et al. 2017b).

Wei et al. discovered that 3500–4700 mg L^−1^ calcium carbonate alkalinity increased methane yield and total solids removal from sludge in corn stover-biochar-added digesters (Wei et al. 2020a). Ambaye et al. (2020a) demonstrated an increase in methane generation and total volatile fatty acids elimination when fruit waste anaerobic digestion was supplemented with sludge-derived biochar. Finally, biochar may accelerate the transfer of electrons between syntrophic bacteria and methanogens, hence increasing biogas production and system stability (Wang et al. 2021b, d). These electro-trophic microbes may be successfully enhanced via the regular use of biochar; additionally, direct interspecies electron transfer may improve volatile fatty acid breakdown (Wang et al. 2021b, d). Additionally, the electron transfer mechanism between bacteria and archaea facilitates the syntrophic conversion of many organic particles to methane (Lovley and Science 2011).

In summary, as compared to the direct interspecies electron transfer syntrophic ability and the action of functional groups on the biochar surface, the attractive buffering capability of biochar is necessary to correct volatile fatty acid accumulation during the anaerobic digestion operation (Wang et al. 2017b). Even so, the mechanisms underlying biochar's ability to alleviate volatile fatty acid inhibition remain unknown. Moreover, additional research is necessary to determine the maximum biochar dosage associated with these features. Similarly, the significance of porous biochar in promoting the development of microbial biofilms and its protective capacity to enrich functional microorganisms attached to it, under acid stress, should be investigated.

#### Mitigating ammonia inhibition

Ammonia, namely ammonium and free ammonia nitrogen, collectively referred to as total ammonia nitrogen, has long been seen as a critical constraint that adversely affects or even causes anaerobic digestion failure (Rajagopal et al. 2013). Chen et al. (2008) observed that a total ammonia nitrogen concentration of 1.7–14 g L^−1^ could lower methane emissions by up to 50%. Additionally, free ammonia nitrogen is considered the principal inhibitor of methanogens (Rajagopal et al. 2013) and has been shown to have an inhibitory effect on anaerobic microbes at concentrations ranging from 150 to 1200 mg L^−1^ (Poirier et al. 2017).

Numerous mitigation strategies have been proposed to resolve the detrimental effects of total ammonia nitrogen and free ammonia nitrogen on anaerobic digestion. Strategies mainly focus on the immobilisation and removal of such inhibitors, which include, co-digestion, dilution, and microbial adaptation (Yun et al. 2016; Dai et al. 2017); struvite precipitation (Huang et al. 2014); the use of a microbial desalinisation cells (Zhang and Angelidaki 2015); in situ membrane treatment (García-González et al. 2016), and ammonia stripping (Kinidi et al. 2018; Georgiou et al. 2019). Other options include the use of inorganic adsorbents such as zeolites (Cardona et al. 2021), as well as organic adsorbents such as activated carbon (Poirier et al. 2017) and biochar (Ambaye et al. 2021).

Biochar can effectively mitigate ammonia inhibition, enhance methane production, shorten reactor lag times, and offer reactor stability (Table 8). Su et al. (2019) demonstrated that adding biochar to the anaerobic digestion of food waste can alleviate inhibition caused by 1500 mg L^−1^ total ammonia nitrogen. Similarly, Lü et al. (2016) reported that biochar can stabilise anaerobic digestion under high ammonium stress of 7000 mg-nitrogen L^−1^.

**Table 8:** Utilisation of biochar to alleviate stressors in anaerobic digestion systems

Biochar	Pyrolysis conditions	Feedstocks	Biochar dosage	Inhibitor	Fermentation pattern	References
Wheat straw	550 °C	Food waste and sludge	10 g L^−1^	Volatile fatty acids (> 4000 mg L^−1^)	Propionate reduced to 1460 mg L^−1^; specific methane yields improved to 24%	Kaur et al. (2020)
Sewage sludge	350 °C	Fruit waste and activated sludge	0.5, 1, 1.5, and 2 g L^−1^	Volatile fatty acids (2587 mg L^−1^)	Total volatile fatty acids volume were reduced to 387, 1196, 1465, and 1594 mg L^−1^ for biochar additions, respectively	Ambaye et al. (2020a)
Sewage sludge				Volatile fatty acids (2943 mg L^−1^)	Total volatile fatty acids volume reduced to 1196, 1821, 646, and 1142 mg L^−1^ for biochar additions, respectively	Ambaye et al. (2020a)
Sawdust waste	500 °C for 1.5 h	Food waste and sludge	20 g L^−1^	Volatile fatty acids (57,900 mg COD L^−1^)	– Butyrate decreased up to 70% – Methane content boosted over 70%	Wang et al. (2021d)
Vermicompost	500 °C for 2.0 h	Chicken manure and kitchen waste	24 g L^−1^	Volatile fatty acids (> 12,000 mg L^−1^)	Total volatile fatty acids reduced from 10,798 to 3957 mg L^−1^	Wang et al. (2017b)
Wood chips	800 °C	Food waste	5 g L^−1^	Volatile fatty acids (> 3000 mg L^−1^)	Methane outcome boosted by 18%	Lim et al. (2020)
Rice husk	550 °C for 2 h	Corn stover and chicken manure	10 g L^−1^	Total ammonia nitrogen (> 6300 mg L^−1^)	Methane generation aspect enhanced by 28–96%	Yu et al. (2021b)
Macadamia nutshells	350 °C for 2 h	Food waste	33.3 g L^−1^	Ammonia nitrogen (1500 mg L^−1^)	Chemical oxygen demand removed up to 90%, compared with 8% without biochar	Su et al. (2019)
Fruitwood	800–900 °C	Glucose solution	10 g L^−1^	Ammonia (7 g-N L^−1^)	Peak methane generation rate prompted by 47.1%	Lü et al. (2016)
Pine pellets	Gasification 600–710 °C	Primary sludge	2.49 and 4.97 g per gTS sludge	Ammonia nitrogen (400–450)	– Increased methane yield by 3.9–9.5% – Increased ammonia nitrogen quantity by 67% for anaerobic system without biochar compared with by −7.2 to 4.7% with biochar	Shen et al. (2016)
Corn stover	Gasification 850 °C	Primary sludge + waste activated sludge	0.25, 0.375, 0.5, and 1 g per d	–	Enhanced methane content by 13.7–25.3%	Shen et al. (2017)
Wheat bran pellets	Pyrolysis 800 °C	Wheat bran pellets	25 g L^−1^	Ammonia nitrogen (200–250)	– Increased total volatile fatty acids removal and reduced lag phase – No adsorption of ammonia by biochar	Viggi et al. (2017)
Wheat straw	Pyrolysis 350, 450, and 550 °C	Chicken manure	5% w/w	Total ammonia nitrogen (4.48 g L^−1^)	Total ammonia nitrogen reduced by 25% than the control	Pan et al. (2019)

The biochar produced at various pyrolysis temperatures, particularly those between 500 and 900 °C, can alleviate several inhibitors that have a detrimental effect on the anaerobic digestion process. Specifically, volatile fatty acids, ammonia nitrogen, and total nitrogen levels may decrease following biochar addition, indicating that biochar plays a role in alleviating anaerobic digestion inhibitors and improves overall digestion performance

The following hypotheses were made regarding the positive effects of ammonia mitigation mechanisms using biochar: (i) directly via cation-exchange capacity (Shen et al. 2017); affordable surface functional groups and physical or chemical adsorption capability (Shen et al. 2017; Khalil et al. 2018; Sarkhot et al. 2013); and (ii) indirectly via improved direct interspecies electron transfer (Lü et al. 2016; Chen et al. 2021a); and immobilisation of microorganisms (Lü et al. 2016). The findings summarised in Table 8 demonstrate how biochar can be used to enhance gas production by removing total ammonia nitrogen and free ammonia nitrogen from the fermentation system. For example, when biochar was added to the anaerobic reactor, 5.5 mg g^−1^ of total ammonia nitrogen was adsorbed (Khalil et al. 2018; Sarkhot et al. 2013). The effectiveness of biochar in reducing ammonia levels was shown to be correlated with its surface area (Zhai et al. 2020). The abundant functional groups (carboxylic, phenolic, and lactonic) produce acidified-biochar (0.1–3.0 mmol g^−1^), which is necessary for free ammonia nitrogen sorption and reactor buffering capabilities (Pan et al. 2019; Chun et al. 2004). Generally, the size of the biochar particles had a significant effect on the improvement of total ammonia nitrogen and free ammonia nitrogen suppression. The large particle size of 2–5 mm demonstrated the greatest ammonia inhibition alleviation and maximal biomethane generation in an apparently shorter lag time, but the smaller-sized biochar of 75–150 μm had a significantly lower effect (Lü et al. 2016).

Furthermore, the microporosity of biochar may provide an ideal habitat for microbial colonisation, which may aid in the recovery of free ammonia nitrogen accumulation (Cheng et al. 2020a). *Bacteroidetes*, *Chloroflexi*, *Synergistetes*, and *Planctomycetes* have all recently been promoted in biochar-added bioreactors (Chen et al. 2021a). Additionally, biochar supplementation can abate the inhibition of acidogenic by-products by increasing syntrophic oxidation of volatile fatty acids (Zhao et al. 2021c). In addition to acidified bacterial enrichment, the porous characteristics of biochar also facilitate the growth of methanogens such as *Methanosaeta*, *Methanobacterium*, *Methanosarcina*, and *Methanolinea* (Kumar et al. 2021c; Chen et al. 2021a). Interestingly, an increase in methanogenesis enzymatic activity, such as coenzyme F420, was observed in a biochar-added reactor (Qi et al. 2021b).

### Contaminant removal by biochar in the anaerobic process

Inhibitors often cause the decline of biomethane production and instability of anaerobic digestion operations through shifting the microbial consortia or inhibiting microbial growth (Zhang et al. 2021a). In general, inhibitors can be direct, such as metals and organics (antibiotics, chlorophenols, lignocellulose hydrolysate, pesticides, and halogenated aliphatics); or indirect, such as sulphides, hydrogen, long-chain fatty acids, volatile fatty acids, and ammonium (Fagbohungbe et al. 2017).

#### Heavy metals removal

Heavy metals can either enhance or impair anaerobic digestion operations. Numerous metals, including nickel, molybdenum, and cobalt, have been shown to enhance the activity of anaerobic enzymes (Luo et al. 2020). For example, nickel is an essential component of hydrogenase, coenzyme F430, and carbon monoxide dehydrogenase, all of which aid in the growth of anaerobic microbes such as methanogenic archaea and sulphate-reducing bacteria (Khan et al. 2021b). According to Pobeheim et al. (2010), a nickel/cobalt mixture or nickel alone enhanced methane output by 30 and 15%, respectively. Similarly, Cao et al. demonstrated that 100 mg kg^−1^ of copper could accelerate the decomposition of organic matter in a digestion system, resulting in increased methane production (Cao et al. 2015). The increased methane volume is a result of enhanced metal bioavailability, enhancing microbial growth and metabolism (Pobeheim et al. 2010).

On the contrary, heavy metals can inhibit enzymes by replacing metals in enzymes or altering the functional groups of protein molecules (Chen et al. 2014). Additionally, heavy metals may produce cytotoxic compounds (Zayed and Winter 2000) that may permeate the cell wall and inhibit microorganisms, hence reducing the amount of methane produced during the fermentation process (Shi et al. 2020). The inhibitory effect of heavy metals on the anaerobic digestion system is relative to their concentration, which regulates enzymatic synthesis (Liu et al. 2021a). Therefore, the half-maximal inhibitory concentration method is employed to quantify the detrimental effect of heavy metals on anaerobic digestion operations (Chen et al. 2014). However, the concentration values for heavy metals in the literature varied significantly. For example, the half-maximal inhibitory concentration values for cadmium, nickel, zinc, and chromium in anaerobic digestion were 330, 1600, 270, and 250 mg L^−1^, respectively (Lin and Chen 1999), whereas the values for the elements mentioned above were reported to be considerably lower (Altaş 2009). Such variation is probably attributable to the inconsistency in anaerobic digestion processes, including reactor configurations, feedstock type, and operational parameters.

Heavy metals are primarily classified according to their bioavailability into three groups: those bound to carbonates or iron/manganese oxides, exchangeable, and those bound to organic particulates (Qi et al. 2021a). Humic acid, an organic component of anaerobic slurry, has the potential to change heavy metal speciation, thereby passivating metals. Wang et al. reported that humic acid with oxygen and aromatic functional groups could adsorb and react with copper(II), nickel(II), and cobalt(II) via anaerobic digestion of corn stover and chicken manure (Wang et al. 2021e). Enhancing metal passivation could be accomplished by optimising anaerobic digestion practices, such as pH, redox potential, and temperature, as well as by adding certain materials, such as biochar (Tao et al. 2021). Whereby the bioavailability and immobility of zinc and copper were diminished as a result of carbonate formation and sulphide precipitation during anaerobic digestion of swine slurry (Marcato et al. 2009). Substrate pH reduction to 5 may be effective in improving the sorption of lead(II) (Naiya et al. 2009).

**Fig. 5:**
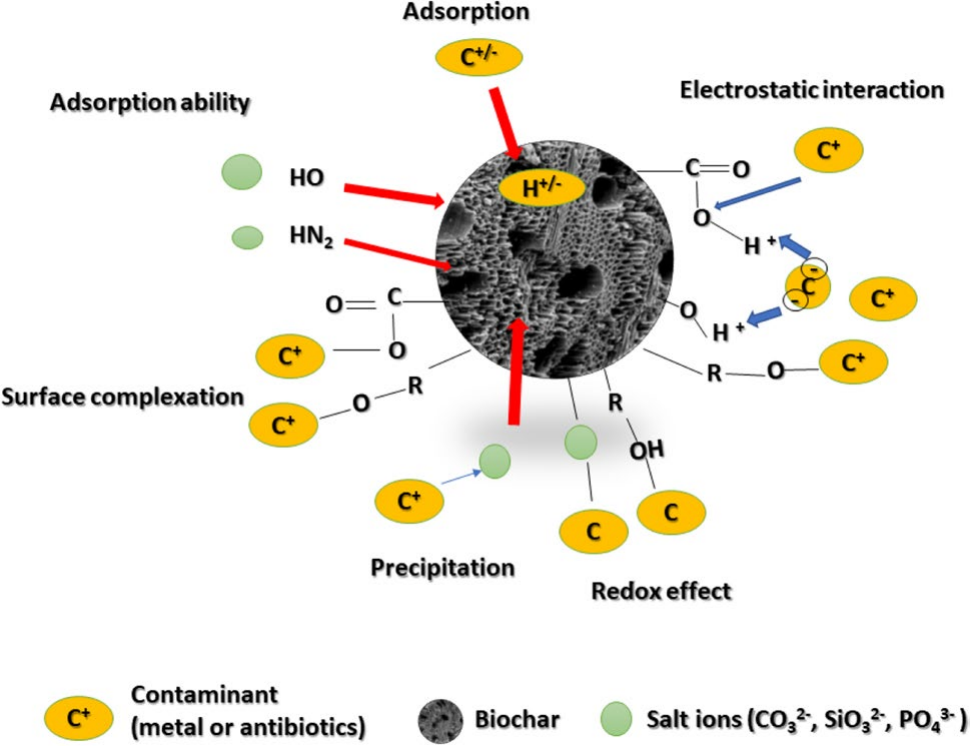
Many features of biochar allow to remove pollutants such as heavy metals and antibiotics. Contaminants can be passivated via adsorption, surface complexation, redox effect, ion exchange, precipitation, electrostatic attraction, and π–π interactions during anaerobic digestion. The heavy metal pollutants affecting the anaerobic digestion process could reduce either mesophilic or thermophilic operation system, which verifies the wide aspect of biochar efficiency on heavy metal removal at various digestion conditions

Biochar has the ability to passivate non-biodegradable heavy metals, hence lowering their bioavailability (Liu et al. 2018b). Several studies have established the role of biochar in improving heavy metal passivation via chemisorption, complexation, redox effect, ion exchange, precipitation, electrostatic attraction, physical adsorption, and π–π interactions during anaerobic digestion (see Fig. 5 and Table 9) (Gopinath et al. 2021; Wang et al. 2019a). This is attributed to the inherent characteristics of biochar, including a large surface area, porosity, adsorption sites (e.g. carboxyl organics and hydroxyl), and large surface cations (Ahmad et al. 2014).

**Table 9:** Impact of biochar addition to anaerobic digestion on heavy metals stabilisation

Digested feedstock	Biochar condition	Fermentation temperature	Main effect	References
Sewage sludge	Pyrolysis, 600 °C Potassium permanganate-biochar	35 °C	Chromium, cadmium, nickel, zinc and copper, were reduced to 4.8%, 8.8%, 15.5%, 7.6%, and 9.7%, respectively	Li et al. (2019c)
Sewage sludge	Pyrolysis, 600 °C Manganese iron oxide-biochar	35 °C	Carbonate-bound chromium, cadmium, and nickel were reduced by 49.8%, 49.5%, and 49.8%, respectively	Zhang and Wang (2020)
Pig manure	Particle size less than 0.425 mm	25 °C	Exchangeable nickel, lead, arsenic, manganese, and chromium decreased by 6.5%, 12.3%, 12.9%, 3.1%, and 22.1%, respectively	Wang et al. (2021f)
Organic Municipal Solid Waste	Pyrolysis-gasification process	51–53 °C	Lead, mercury, copper, nickel, chromium, and zinc content in the slurry was reduced	Bona et al. (2020)

Digested feedstocks such as sewage sludge, pig manure, organic municipal solid waste were investigated, and the impact of biochar preparation conditions along fermentation temperature was provided

Mahdi et al. confirmed that adsorption via ion exchange could remove 57–72% of iron(II), nickel(II), and copper(II) using date seed biochar (Mahdi et al. 2018). However, biochar's passivation ability is typically regulated by operational practices such as alkalinity, organic loading rate, and pH. The alkaline nature of biochar enhances the adsorption and precipitation capacity of heavy metals, hence increasing the amount of biogas produced during anaerobic digestion (Mao et al. 2015). Recently, biochar modification has been proposed as a viable method to attain highly effective and steady performance in the reduction of heavy metals. For example, thiol-improved biochar derived from rice straw was able to alleviate heavy metals in soil, reducing Pb^2+^ by 11.1–39.2% via complexation (Fan et al. 2020).

#### Antibiotics

Antibiotics of various kinds and dosages can inhibit anaerobic digestion by increasing volatile fatty acid accumulation and decreasing methane yield (Table 10). For example, clarithromycin dosages ranging from 0 to 1000 mg kg^−1^ total suspended solids have been shown to increase total volatile fatty acids values by 10–22% in the anaerobic digestion of sewage sludge (Huang et al. 2019a). Similarly, Hu et al. reported a 73.2% increase in total volatile fatty acids when sulphamethazine was added to the digestion system (Hu et al. 2018). Antibiotics have been shown to inactivate or inhibit microbiota by deteriorating the cell membrane and deoxyribonucleic acid transcript (Finberg et al. 2004); thus, the antibiotic-induced accumulation of volatile fatty acids may disrupt the cell wall and extracellular polymeric matrix. Due to their slower growth rate, archaea are more susceptible to such unfavourable effects than bacteria, as shown by their lower methane generation (Aydin et al. 2015). Liu et al. (2021b) demonstrated that tetracycline at a concentration of 8 mg L^−1^ reduced daily methane generation by 73%.

**Table 10:** Influence of antibiotics on methane outcome during the anaerobic digestion process

Feedstock	Antibiotic	Dosage	Methane production	References
Dewatered sludge	Roxithromycin	0–1 mg L^−1^	Reduced from 0.164 L per gVS to 0.151 L per gVS	Ni et al. (2020a)
Swine manure	Chlortetracycline	0–0.5 g per kg TS	Reduced by up to 37.3%	Yin et al. (2016)
Swine manure	Oxytetracycline		Inhibited gas yield by 8.3–22.1%	Yin et al. (2016)
Dewatered sludge	Sulphadimethoxine	100 mg L^−1^	Increased methane output by 39–52%	Zhi et al. (2019)
Dewatered sludge	Oxytetracycline		Enhanced methane output by 1–25%	Zhi et al. (2019)
Cattle manure	Ceftiofur	0.2–250 mg L^−1^	Reduced methane output by 20% at dosage > 50 mg L^−1^	Flores-Orozco et al. (2020)
Cattle manure	Chlortetracycline	0.04–1.28 mg L^−1^	Reduced methane output by 12–33% at concentration > 0.64 mg L^−1^	Andriamanohiarisoamanana et al. (2020)
Cattle manure	Oxytetracycline		Reduced methane yield by 20–32%	Andriamanohiarisoamanana et al. (2020)

The table demonstrates that the existence of most antibiotics can reduce the anaerobic digestion performance due to their inhibitory effect on microorganisms

In comparison with heavy metals, antibiotics are easily adsorbed by biochar (Fig. 5) via non-covalent π–π interfaces and electrostatic attraction, with the sorption efficiency of biochar being entirely dependent on the matrix pH (Gopinath et al. 2021). The alcohol, carboxyl, phenyl, hydroxyl, and aldehyde groups contained in the biochar are critical concerning antibiotic adsorption (Wu et al. 2019a), as these groups accept an electron to form π–π electron donor–acceptor (adsorption) with antibiotics (Ahmed et al. 2018). Moreover, large amounts of enzymes and microorganisms bound to the biochar surface via biofilm formation are necessary for the transformation and decomposition of antibiotics in anaerobic digestion processes (Liang et al. 2020). The higher sorption function of biochar (Fig. 5) facilitates its enhanced adsorption capabilities as compared to graphite, multiwalled carbon nanotubes, bentonite, and activated carbon (Ahmed et al. 2015). However, additional research is necessary to determine the biotic and abiotic factors contributing to biochar advanced antibiotic removal within the fermentation system. Additionally, the large-scale application of biochar for antibiotic removal remains a challenge that requires further investigations.

#### Microplastics

The effects of microplastics on the anaerobic digestion process are highly variable, depending on their type, particle size, concentration, and anaerobic digestion conditions (Table 11). Microplastics, particularly nano-plastics, can diffuse through the membranes of microbial cells, disrupting proteins and phospholipids (Zhang and Chen 2020). For example, 200 mg L^−1^ nano-plastics may impair the activities of *Gracilibacteraceae*, *Cloacamonaceae*, and *Anaerolinaceae* during anaerobic digestion, resulting in a 14.4% reduction in methane yield (Fu et al. 2018a). Adding microplastics (polyvinyl chloride) at a concentration of 10–60 mg per gTS to sewage sludge resulted in considerable bisphenol A leakage, which lowered methane production by 75.8–90.6% (Wei et al. 2019a). On the other hand, Chen et al. (2021b) discovered that microplastics enhanced the release of several important enzymes, including protease, butyrate kinase F420, and butyrate kinase, resulting in the production of volatile fatty acids and methane. Such variation is attributed to the fact that microplastics have a variety of properties. Microplastics contain a variety of surface functional units, such as carboxylic, phenyl, and amine, which serve as absorbents for some contaminants (antibiotics) and serve as a colonisation set for microorganisms, exerting a variety of effects on the operation of anaerobic digestion (Cao et al. 2021).

**Table 11:** Microplastics' effects on biogas production during anaerobic digestion

Microplastics	Concentration	Particle size	Digested feedstock	Mode	Effect on biogas yield	References
Polyvinyl chloride	10 particles	1000 μm	Waste activated sludge	Batch	Improved methane yield to 5.9%	Wei et al. (2019a)
	20–60 particles per g total solids				Reduced methane output by 9.4–24.2%	
Polyethylene	10–60 particles per g total solids	40 μm	Waste activated sludge	Batch	No effect	Wei et al. (2019b)
	100 and 200 particles per g total solids				Reduced methane output by 12.4% and 27.5%	
	200 particles per g total solids			Continuous	Reduced methane output by 28.8%	
Polyester	1–200 particles per g total solids	200 μm	Waste activated sludge	Batch	Reduced methane output by 4.9–11.5%	Li et al. (2020c)
Polyamide	5–50 particles per g total solids	500–1000 μm	Waste activated sludge	Batch	Enhanced methane output by 4.84–39.5%	Chen et al. (2021b)
Polystyrene	0.05–0.20 g L^−1^	5 μm, 80 nm	Synthetic wastewater	Batch	No effect	Zhang et al. (2020a)
	0.25 g L^−1^				Reduced methane output by 17.9% and 19.3%	
Polystyrene	10 μg L^−1^	50 nm	Synthetic wastewater	Batch	No effect	Wei et al. (2020b)
	20 and 50 μg L^−1^				Reduced methane output by 19.0% and 28.6%	

The presence of microplastics in the anaerobic digestion system induces different effects on system performance. Higher doses of microplastics can reduce the digestion performance; however, lower doses may advance gas production due to the formation of micro-sheets for biofilm formation that improves microbial colonisation and proliferation

Through sorption and/or implied microbial biodegradation, biochar can alter microplastics in the media. Magnetic biochar (iron (II, III) oxide -biochar) has been shown to be effective at immobilising microplastic particles in groundwater and soil (Tong et al. 2020). Additionally, by enhancing the oxidation process, the applied magnetic-biochar enhanced the separation of microplastics from other contaminants, such as heavy metals (Ye et al. 2020). This separation process was possibly facilitated by the effective sorption of microplastics and biochar with the contaminants. In addition to the adsorption process, biochar can be used to stimulate the microbial niche during anaerobic digestion, hence aiding in the degradation of micro/nano-plastics. Biochar, in particular, can improve direct interspecies electron transfer in anaerobic digestion operations by promoting the growth of *Pseudomonas stutzeri* and *Pseudomonas putida*, both of which are known as effective plastic biodegrading bacteria (Qi et al. 2021b).

Additionally, biochar can enhance functional microbes, such as methanogens, to accelerate direct interspecies electron transfer by diminishing the gap between syntrophic compartments (Zhao et al. 2015). Li et al. (2021) demonstrated that promoting direct interspecies electron transfer and enhancing the formation of microbial consortiums can promote pyrene, a polycyclic aromatic hydrocarbon, biodegradation in anaerobic sludge digestion. Similarly, Hao et al. (2021) noted that the addition of magnetic biochar could stimulate some species such as *Spirochaetes*, *Euryarchaeota*, and *Chloroflexi*, which accelerate the methanogenesis phase and promote the elimination of polycyclic aromatic hydrocarbons. Recirculation of biochar-loaded digestate could potentially improve the stability and variety of the microbial community, hence accelerating polycyclic aromatic hydrocarbons biodegradation. This would be an interesting area for future research.

#### Furfural and 5-hydroxymethyl furfural degradation

5-hydroxymethyl furfural, a toxic furan by-product, has the potential to have a detrimental effect on anaerobic digestion systems. They could be formed by pretreatment of substrates with heat, acid, or alkaline (Kim and Karthikeyan 2021; Bruni et al. 2010). At lower concentrations, 5-hydroxymethyl furfural can be metabolised to methane during fermentation. Khan et al. (2021c) reported that using 5-hydroxymethyl furfural and furfural as a carbon source resulted in a methane yield of 21.31–28.98 mL per 0.5 g chemical oxygen demand. Huang et al. (2019b) demonstrated that various furfural and 5-hydroxymethyl furfural concentrations in acid pretreated elephant grass could be completely consumed during the subsequent digestion process. However, it was demonstrated that furfural and 5-hydroxymethyl furfural concentrations of more than 1 and 2 g L^−1^ significantly reduced the methane yield to 24.7 and 47.6%, respectively, due to deoxyribonucleic acid and cell membrane structure damage to the entire microbe population during the digestion process (Anburajan et al. 2020).

Only a few studies have been conducted to determine the effectiveness of biochar in removing/adsorbing 5-hydroxymethyl furfural during the anaerobic digestion process. However, their removal with biochar was documented. For example, Li et al. (2014) demonstrated that adding 75 g of biochar per L of water can adsorb 10 g per L furfural with a 100% removal efficiency. Similarly, biochar that has been mildly treated with ruthenium can work as a catalyst to promote the conversion of furfural to furfuryl alcohol under conditions of high pressure and temperature (Bardestani et al. 2020). However, additional research is necessary to fully understand the potential of biochar in removing 5-hydroxymethyl furfural and other furan by-products during anaerobic digestion operations.

### Summary

To conclude, our analysis shows that biochar can be utilised as a means of enhancing anaerobic digestion operations, primarily for biogas generation and upgrading, operation performance and sustainability, and the mitigation of inhibitory impurities such as gaseous impurities, antibiotics, residues, heavy metals, microplastics, and furan-by-products. Such enhancement is attributed to the biochar's fundamental properties, including its large surface area, porosity, surface functional groups, and interaction with an anaerobic microbial consortium enabling sophisticated electron transfer. Nonetheless, the following challenges and prospects continue to be significant concerns:

- Further research is needed to elucidate the underlying function of biochar in improving the adsorption of pollutants, particularly furan derivatives, antibiotic resistance genes, and nano-plastics, which are emerging environmental threats.
- Biochar effectively removes contaminants in anaerobic digestion systems, while also adding value to the digestate produced. This approach would enhance contaminant removal while maintaining the proper quality of digestate biofertilizer for agricultural applications.
- Several batch tests with various feedstocks and the addition of biochar have been conducted under anaerobic digestion conditions. However, few large-scale anaerobic digestion system tests have been conducted to ascertain the optimal dosage, substrate to inoculum ratio, particle size, and re-utilisation rates. Thus, the field-scale application of biochar for improving anaerobic digestion should be researched in the future. Furthermore, additional research should focus on developing innovative tools and biochar composites, such as metal biochar frameworks and biochar-loaded nanomaterials, to enhance biochar's sorption capacity.
- While practically all prior research established that adding biochar to anaerobic digestion operations would considerably enhance methanogenesis, certain studies revealed the unfavourable or even inhibitory effects of adding biochar on anaerobic digestion performances. The precise mechanism by which this inhibition occurs is unknown. Thus, optimising the condition of biochar in fermentation systems requires further clarification of the appropriate inhibition mechanisms.

## Composting

### Concepts of the composting process

The composting process generally consists of three distinct phases: (i) a moderate-temperature (mesophilic) stage, (ii) a high-temperature (thermophilic) stage, and (iii) a cooling and maturation stage (Xiao et al. 2017). At various stages of composting, distinct microbial communities dominate (Awasthi et al. 2017a). Composting substrate characteristics such as moisture content, particle size, biochemical composition and properties, and environmental conditions such as oxygen supply (aeration), temperature, and hydrogen ion concentration (pH) all have a significant effect on the composting process (Onwosi et al. 2017; Viaene et al. 2017).

The following summarises the effects of the factors mentioned above on composting: (i) the initial feedstocks' low carbon to nitrogen ratio may result in nitrogen losses due to leaching or ammonia volatilisation, whereas their higher carbon to nitrogen ratios mostly lengthen the composting period (Diaz et al. 2011); (ii) the biochemical characteristics of compost indicate its degradability, and thus some feedstocks are difficult to degrade during the process(Vandecasteele et al. 2017); (iii) small-sized substrates promote the development of clumps, while large-sized substrates are difficult to decompose (Bernal et al. 2009); (iv) moisture contained within compost promotes nutrient and gas exchange within the substrates and compost pile (Onwosi et al. 2017); (v) the pH of the compost pile has an effect on all microbial activities and ammonia volatilization (Zhang and Sun 2017); (vi) while the air temperature is critical for composting to initiate, the temperature of the compost pile reveals the extent of microbial activity and advancement of the composting process. A high temperature is advantageous for destroying pathogens and weed seeds (Xiao et al. 2017). Finally, aeration is a critical parameter that significantly impacts the quality of compost, composting processes, and greenhouse gas emissions, so adequate aeration of compost is vital (Chowdhury et al. 2014).

### Production of co-composted biochar

Biochar co-composting refers to the process of mixing biochar with compostable substrates such as manure, plant residues, and sewage sludge prior to aerobic composting (Antonangelo et al. 2021). Because co-composted biochar retains all of the compost and biochar properties, it is primarily used to improve soil conditions and mitigate toxic elements (Khan et al. 2016). The methods by which biochar is applied in soils are critical in determining its anticipated effects.

In general, two techniques are widely used to combine biochar and compost. The first is to add biochar to composted substrates after the composting process is complete and prior to soil application. This approach may improve soil nutrient availability and plant growth (Cao et al. 2018; Naeem et al. 2018; Safaei Khorram et al. 2019). The second approach is the addition of biochar to substrates at the start of the composting process, which is referred to as co-composting. The co-composted biochar would then be applied to soils. The cost of co-composting is significantly less than the cost of mixing biochar with compost post-composting, which requires two distinct steps: conventional composting and subsequent biochar addition (Khan et al. 2016). The co-composting procedure has several advantages, which will be discussed in detail.

### Changes of compost properties after biochar addition

#### Physicochemical properties of biochar during composting

Temperature is a critical parameter because it not only affects the rate of composting but also contributes to the inhibition of pathogenic microbes and weeding seeds originating from the input substrates (Czekała et al. 2016). When biochar was added with substrates, the composting temperature increased rapidly as compared to the non-supplemented control (Wei et al. 2014). Additionally, it has been observed that co-composting with biochar facilitated process completion one week earlier compared to the process without biochar addition (Li et al. 2015; López-Cano et al. 2016; Godlewska et al. 2017). The higher temperature achieved with biochar addition has been attributed to the increased microbial activity induced by biochar and lowered heat losses (Li et al. 2015), where biochar attempts to fill intraparticle voids of composted substrates, which are believed to reduce heat losses during the composting process (Zhang et al. 2016b). Additionally, biochar enhances aeration, which accelerates microbial growth and increases the amount of heat generated (Zhang et al. 2016b).

In addition to temperature, moisture content plays a significant role in influencing the effectiveness of composting. The ideal moisture level should remain between 50–60% throughout the composting process (Li et al. 2015). The high moisture content of the compost pile may restrict aeration flow within the composted substrates, whereas low moisture levels inhibit microbial activity (Godlewska et al. 2017). As the composting process is initiated, the increased temperature of the pile leads to significant water evaporation. However, some studies have demonstrated that adding biochar to compost reduces water loss compared to compost without biochar (Xiao et al. 2017; Li et al. 2015). This is mainly due to biochar's exceptional water holding capacity (Husni and Samsuri 2012). Biochar is recommended to be added to compost in general, but especially to substrates with high moisture levels, to bring the moisture content down to an acceptable level.

The pH of the compost is a critical parameter. The ideal pH range for plant growth is between 6.5 and 7 (Zhang et al. 2016b). Additionally, pH affects the immobility of heavy metals, as higher pH values of compost reduce the solubility of metal contents, lowering the toxicity of heavy metals to crops when applied as a fertiliser to the soil (Zhang et al. 2016b). The addition of biochar can lead to an increase in the pH of the compost as a result of the leaching of soluble alkaline groups (Antonangelo et al. 2021). Co-composting wheat straw, biochar, and poultry droppings resulted in a higher pH of the subsequent compost than without the addition of biochar (Czekała et al. 2016).

Similarly, Czekała et al. discovered that co-composting sewage sludge and biochar (4% w/w) increased pH considerably as compared to sewage sludge composted without biochar supplementation (Czekała et al. 2016). Sánchez-Garcia et al. (2015) reported that adding biochar at a concentration of 3% (dry weight) to barley straw and poultry manure mixtures increased the pH from 5.9 to 6.5. However, in some cases, the pH was reduced when co-composting organic material and biochar due to increased acid generation from organic particulate biodegradation as a result of increased microbial activity (Wei et al. 2014; Chen et al. 2010). Additionally, the increased microbial activity may result in nitrogen biodegradation and increased ammonia generation during the later stages of composting. The generated ammonia/ammonium may then be adsorbed by the surface of biochar, resulting in a slight decrease in the pH of the compost mixture (Antonangelo et al. 2021). On the other hand, various studies have demonstrated that co-composting with biochar did not significantly alter the pH of the co-composted mixture (Zhang et al. 2014c, 2016b). In general, the pH value of biochar-amended compost varies according to the biochar characteristics, and composting substrates mentioned previously.

#### Impact of biochar amendment on the composting of organic matter

Research has shown that co-composting with biochar resulted in a higher dissolved organic carbon degradation rate than composting without biochar (Zhang et al. 2016b; Sánchez-García et al. 2015; Khan et al. 2014). This can be explained as follows: to begin, biochar addition enhances microbial growth by increasing oxygen flow due to the enhanced porosity of the biochar, which further enhances the substrate mixture's porosity (Vandecasteele et al. 2017). Second, the excellent surface properties of biochar facilitate the sorption of ammonium, ammonia, and hydrogen sulphide produced by the compost's microbial activities (Antonangelo et al. 2021). Additionally, the existence of functional groups on the surface area of biochar chemisorbed dissolved organic carbon, resulting in a reduction in leaching losses (Zhang et al. 2014c).

Ultimately, the addition of biochar can stabilise humic and fulvic acids during the composting process by improving the aromatic properties of organic materials, thereby improving the compost quality (Awasthi et al. 2017a; Jindo et al. 2016). Biochar indirectly facilitates the biodegradation of organic matter by simulating enzymatic activity (Zhang et al. 2016b). Jindo et al. (2016) reported that adding biochar to compost stabilised the chemical component of fulvic acids and enhanced the degradation of humic acids, resulting in higher environmental stability. Wei et al. (2021) evaluated woody peat and biochar co-composting. They revealed that co-composting reduced the humic to fulvic acid ratio compared to the control, indicating that the humification process is optimised following co-composting.

There are very few studies on the effect of co-composting with biochar on reducing organic pollutants. Polycyclic aromatic hydrocarbons are the most reported pollutant in sewage sludge compost. However, biochar addition can help diminish such contaminants in composts (Stefaniuk and Oleszczuk 2016). The reduction in polycyclic aromatic hydrocarbons is attributed to the increased surface area of the biochar and subsequent increase in microbial activity (Oleszczuk and Kołtowski 2018).

#### Influence of biochar addition on some nutrients

While inorganic nitrogen is frequently transferred from ammonium to nitrate during composting (Larney et al. 2006), the bioavailability of nitrogen decreases as the composting process progresses. Biochar co-composts typically release less ammonia and more nitrate than composts without biochar (Malińska et al. 2014). The conversion of ammonia/ammonium into nitrate (nitrification process) is typically facilitated by nitrifying bacteria, which is enhanced by the addition of biochar, whereas the opposite process (ammonification process) is inhibited (López-Cano et al. 2016). Furthermore, the metal content of biochar (e.g. aluminium, iron, silicon, and iron-, calcium-, aluminium- phosphates, calcium- and magnesium- carbonates) may provide a positively charged biochar surface, enhancing nitrate adsorption (Archanjo et al. 2017).

Losses of nitrogen were reduced when co-composting with biochar due to the sorption of ammonium/ammonia on the biochar surface (López-Cano et al. 2016; Chen et al. 2010). Specifically, López-Cano et al. (2016) demonstrated that co-composting with biochar increased NO_3_^−^ levels by twofold compared to composting without biochar addition. Other scholars reported similar results (Khan et al. 2014; Kammann et al. 2015). Co-composting municipal solid waste and green waste (50:50, w/w) with 10% biochar obtained from holm oak pyrolysed at 650 °C increased the ammonium-nitrogen content of compost by up to 29% after 90 days of composting (Viaene et al. 2017; Vandecasteele et al. 2017). As a result, biochar-amended compost contains more nitrogen than control compost without biochar, which benefits the soil. It was stated that using biochar at 5% (w/w) increased the nitrogen content of the final compost by 45% compared to compost without biochar (Jain et al. 2018).

Importantly, composting incorporates additional critical nutrients such as phosphorus, calcium, potassium, sodium, magnesium, and sulphur. The concentrations of these metals highly depend on the type of composted substrate, with phosphorus and potassium contributing the most. The leaching of macro-elements from compost reduces the fertiliser quality of compost significantly (Rameeh 2012). Co-composting with biochar has been shown to affect the macro-elements and fertiliser properties of the compost (Zhang et al. 2016b). Biochar produced from wheat straw that was pyrolysed at 500–600 °C and added at a rate of 10% or 15% (w/w) to pig manure and wheat straw compost increased the amount of phosphorus, calcium, magnesium, and potassium elements in the compost (Zhang et al. 2016b). The higher content of such elements in the co-compost is attributed to the macro-elements being retained in the biochar added.

Additionally, the biochar's negatively charged surface area retained the potassium, magnesium, and calcium cations via electrostatic interactions (Zhang et al. 2016b; Vandecasteele et al. 2016). As a result, ions contained in the composted substrates were not leached out, increasing the quality of fertilisers produced. Additionally, the authors noted that the phosphate levels (after 105 days) in compost substrates containing biochar were reduced by 2.6–23.8% compared to their concentration prior to composting. This was attributed to phosphorus loss (soluble form) caused by organic phosphorus mineralisation and microbe utilisation. The levels of sodium ions were nearly unchanged before and after the composting process with biochar addition and were 8.7–16.7% lower than the control, indicating that soil deterioration may potentially be reduced (Zhang et al. 2016b).

Wei et al. (2021) observed that the total phosphorus concentration increased during the co-composting operation of woody peat and biochar due to the biodegradation of organic materials. However, the authors noted that the total phosphorus content of the co-compost could not be increased above that of control. Vandecasteele et al. (2017) reported that co-composting municipal solid waste and green waste (1:1 w/w) with biochar (10% at 650 °C) decreased the HNO_3_-P content by 18%, however, increased the H_2_O- and CaCl_2_-P bioavailability by 16.8 and 4.9%, respectively, in biochar-amended substrates. The authors concluded that adding biochar had no effect on the fertiliser value of phosphorus.

#### Influence of biochar on heavy metals availability

Biomass contains varying amounts of heavy metals, depending on the type of biomass. Composting may influence the bioavailability of heavy metals. Typically, bonds between metals and organic particulates are formed via complexation, which reduces the solubility of metals and thus their bioavailability. However, when compost is added to the soil, with the variations in pH, the availability of heavy metals can rise (Godlewska et al. 2017). Several studies examined the stabilising effects of biochar on heavy metals during composting, which were attributed to biochar's ability to reduce the bioavailability and immobility of metals. Chen et al. (2010) reported a decrease in the mobility of zinc and copper in co-composted pig manure incorporating biochar. Additionally, biochar added to pig manure and humic acid compost has been shown to passivate lead, copper, and cadmium by 66, 95, and 69%, respectively (Zhou et al. 2018a).

There are several mechanisms by which biochar deactivates heavy metals. For example, complexation, physical adsorption, reduction, ion exchange, electrostatic interactions with the biochar surface, and precipitation can potentially affect metal bioavailability and immobilisation (Guo et al. 2020a; Wang et al. 2017c; Awasthi et al. 2016). Heavy metals may form complexation bonds with biochar functional groups (carboxylic groups); additionally, surface precipitation may immobilise metals, particularly lead (Xue et al. 2012). Cui et al. (2016a) observed a reduction in the bioavailability of copper, zinc, and arsenic when co-composting sawdust and chicken manure (2:3, w:w) with biochar (5%) produced from rice straw or fungal biomass combustion when compared with the control in which biochar is not added. The decreased bioavailability of heavy metals was due to the effective immobilisation imposed by biochar.

Li et al. (2015) investigated the effect of co-composting pig manure with biochar made from pyrolysed corn stalk prepared at various temperature (250–900 °C) on the availability of copper and zinc. Their findings indicated that biochar produced via pyrolysis at temperatures between 450 and 500 °C had the greatest reduction in heavy metal bioavailability. After 90 days of composting, the bioavailability of these metals was reduced by 24.8 and 9.9%, respectively, when compared to their initial concentrations at the start of the composting process. However, some studies indicate that biochar did not affect the bioavailability and mobility of heavy metals in co-composts containing biochar (López-Cano et al. 2016).

In conclusion, the efficiency of reducing the bioavailability of heavy metals depends entirely on the original compost substrates and the characteristics of the biochar used. Biochar acts as a beneficial core element during composting, immobilising metals in the co-compost. After land application, the leaching (elution) or metal mobility rate would be considerably lowered. As a result, their leaching into the environment will be significantly reduced.

#### Influence of biochar on the release of gases during composting

Several gases are produced during the composting process as organic matter degrades, including carbon dioxide, methane, nitrous oxide, carbon monoxide, and ammonia (Cui et al. 2016a; Yin et al. 2021a). The gases produced are primarily greenhouse gases, which have a detrimental effect on the ecosystem and contribute to global warming. As a result, reducing the amount of gaseous emissions produced by the composting process would benefit climate change. Biochar may be beneficial for gaseous emission reduction when compared to unamended compost, due to improved aeration, decreased bulk density, gas diffusion, and enhanced growth of methane-consuming methanotrophic archaea (Godlewska et al. 2017).

##### Impact on carbon dioxide emissions during composting

There is conflicting evidence regarding the effect of co-composting with biochar on carbon dioxide emissions. According to some studies, adding biochar to pig manure composting operations significantly reduced carbon dioxide emissions by 26.1% (Wang et al. 2018c). Similarly, when co-composting, carbon dioxide emissions were reduced by 51% compared to when no biochar was added. These studies attributed the reduction in carbon dioxide emissions to biochar's adsorption capacity for carbon dioxide (Vandecasteele et al. 2017; Vandecasteele et al. 2016). On the other hand, Mao et al. demonstrated that adding biochar to the same substrates increased carbon dioxide emissions by 53.2% (Mao et al. 2018). The various effects of biochar on carbon dioxide emissions have been attributed to either biochar improving organic particulate sequestration and therefore reducing carbon dioxide emissions during co-composting (Liu et al. 2017b), or biochar enhancing microbial growth and intensifying their ability to further biodegrade organic materials, thereby liberating more carbon dioxide (Mao et al. 2018). Another potential source of increased carbon dioxide liberation is increased aeration in compost piles caused by biochar, which inhibits methanogens' activity and denitrifies bacteria, thereby increasing methane to carbon dioxide conversion (He et al. 2019).

##### Impact on methane emissions during composting

Biochar addition can improve the compost environment by reducing anaerobic zones and providing an oxidation redox, thereby decreasing anaerobe activity and increasing methanotroph activity, thus lowering methane emissions (Yin et al. 2021a; Sonoki et al. 2013). Additionally, the ammonium/ammonia adsorption capacity of biochar would reduce methanogen nitrogen utilisation, thereby reducing methanogen activity and methane emissions (Karhu et al. 2011). Awasthi et al. (2016) co-composted wheat straw and sewage sludge (1:1) with wheat straw biochar (12%). The authors discovered an up to 80% reduction in methane emissions when compared to compost without biochar addition. Similarly, when biochar produced from a mixture of hardwood and softwood was added in a 4:1 ratio to chicken manure at a 27% (dry weight) concentration, methane generation was reduced by up to 32% compared to compost without biochar addition (Chowdhury et al. 2014).

##### Impact on nitrous oxide emissions during composting

Biochar can significantly reduce nitrous oxide emissions by decreasing the amount of inorganic nitrogen used by nitrifying/denitrifying bacteria (He et al. 2019). Additionally, this can be achieved via the adsorption capacity established by the biochar's surface area, which adsorbs nitrous oxide and converts it to nitrogen via a specific biological reaction (Harter et al. 2016). Biochar addition may also affect specific enzymes encoded by certain genes that affect nitrous oxide production, which are involved in the denitrification process, such as the nirK, nirS, and nosZ gene expressions (Xiao et al. 2017; Yin et al. 2020a). Wang et al. investigated the effect of co-composting wood chips, pig manure, and sawdust with bamboo biochar (3% at 600 °C) on nitrous oxide emissions. The authors observed a reduction of 25.9% in nitrous oxide emissions compared to the control. This reduction was attributed to a decrease in the amount of nitrogen dioxide, which is primarily converted to nitrous oxide. Furthermore, by enhancing bacterial activities that reduce nitrous oxide and inhibit the following enzymatic activities (Wang et al. 2013). Other studies confirm these findings (Chowdhury et al. 2014; Awasthi et al. 2016).

##### Impact on ammonia emissions during composting

Ammonia gas can be released during the composting process. Ammonia immobilisation can potentially reduce ammonia emissions while also retaining ammonia, which helps reduce nitrogen losses, thereby increasing the compost's fertiliser value. Efforts have been made thus far to minimise ammonia losses during the composting process. The addition of biochar to compost is capable of reducing ammonia release, and loss due to the following: (i) modified surface area of biochar can adsorb ammonia/ammonium through the acidified functional groups (Godlewska et al. 2017); (ii) adding biochar improves the compost's overall environment and stimulates the activity of nitrifying bacteria, which convert ammonia to nitrate, thereby retaining nitrogen in the compost (Akdeniz 2019); (iii) biochar addition to compost increases certain enzymatic activities, such as cellulase (Yin et al. 2021b), which increase dissolved organic carbon production through cellulose disintegration, thereby reducing ammonia emissions via increased microbial utilisation of ammonium (Agyarko-Mintah et al. 2017).

Khan et al. (2014) demonstrated that co-composting with biochar for 126 days reduced ammonia emissions by 90% compared to composts without the addition of biochar. Similarly, Steiner et al. observed that co-composting biochar, obtained from pine chips at 400 °C, with poultry litter, resulted in a 47% reduction in ammonia emissions (Steiner et al. 2010). However, some studies found that co-composting wheat straw and sewage sludge with biochar increased nitrogen losses (ammonia) from the compost, when compared to compost without biochar (Awasthi et al. 2016). Malińska et al. (2014) investigated the effect of co-composting wood chips and sewage sludge with biochar on ammonia emissions and discovered that biochar had a dose-dependent effect on ammonia emissions. Whereas adding biochar during the first composting week reduced ammonia emissions relative to the control due to biochar's adsorption capacity, and adding biochar during the second composting week increased ammonia emissions relative to the compost without biochar. Similarly, a positive correlation between biochar dosage and ammonia release during sewage sludge composting was discovered (Hua et al. 2009).

In conclusion, Table 12 summarises the effects of biochar addition on the greenhouse gas emissions produced by various composting processes. This effect varied significantly between studies due to the variety of biochars used and operating conditions such as pyrolysis temperature, dosage, and particle size (Li et al. 2015; He et al. 2019; Awasthi et al. 2020; Chen et al. 2017b).

**Table 12:** Influence of biochar addition on gaseous emissions during the composting process

Composting substrates	Biochar features				Reduction in gaseous emissions (%)				References
	Type	Dosage (%)	Pyrolysis temperature	Particle size (mm)	Carbon dioxide	Methane	Ammonia	Nitrous oxide	
Sawdust and poultry manure	Straw	10 (w/w)	450–500 °C	≤ 2	Not mentioned	Not mentioned	12.4	Not mentioned	Zhang et al. (2020b)
Wheat straw and poultry manure	Bamboo	2–10 (dry weight)	Not mentioned	Not mentioned	5.5–72.6	12.5–72.9	19.0–77.4	12.4–81.6	Awasthi et al. (2020)
Chicken manure and wheat straw	Chicken manure	2–10 (dry weight)	550–600 °C	Not mentioned	Not mentioned	20.5–61.5	19.2–48.1	4.7–15.1	Chen et al. (2020c)
Sawdust and pig manure	Bamboo	5 (dry weight)	Not mentioned	2–3	Not mentioned	54.4	12.4	36.1	Mao et al. (2018)
Sawdust and layer manure	Cornstalk, bamboo, wood and layer manure	10 (dry weight)	450–500 °C	≤ 2	Not mentioned	15.5–26.1	9.2–24.8	Not mentioned	Chen et al. (2017b)
Wheat straw and sewage sludge	Wheat straw	2–18 (dry weight)	500–600 °C	2–5	Not mentioned	92.8–95.3	58.0–65.2	95.1–97.3	Awasthi et al. (2017b)
Sugarcane straw and poultry litter	Green waste and poultry litter	10 (dry weight)	550 °C	Not mentioned	Not mentioned	77.8–83.3	54.9–60.2	68.2–74.9	Agyarko-Mintah et al. (2017)
Solid sewage waste and green waste	Holm oak	10 (dry weight)	650 °C	Not mentioned	52.9	95.1	Not mentioned	14.2	Vandecasteele et al. (2016)
Rice chaff and cattle manure	Wheat straw	3 (dry weight)	450 °C	Not mentioned	Not mentioned	Not mentioned	Not mentioned	54.1	Li et al. (2016)
Barley straw and poultry manure	Hardwood and softwood (4:1)	27.4 (dry weight)	500–700 °C	≤ 16	21.5–22.9	77.9–83.6	35.3–43.0	16.1–35.3	Chowdhury et al. (2014)
Sawdust, pig manure and wood chips	Bamboo	3 (dry weight)	600 °C	Not mentioned	Not mentioned	Not mentioned	Not mentioned	25.9	Wang et al. (2013)

The addition of biochar significantly reduced the gaseous emissions produced during the composting process, thereby having a beneficial effect on the environment. The optimal dosage for adding biochar is approximately 10 g L^−1^

#### Influence of biochar on microbes during composting

During composting, biochar can act as a protective environment for microbes. The various pores in biochar, combined with its ability to retain nutrients, dissolved organic carbon, nitrogen, minerals, water-holding capacity, adequate moisture, pH buffering, and aeration, can provide an adequate environment for microbial proliferation. Additionally, it can mitigate inhibitor stresses such as hydrogen sulphide, ammonia, heavy metals, pathogens, and adverse environmental conditions (leaching, desiccation, and pH) (Sanchez-Monedero et al. 2018; Sun et al. 2020a; Sun et al. 2020b). Several studies have demonstrated that biochar-modified compost contains a greater diversity of bacteria, actinomycetes, and fungi than compost without biochar (Antonangelo et al. 2021; Wei et al. 2014; Du et al. 2019). Adding biochar at a rate of 5–20% to compost enhanced *Bacillus sp.* growth compared to those without biochar (Du et al. 2019). Similarly, compost containing 1% biochar increased the abundance of *Actinobacterium sp*., *Flavobacterium sp*., and *Rhizobiales sp*., resulting in improved lignocellulosic biomass decomposition. In doing so, it enhanced *Acinetobacter sp.* for hydrocarbon biodegradation and *Geobacillus thermodenitrificans* to improve denitrification and hydrolysis processes (Wei et al. 2014).

### Factors influencing the effect of biochar during the composting process

Various biochar characteristics have a significant impact on the effectiveness of the composting process. Specifically, the type of biochar, the rate at which it is loaded, the conditions under which it is produced, the pH of the biochar, and the particle size of the biochar (Antonangelo et al. 2021).

#### Biochar loading rates

The dosage of biochar used has a significant effect on the compost's properties and on the reduction in greenhouse gas emissions. Generally, a loading rate of 10–15% (w/w) is considered optimal for biochar addition, but rates up to 20–27% have been reported (Chowdhury et al. 2014; Steiner et al. 2010). For example, Awasthi et al. (2017b) discovered that the greatest effect of biochar added to compost occurred at a concentration of 12% (out of a range of 2–18%). Similarly, the 10% loading rate was estimated to have the greatest mitigating efficiency among dosages of 2–10% (Awasthi et al. 2020). A higher loading rate of biochar may result in compost dehydration due to excessive aeration.

#### Type of biochar

The type of biomass used to produce biochar has a significant effect on the characteristics of biochar; thereby, it directly affects the greenhouse gas emission potential and compost properties (Akdeniz 2019). Biochar porosity can improve the aeration of the compost environment, increase aerobic bacterial activity, particularly methanotrophs and nitrifying bacteria, and decrease anaerobic activity (e.g. methanogens and denitrifying bacteria). Furthermore, the biochar's surface area facilitates the adsorption of ammonium/ammonia and nitrous oxide. These aspects strongly support the ability of biochar to significantly reduce methane, ammonia, and nitrous oxide emissions from compost.

In the light of these characteristics and the type of biochar used in the composting process, biochar derived from plant and woody biomass has a higher capacity for mitigating methane, ammonia, and nitrous oxide emissions from compost. Various studies confirm this assertion. Chen et al. (2017b) for example, used a variety of biochar types derived from bamboo, corn stalk, coir, woody biomass, and manure in the composting process to mitigate ammonia and methane emissions. Their findings indicated that using biochar made from corn stalk resulted in the greatest reduction in gaseous emissions due to its high surface area, pore volume, and total acidic functional groups. Similarly, nitrous oxide and methane emissions were reduced by 42.01 and 19.79%, respectively, when bamboo biochar was used compared to rice straw biochar (He et al. 2019), which is attributed to the higher specific surface area and pore volume of the bamboo biochar, as well as its higher aromatisation degree.

#### Pyrolysis temperature

The physicochemical characteristics of biochar are mostly affected by pyrolysis temperature, which significantly influences biochar surface area, porosity, and aromatic groups. These characteristics can potentially be improved with the increase in pyrolysis temperature; however, the presence of acid oxygen-containing functional groups diminishes. As a result, biochar produced from the same material at different temperatures will have a different effect on greenhouse gas emissions mitigation during the composting process. In general, biochar produced via pyrolysis at temperatures ranging from 500 to 900 °C is more effective at reducing nitrous oxide and methane emissions, whereas biochar is produced via pyrolysis at temperatures ranging from 200 to 500 °C is more effective at reducing ammonia emissions (Yin et al. 2021a). Woody biomass such as beech, bamboo, and oak, as well as crop residues such as wheat straw, pyrolysed at a temperature between 400 and 700 °C, are typically used to make biochar for composting (Antonangelo et al. 2021; Zhang et al. 2016b), where biochar properties, such as specific surface area, presence of functional groups, porosity, alkalinity, and water holding capacity, among others, are optimal. Deng et al. (2021) reported that biochar produced at a pyrolysis temperature of 600 °C was more effective than biochar produced at 300 or 450 °C at reducing nitrous oxide emissions. However, Li et al. (2015) concluded that biochar formed at temperatures of 700 and 900 °C released more ammonia during composting than biochar formed at temperatures of 300 and 500 °C.

#### Biochar particle size

The particle size of biochar significantly affects its pore characteristics and specific surface area. A study reported that using biochar with a particle size of 4–10 mm reduced methane emissions by up to 56.8% during composting; however, by reducing the particle size to less than 1 mm, methane emissions increased by 22.15% (He et al. 2018a). This difference was attributed to the loss of aggregates caused by the use of smaller-sized biochar, which resulted in a looser compost pile. Additionally, it was difficult to form interconnections between biochar pores and pig manure compost substrates using smaller-sized biochar (He et al. 2018a). As a result, adding granular biochar increased the porosity of the compost, thereby reducing methane emissions. On the other hand, adding powdered (< 1 mm) biochar reduced ammonia emissions from pig manure compost more than granular (4–8 mm) biochar, owing to the increased presence of active sites on the surface of the powdered biochar; however, the size of the biochar had little effect on methane and nitrous oxide emissions(He et al. 2019).

### Effect of co-composted biochar on agricultural lands

Fertility loss, a decline in soil organic matter, soil nutrient imbalances, and unsustainable agricultural land use all contribute significantly to agricultural productivity loss (Antonangelo et al. 2021; Agegnehu et al. 2017). Additionally, the current climate change is causing a dramatic decline in global incomes, which is directly related to food scarcity (Kogo et al. 2021). As a result, proper land use combined with climate change mitigation is favoured. The application of biochar to soils benefits both climate change and land use by promoting carbon sequestration as well as agricultural development. As a result, recent efforts to improve the utilisation of biochar as a co-compost additive have gained increased interest.

Increased soil salinity is a major environmental concern that affects approximately 932 million hectares globally (Daliakopoulos et al. 2016). Co-composting with biochar may alleviate soil salinisation, as biochar improves the chemical and physical properties of salty soils by promoting salt leaching (Lashari et al. 2013). Additionally, using stable carbon-rich materials such as biochar, which is an emerging alternative to a variety of soil organic amendments, can help overcome soil mineralisation (Agegnehu et al. 2017). Thus, co-composting with biochar is a viable strategy for improving soil fertility, organic carbon, and crop yields (Agegnehu et al. 2017; Yuan et al. 2017a). Co-composting with biochar can provide nutrients and improve nutrient utilisation, raise the pH of the soil at a specific application rate, and increase the soil's water retention capacity, particularly in sandy and clayey soils (Antonangelo et al. 2021; Agegnehu et al. 2016a). As a result, recent research has examined the potential uses of co-composted biochar to accelerate the composting process and the formation of a stabilised end product to maximise carbon sequestration and soil fertility potentials (Antonangelo et al. 2021; Sánchez-García et al. 2015; Zhang and Sun 2014).

#### Influence of co-composted biochar on soils, plant growth and yields

Table 13 summarises the major effects of co-composted biochar on agricultural crops and soil characteristics. It can be clearly noted that co-composting biochar with substrates improves the soils' physicochemical properties. Meanwhile, it enhances crop yields and improves the overall health status. Thus, co-composting with biochar not only reduces gaseous emissions during the composting process, but also increases crop productivity.

**Table 13:** Impact of co-composted biochar on soil characteristics and agricultural crop productivity

Biochar substrate	Crop used	Impacts on soil	Impacts on plant	References
Green waste	Apple	Not mentioned	Improved stem thickness; no impact on yield	Eyles et al. (2015)
Bamboo	Corn	Improved soil available phosphorus and total nitrogen	Enhanced plant growth and production	Doan et al. (2015)
Grape pomace and rice husk	Ryegrass	Enhanced soil nutrient retaining capacity	Improved dry matter production by up to 78%	Manolikaki and Diamadopoulos (2017)
Palm fruit bunch	Cucumber	Improved soil cation-exchange capacity, carbon, nitrogen and water holding capacity	Enhanced plant biomass and fruit production	Cao et al. (2018)
Waste willow wood (*Salix spp.*)	Corn (*Zea mays*)	Increased dissolved organic carbon, available phosphorus, calcium and cation-exchange capacity & improved soil water retention and lowered nitrous oxide emissions over time	Increased yield; increased nutrient uptake by plants	Agegnehu et al. (2016a)
Eupatorium adenophorum	Corn (*Zea mays*)	Improved soil cation-exchange capacity and calcium, potassium, and magnesium	Yield increased by 243%	Pandit et al. (2019)
Acacia (*Acacia spp.*)	Barley (*Hordeum vulgare*)	Improved soil moisture retention and increased dissolved organic carbon by 34% and cation-exchange capacity by 24%, and improved soil fertility	Yield increased	Agegnehu et al. (2016b)
Wheat straw	Corn	Improved soil organic carbon, nitrogen, phosphorus and potassium content, reduced pH	Increased leaf chlorophyll content	Naeem et al. (2018)
Hardwood	Apple	Improved soil organic carbon and bioavailable phosphorus quantity by up to 300%	Enhanced shoot number and a trunk diameter of apple trees by up to 23–26%	Safaei Khorram et al. (2019)
Oak	Grape (Viti) and greenhouse tomato (*Lycopersicon esculentum*)	Increased soil pH, nutrient contents, dissolved organic carbon, and improved biological activity of the soil	Enhanced both fruits quality and crops yield	Sánchez-Monedero et al. (2019)
Willow	Peanut	Enhanced soil organic carbon, soil water content and cation-exchange capacity	Improved seed production and total pod production by 22–23%, respectively	Agegnehu et al. (2015)
Willow	Papaya and banana	Improved soil organic carbon, soil water content, cation-exchange capacity, potassium and calcium content	No impact on Papaya yield; reduced banana production by 18%	Bass et al. (2016)
Garden peat	Wheat	Improved phosphorus, potassium, and total nitrogen value of soil	Enhanced grain production by 27%	Qayyum et al. (2017)
Oak residue	Grape	Improved carbon sequestration and soil available nutrients	Improved yield by 50%	Oldfield et al. (2018)
Wood	Quinoa	Enhanced soil water retention capacity	Improved biomass production by 68%	Kammann et al. (2015)

Various biochar substrates were studied along with the crops used. General improvements in soil and crop productivity are observed

Naeem et al. (2018) demonstrated that co-composted biochar application decreased the pH of the soil and indicated that it could improve corn yields on alkaline soils. However, Major et al. (2010) reported that co-compost supplements could raise the soil pH and increase the retention of basic cations in acidic soils, thereby improving crop nutrition. As a result, acidic soils benefit from alkaline biochar; however, slightly alkaline biochar can still be used in alkaline soils (Naeem et al. 2018; Major et al. 2010).

Biochar contains essential macro- and micronutrients, including nitrogen, potassium, phosphorus, and calcium, which crops can utilise (Manolikaki and Diamadopoulos 2017). Co-composted biochar improves soil nutrient conditions by increasing the nitrogen, potassium, phosphorus, and calcium values as well as the cation-exchange capacity of the soil (Cao et al. 2018; Qayyum et al. 2017). Thus, soils retain a greater amount of nutrients and cations for subsequent plant uptake (Agegnehu et al. 2017). According to Hong and Lu, co-composted biochar can increase the bioavailability of phosphorus in soils and reduce the need for additional phosphorus fertilisers in supplemented soils (Hong and Lu 2018).

The application of co-composed biochar to soils can increase the organic carbon content of the soil as well as soil moisture. Agegnehu et al. (2015) observed that when biochar was added to fertiliser-amended soils, the carbon content increased from 0.93 to 1.25%, while the moisture content increased from 18 to 23%. Similar findings of increased soil moisture content in soils supplemented with co-composted biochar have been published (Naeem et al. 2018). Moreover, biochar can enhance the porosity and physical quality of soils, which can stimulate aggregation and restructuring of the soil's porosity arrangement, thereby affecting the soil's water retention capacity (Sun and Lu 2014). Several other reports support this conclusion (Guo et al. 2020a; Agegnehu et al. 2015; Bass et al. 2016; Liang et al. 2014).

However, not all studies demonstrated beneficial effects of biochar-amended soils on crop yield or growth. Borchard et al. (2014) discovered no effect of biochar addition on corn yield, either positive or negative. Xu et al. (2016) observed a decrease in *Suaeda salsa* biomass following biochar application compared to the non-applied crop, as well as a slight enhancement of sodic saline soils following biochar amendment. Bass et al. (2016) observed an 18% decline in banana yield following biochar additions, but no effect on papaya production. Other researchers concluded that increased plant growth or yield is primarily due to inorganic fertiliser application rather than biochar addition, and thus that adding biochar to soils may have had no or a negative effect on plant growth or yield (Kamau et al. 2019; Sorrenti et al. 2019).

According to Table 13, co-composted biochar has a significant potential to advance crop production; however, some reports indicate that it has a negligible effect on crop yield. Generally, prior to initiating the aerobic composting operation, mixing biochar with compost substrates that are high in nutrients and low in organic carbon increases the agronomic value of biochar-amended soil. Thus, biochar co-composting may overcome biochar's low nutrient value, adjust nutrient delivery from the compost, and reduce contaminant and nutrient leaching, resulting in a promising soil amendment concept. However, adding biochar to agricultural crops alone has not resulted in increased crop yields (Hagemann et al. 2017b; Graber et al. 2010). Therefore, as previously discussed, the application methods for biochar in soils are critical in determining its potential impact.

To summarise, adding biochar to composted substrates after the composting process has been completed and prior to soil application may improve soil properties and plant growth (Cao et al. 2018; Naeem et al. 2018; Safaei Khorram et al. 2019). However, adding biochar at the start of the composting process (co-composting) has various advantages. Głąb et al. (2018) investigated the enhanced water holding capacity of sandy soils amended with co-composted biochar. Kammann et al. (2015) demonstrated increased nitrate retention, which prevents it from leaching out of soils following the application of co-composted biochar, due to its nano/microporosity and ion water holding capacity. Additionally, it was reported that co-composted biochar had a greater effect on crop yields and soil quality (Hagemann et al. 2018).

Co-composting with biochar can enhance the interactions between biochar and compostable substrates, thereby increasing the function of both materials (Wu et al. 2016). Moreover, this combination accelerates the formation of phenolic and carboxylic groups on the surface of biochar, thereby increasing its reactivity (Wiedner et al. 2015). Furthermore, the addition of biochar aids in the humification of the mixture and improves the quality of the compost. After reacting with biochar during the humification stage, heavy metals in compost piles are immobilised (Guo et al. 2020a). As a result, co-composted biochar added to soils accomplishes two goals: (i) it effectively stabilises organic matter, and (ii) it increases the agronomic value of the composted material, implying that coupling biochar and compost may serve a similar function to chemical fertilisers.

#### Mitigation of salinity and drought stresses

The primary stressors affecting global crop yields are salinity and drought (Kanwal et al. 2017), whereby their presence can significantly impair nutrient uptake, growth, and plant production. Drought, in particular, can deplete crop chlorophyll levels and gas exchange capacity by inducing oxidative stress (Abbas et al. 2018a). Several studies have demonstrated the beneficial effects of co-composting with biochar on soils in terms of alleviating salinity and drought stress and increasing crop yields (Lashari et al. 2013; Artiola et al. 2012), where biochar addition improves the water-holding capacity of soils, the amount of water available to the soil, and the biological and physical characteristics of soils during drought (Artiola et al. 2012; Maienza et al. 2017).

Similarly, Jačka et al. (2018) demonstrated that the surface area of biochar (hydrogen bonds) could efficiently interact with water molecules and increase the water retention capacity of soils by 5%. Furthermore, Burrell et al. (2016) reported that co-composted straw biochar improved soil aggregation stabilisation, which increased available water to plants. Moreover, biochar addition could affect the physiological and morphological characteristics of crops grown in stressed soils. Abbas et al. (2018a) indicated an improvement in photosynthetic rate, transpiration rate, stomatal conductance, chlorophyll content, and water utilisation rate in drought-stressed soils following application of rice straw-derived biochar, as well as an increase in the wheat crop's antioxidant enzymatic activities. Similar findings revealed that adding 3% biochar increased the available water content, biomass density, and photosynthesis of plants exposed to drought by 24, 35 and 39%, respectively (Hashem et al. 2019). On the other hand, some reports indicated a slight increase in the water-holding capacity of drought-stressed soil amended with biochar (Keshavarz Afshar et al. 2016; Tanure et al. 2019).

The regulation of enzymes, microbes, and phytohormones induced by biochar has the potential to alleviate salinity-stressed soils. Lu et al. (2015) revealed that incorporating manure co-composted with biochar increased the activity of phosphatase and urease in saline-stressed soils, thereby mitigating the effect of salinity on crops. Similarly, it was observed that plants grown in saline soil amended with biochar had lower sodium levels, polyamines/polyamine oxidase activity, as well as jasmonic and abscisic acid levels (Farhangi-Abriz and Torabian 2017). Additionally, biochar may enhance the salinity effect via reduction and sorption of sodium uptake by crops (Ali et al. 2017). Overall, the addition of biochar can improve the crops' salt tolerance ability in salinity-stressed soils by decreasing mineral accumulation, sodium uptake, as well as phytohormone and stomatal conductance regulation (Ali et al. 2017).

#### Interactions with heavy metals in soils and plants

Co-composting with biochar has been shown to have a significant effect on the speciation and bioavailability of heavy metals, as well as the ability to immobilise metals in amended soils (Ouyang et al. 2017). Furthermore, it has been shown to reduce cadmium discharge from the soil into nearby bodies of water (Ouyang et al. 2017). Similarly, co-composting with biochar derived from woody biomass decreased zinc, lead, and cadmium concentrations in amended soils by 92, 86, and 5%, respectively (Karer et al. 2018).

The following mechanisms can be attributed to the stabilisation of heavy metals in biochar-amended soils: (i) the high specific surface area and extensive porosity of biochar enhance metal adsorption capabilities (Ouyang et al. 2017); and (ii) biochar's sorption capacity is linearly correlated with the presence of oxygen-containing functional groups on its surfaces(Zhang et al. 2014d). Functional groups on the surface of biochar, such as hydroxyl, carboxyl, and phenolic, are critical for heavy metal retention when co-composted biochar is applied to soils (Uchimiya et al. 2011). However, the ability of biochar to remove heavy metals is entirely dependent on the type of heavy metal and the soil characteristics. According to Kloss et al., biochar application increased the amount of molybdenum, while decreasing the amount of copper, manganese, and cadmium in plants (Kloss et al. 2014). Uchimiya et al. reported that biochar could help stabilise heavy metals [such as copper(II) and lead(II)] in acidic, low organic carbon, and low cation-exchange capacity soils (Uchimiya et al. 2011).

Biochar has an alkaline pH, which is typically higher than that of soils; thus, biochar has the potential to immobilise metals, particularly in soils with a low pH (Puga et al. 2015). Kloss et al. discovered that adding biochar to soil can significantly increase arsenic levels, which could contaminate subsequent groundwater in areas with heavy rainfall (Kloss et al. 2014). Similarly, Beiyuan et al. observed an increase in arsenic mobilities and bioavailability in soil amended with biochar, which they attribute to the pH change following biochar amendment (Beiyuan et al. 2017). Additionally, biochar-amended soil has the potential to alter the rate of heavy metal uptake by plants. For example, Zhou et al. observed a decrease in the exchangeable rate of heavy metals from soils to plants, which resulted in less metal build-up in plants (Zhou et al. 2018a). Similarly, in soils supplemented with biochar, heavy metals migration to above-ground plant parts was reduced (Zhu et al. 2015). It was noted that mine soils amended with biochar decreased the bioavailability of zinc, lead, and cadmium by 54, 50, and 56%, respectively, reducing the plants' uptake of heavy metals (Puga et al. 2015).

### Summary

This section thoroughly investigated the impact of utilising biochar within the composting process, demonstrating clear agronomic and climate mitigation related benefits. Our analysis confirms that the composting process, along with the subsequent long-term storage in soils, is a robust biochar-based carbon sink application. However, it is recommended to address the following points moving forward.

- Several studies have established the beneficial effects of biochar-amended soils on crop health and yields; however, this effect is highly variable depending on soil type, biochar application method, and plant type. As a result, the specific mechanisms by which biochar exerts its various effects require further investigation.
- The subsequent immobilisation of heavy metals in soils is a critical constraint on expanding the use of co-composted biochar in soils, resulting in metals deficiency in plants. Thus, optimising the degree of immobilisation of heavy metals in order to avoid heavy metal deficiency in plants is an important area of future research.
- Long-term field applications of co-composted biochar in soils are necessary to assess the potential effect on soils, plants, and long-term strategies for contaminants mitigation. Additionally, the periodic application of co-composted biochar requires additional research.
- While plant-derived biochar received considerable attention, there are still constraints on utilising other high-value feedstocks, such as seaweeds and fish shells.
- Additional research is needed to compare co-composted biochar to other organic and inorganic fertilisers in terms of efficiency, disadvantages, and environmental impact.
- Continuous amending of co-composted biochar can result in excess of nutrients being added to the soil, particularly nitrogen and phosphorus, resulting in water pollution (groundwater eutrophication). As a result, optimising the application rate concerning the plant's consumption rate is necessary and will continue to be a focus of future research.
- Life cycle assessments of the greenhouse gas potential of soil amended with co-composted biochar are urgently needed to determine the degree of environmental impact associated with the application of such co-compost in comparison with standard compost without the addition of biochar and inorganic fertilisers.

## Environmental remediation

**Fig. 6:**
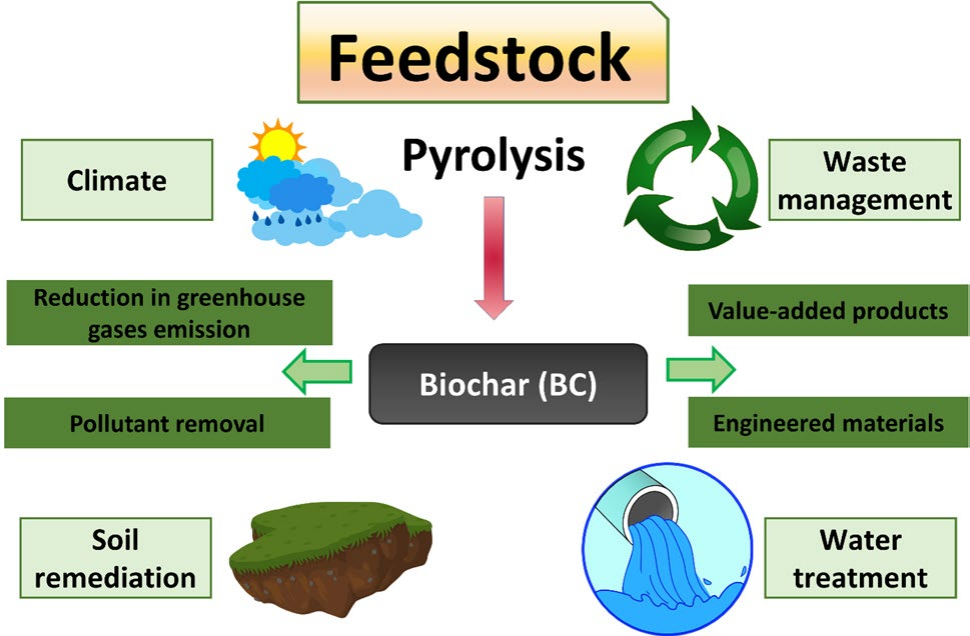
Applications of biochar in the environmental remediation contexts. Pristine and engineered biochar could be employed in a variety of applications, including water treatment, soil remediation, and carbon sequestration, hence mitigating climate change. Converting biogenic residues to biochar enables further value development from a waste management perspective

The global community is currently facing unprecedented environmental concerns. Contaminants emitted from residential, commercial, and industrial sources frequently affect the environment (Monisha et al. 2021). The literature demonstrates that water and soil are more vulnerable to both organic and inorganic contaminants mainly caused by human activity. However, technological developments in water and soil remediation have accelerated recently. In this context, the utilisation of biochar in environmental remediation applications has emerged as a highly promising technique. This approach facilitates value maximisation, in which biochar can carry out a remediation role in conjunction with its carbon sequestration purpose. This section will further investigate the dimensions related to the use of biochar in water treatment and soil remediation applications. Figure 6 illustrates the various aspects in which value is created in this context.

### Water remediation

Global concern about water pollution and the associated challenges related to the production and disposal of massive amounts of industrial effluents as well as stormwater has prompted the scientific community to explore efficacious and cost-effective solutions (Arslanoğlu et al. 2020). As a result, biochar has been promoted as a viable option for treating water contaminated with various emergent pollutants (Kamali et al. 2017). In general, the technical effectiveness of biochar-assisted water treatment pathways is heavily influenced by operational parameters such as ease of application, treatment efficacy, process robustness, scalability, and suitability for integration with other water treatment strategies (Kamali et al. 2021). Indeed, the sorption potential of biochar is attributed to its unique physicochemical properties, including specific surface area, ion exchange capacity, microporosity, and loading capacity. The unique characteristics of the as-prepared biochar samples dictate how various contaminants interact with it via various mechanisms (Qiu et al. 2021; Zhang et al. 2020c). The following section discusses the current state, challenges, sustainability concerns, and knowledge gap associated with biochar’s potential in the remediation of contaminated water.

#### Synthesis of biochar and the related physicochemical attributes

It is generally understood that the surface chemistry (i.e. charge, functional groups, elemental composition, and acidity/basicity), as well as the morphological characteristics (specific surface area, and pore distribution) of the biochar, are all important factors influencing its adsorptive capacity (Xiang et al. 2019; Akhil et al. 2021). Nature of the feedstock, pyrolysis operational parameters (i.e. pyrolysis temperature, heating rate, residence time, and carrier gas type/flow), and surface functionalisation/activation play a paramount role in imparting the biochar its adsorptive features (Ersan et al. 2017; Krasucka et al. 2021). Exploring the relationships between these variables and biochar features is of interest for synthesising a ‘model’ biochar with optimised physicochemical properties for water remediation. Therefore, this section will thoroughly discuss the relationship between biochar synthesis conditions and their physicochemical properties.

Thermochemical treatment is conventionally used to convert biomass into biochar. The conversion scheme varies depending on the treatment temperature, residence time, heating rate, and the reaction atmosphere. The composition of the biochar is mainly dependent on the parent raw materials (i.e. agricultural residues and forestry wastes) utilised in its production (Fan et al. 2017). In other words, the percentage of cellulose, hemicellulose, and lignin in different biomasses will determine the elemental composition of the biochar. Mostly, the larger the lignin content in biomass, the greater the fixed carbon content in the resultant biochar (Vyavahare et al. 2019). Increased cellulose and hemicellulose concentrations in the biomass result in an additional microporous structure, whereas increased lignin content results in significant mesoporous structures with a large surface area and increased aromaticity (Solanki and Boyer 2017).

In general, biochar is composed of carbon, hydrogen, oxygen, nitrogen, and traces of elements such as alkali and alkaline earth metals, with carbon accounting for more than 50% of the total weight% (excluding manure and sludge-based biochar). Both alkali and alkaline earth metals could directly influence the cation and anion exchange loading capacities as well as the pH value of the prepared biochar (Boakye et al. 2019). Furthermore, most biochar produced from agricultural or forest residues has high carbon content in comparison with the other types of biochar, which results in a higher yield as well as carbon- and oxygen-containing functionalities that serve as additional effective sites for capturing target pollutants (Huang et al. 2019c). Similarly, the biochar produced from industrial and municipal solid wastes typically has a very high content of ash and non-organic components, improving the biochar loading capacity for phosphate (Takaya et al. 2016). The literature shows that the excessive mineral content in the biochar, which could occupy the available binding sites and hence, deteriorates the biochar porous network structure, affects the overall surface activity adversely.

The pyrolysis temperature has a significant effect on the properties of the resulting biochar. The aromaticity, carbon content, microporosity, surface area, and hydrophobicity increase as the operating temperature increases. Similarly, as the pyrolysis temperature increases, the pH of the biochar increases due to the increased ash content. Low temperature–biochar (< 500 °C) exhibits low polarity, a low carbon/nitrogen ratio, and a higher concentration of dissolved organic carbon in addition to functional moieties containing oxygen. In contrast, high-temperature–biochar (> 500 °C) possesses low acidity, caused by loss of oxygen and hydrogen-containing functionalities (Pokharel et al. 2020a). It is believed that negatively charged oxygen-containing groups such as alcohols, carbonyl, and carboxylic acid enhance the biochar's cation-exchange capacity towards cationic pollutants. Similarly, it is believed that the oxonium functional group enhances the biochar's anion exchange capacity (Pokharel et al. 2020a; Janu et al. 2021).

#### Parameters affecting the adsorption process in static and dynamic water treatment systems

Numerous studies, particularly at the laboratory scale, have examined the adsorption pathway using synthetic pollutant solutions (Xu et al. 2021; Yao et al. 2021; Yu et al. 2019a; El-Shafie et al. 2021; Hassan et al. 2020). However, biochar can be considered as a viable solution if employed at a large industrial scale (continuous systems) for water treatment. In comparison with synthetic solutions, wastewater is discharged into aquatic systems in large quantities and with complex characteristics, which can affect the biochar's adsorptive performance (Reguyal and Sarmah 2018). In laboratories, the batch (static) mode is frequently used to determine the adsorption kinetics, isotherms, and thermodynamics of the adsorbents in use. However, due to the large volumes of released effluents that must be treated, this approach is rarely used in industrial sectors (Mazur et al. 2018).

Typically, dynamic, fixed bed (continuous flow) systems are recommended in this case because they closely resemble large-scale industrial applications (de Franco et al. 2017). Despite the widespread use of fixed-bed columns, packing the bed with pristine biochar may be impractical due to its low mechanical strength, low density, and poor hydraulic conductivity (Jung et al. 2017). To overcome these constraints, biochar particles encapsulated in a polymeric matrix, as well as other strategies for physicochemical modification, can be used (Panahi et al. 2020). Moreover, the presence of other competitive species may significantly impair the biochar's adsorptive capacity (e.g. inorganic and organic pollutants). Thus, understanding the effect of various operational parameters on adsorption processes in batch or fixed-bed modes is considered fundamental for a thorough understanding of the entire process (Baltrėnaitė-Gedienė et al. 2020; Shin et al. 2021).

##### Operating mode

Adsorption procedures are defined as the capacity of biochar (a solid adsorbent) to concentrate a specific adsorbate on its surface via an adsorbent-adsorbate interface in order to accomplish the desired separation or purification objective. Batch adsorption is the simplest method for promoting solid–liquid interaction. In this approach, a fixed amount of biochar is mixed with a known volume and concentration of pollutant solution in a sealed vessel. The system is agitated to ensure homogeneity of the solution, the initiation of solid–liquid interaction, and the acceleration of mass transfer until the saturation plateau is reached (Patel 2021). The performance of the biochar is primarily determined through the use of mathematical models of adsorption kinetics, isotherms, and thermodynamics.

Fixed-bed columns are frequently used in continuous-flow systems. The operation of this unit begins with the feeding of a pollutant solution into a column packed with a fixed amount of biochar. Adsorption equilibrium is reached when the adsorbate concentrations at the inlet and outlet are equal. Typically, fixed bed technology operates as a function of bed height and adsorbate flow rate, which are used to construct breakthrough curves for subsequent analysis. While evaluating the purification process via column studies is more practical than batch studies, this does not negate the importance of batch studies (Thirunavukkarasu et al. 2021).

Nascimento et al. investigated the adsorption of two different types of cationic (basic blue 12) and anionic (reactive black 5) dyes onto wood residue-based biochar in batch and fixed-bed operation modes. They reported that the maximum loading capacities of as-used biochar were 35.67 and 80.41 mg g^−1^ for reactive black 5 and basic blue 12, respectively. Additionally, the breakthrough curve studies determined that 100 and 224 L of reactive black 5 and basic blue 12 can be purified using the fixed-bed pilot plant, respectively (do Nascimento et al. 2021).

##### Primary pH of the target pollutant

The pH of the contaminant solution is a critical parameter in the adsorption process because it has a significant effect on the adsorbent surface charge, solution chemistry, and degree of ionisation of the adsorbate, as well as the competitive adsorption between hydron/hydroxide and target pollutants (Al-Saad et al. 2019; Xiang et al. 2020a). Numerous studies have established that the protonation and de-protonation of adsorbents containing functional groups in acidic and alkaline environments, respectively, are maintained by the effect of initial solution pH. Katiyar et al. concluded that raising the pH of the solution from 1 to 5 caused the surface of the biochar to become more negatively charged, favouring copper adsorption with almost 100% removal via electrostatic interactions. At a pH range of 4–5, the maximum loading capacity of *Ascophyllum nodosum*-based biochar was achieved. Beyond a pH of 5, a small reduction in copper adsorption is noted, which may be attributed to the formation of hydroxo copper(I) hydroxide, which impedes the interaction of biochar and copper ions (Katiyar et al. 2021). Additionally, changes in pH can significantly affect the electrostatic interactions as measured by the zeta potential (point of zero charge) of the biochar. When the solution pH is less than the point of zero charge, this implies that the biochar surface is positively charged, which enhances the adsorption of negatively charged pollutants, and vice versa when the solution pH is greater than the point of zero charge (El-Azazy et al. 2020; El-Azazy et al. 2021a; Kelm et al. 2019).

##### Biochar dosage

The concentration of biochar has a substantial effect on the adsorption process. Determining the optimal dosage, above which further increases in biochar have no noticeable effect, is critical. Fundamentally, increasing the amount of biochar implies an increase in pollutant removal efficacy as the accessible surface area with more adsorptive sites increases (Fan et al. 2017). Meanwhile, due to the unsaturation state of biochar's unoccupied adsorptive sites, any increase in a concentration above the optimised value does not affect the adsorption process. This could be explained by the screen effect phenomenon, which occurs when biochar particles aggregate at higher concentrations (Wang et al. 2017d; Güzel et al. 2017).

##### Biochar particle size

Another critical parameter affecting the adsorption and ion exchange pathways is the particle size of the biochar. As the particle size decreases, the biochar surface area increases, shortening the adsorbate's diffusion path and ultimately increasing the adsorption capacity. Typically, the use of biochar powder is impractical due to operational disadvantages such as difficulty extracting from aqueous solutions, low mechanical strength, low density, poor hydraulic conductivity, and high flow resistance in a continuous-flow system (Quesada et al. 2020). Kang et al. investigated the effect of biochar particle size on phenanthrene adsorption. The equilibrium times decreased from 4.6–17.9 days (original biochar) to 1–4.6 days (powdered biochar) for the different particle sizes of 4, 2, and 1 mm and 500, 250, 150, and 75 μm (biochar without grinding), 200, 150, and 75 μm (biochar passed through 250 μm-size sieves), and 106, 75, and 63 μm (biochar passed through 125 μm-size sieves). These findings demonstrate the critical role of biochar particle size in optimising organic compound adsorption (Kang et al. 2018).

##### Interaction time

Interaction time is undeniably one of the most influential variables included in adsorption investigations. During the initial stages of adsorption, the abundance of void adsorptive sites significantly increases the adsorption rate. Following that, as the adsorbate gradually occupies the binding sites, the adsorption rate decreases until an equilibrium state is reached due to the gradual saturation of the adsorbent active sites. Theoretically, several kinetic models based on distinct theories are used to specify the equilibrium time, mass transfer rate, and adsorption governing stage. These models include pseudo-first-order, pseudo-second-order, intraparticle diffusion, Boyd, Bingham, and Elovich (Abdel Maksoud et al. 2020). The effect of interaction time on ciprofloxacin and sparfloxacin adsorption onto iron (II, III) oxide/graphene-modified biochar was systematically investigated. The authors reported that the maximum loading capacities of modified biochar for ciprofloxacin and sparfloxacin were 283.44 mg g^−1^ (equilibrium time of 36 h) and 502.37 mg g^−1^ (equilibrium time of 24 h), respectively (Zhou et al. 2019a).

##### Adsorption temperature

Temperature can have an effect on the adsorbent-adsorbate system, depending on the biochar/adsorbate characteristics and the exothermic or endothermic nature of the reaction. For example, the temperature can affect both the adsorption rate and the loading capacities of biochar. In the case of exothermic reactions, increasing the temperature of the reaction may inhibit adsorption by weakening the physical bond between the adsorptive sites on the biochar surface and the adsorbate (Ahmed et al. 2017). Cai et al. (2019) demonstrated that as the temperature increased, the solubility of oil increased, resulting in increased interactions between oil molecules and the solvent, thereby inhibiting oil molecule adsorption onto the biochar. Contrarily, in the case of an endothermic reaction, an increase in the temperature may promote adsorbate migration onto the surface of the biochar. Several studies corroborated this assertion (Wu et al. 2018; Zhou et al. 2019b). Additionally, elevated temperatures may strengthen the electron donor and acceptor, and hence, improve the π–π electron donor–acceptor interactions between organic pollutants and biochar (Ai et al. 2019).

##### Primary pollutant's concentration

The influence of primary concentration is highly relevant to the adsorption of aquatic pollutants. As pollutant concentrations increase, the biochar's loading capacity increases proportionately until the biochar becomes completely saturated. This is due to an increase in the concentration gradient between aquatic pollutants (molecules or ions) and specific sorptive sites on the biochar surface, which drives the adsorption process up to saturation. In contrast, no further upgrading occurs (Elgarahy et al. 2019). Numerous isotherm models based on the researched parameters; two (e.g. Langmuir, Freundlich, Temkin, Dubinin–Radushkevich), three (e.g. Hill, Toth, and Slip), and four (e.g. Baudu and Weber–van Vliet) were used to attempt a systematic understanding of the adsorption isotherms. Li et al. (2020d) demonstrated that the loading capacities of lanthanum-coated biochar-based urban dewatered sewage sludge were closely related to the primary phosphate concentrations in order to overcome the strict mass transfer resistance.

##### System complexity

The complexity of the system caused by the coexistence of inorganic, organic, and ionic species can significantly affect the adsorption process. As a result, understanding the synergism or antagonistic effects of these interferents is critical for optimising the efficacy of the water treatment process. The presence of these competitive species may result in the formation of complexes, lowering the biochar's affinity for target pollutants. On the contrary, these species may enhance the performance of the adsorption process via certain intermolecular forces, such as co-adsorption and sating-out phenomena (Ahmed and Hameed 2018). Choudhary et al. investigated the competitive adsorption of copper and nickel metal ions and malachite green dye molecules and reported that the loading capacities of *Opuntia ficus*-indica-based biochar for copper decreased from 13.76 to 10.65 and 10.24 mg g^−1^, respectively, in binary and ternary systems. Similarly, the loading capacities towards nickel declined from 11.36 to 5.59 and 4.49 mg g^−1^ in binary and ternary systems, respectively (Choudhary et al. 2020).

##### Flow rate of pollutant feeding solution

In a fixed-bed system, the inlet flow rate significantly affects the profile of the breakthrough curve due to variations in the feed superficial velocity and interaction time. Reduced flow rates maximise the interaction time between the feed solution and the packed biochar, ensuring a sufficient residence time for adsorption (Thirunavukkarasu et al. 2021). Jung et al. investigated the adsorption performance of electrochemically modified biochar calcium-alginate beads towards phosphate at flow rates of 2.5, 5.0, and 7.5 mL min^−1^, with a fixed amount of biochar and a fixed concentration of primary phosphate. They demonstrated that increasing the inflow rates significantly decreased breakthrough and saturation times from 168.3 to 32.9 min and 308.0 to 322.5 min, respectively. This can be explained by the phosphate ions' insufficient residence time on the modified biochar surface to achieve equilibrium (Jung et al. 2017).

Moreover, there is a clear relationship between the superficial velocity of the liquid, the flow rate, and the mass transfer resistance. Evidently, the greater the liquid's superficial velocity, the less resistance there is to mass transfer. However, if the adsorption rate is determined by intraparticle diffusion, the liquid superficial velocity is unlikely to have a significant effect. Accordingly, feeding flow rate control is a critical operational parameter for achieving adequate residence time, avoiding adsorbent channelling, and minimising liquid-film resistance (Fernández-González et al. 2019).

##### Biochar bed height

Another critical parameter affecting the fixed-bed adsorption system is the height of the biochar. Considering the pressure drop across the working system, utilising biochar with the appropriate mechanical strength and particle size is highly recommended to improve adsorption performance. In practice, raising the bed height tends to increase the number of potential adsorptive sites, the time required for adsorbate to interact with biochar, and, as a result, the total amount of adsorbate captured (Abdel Maksoud et al. 2020). It is important to note that increasing the bed height reduces axial dispersion and eliminates the challenges associated with fluid maldistribution throughout the bed's length. By maintaining a ratio of 5 between bed height and column diameter, liquid maldistribution can be avoided, while axial dispersion can be kept to a minimum by maintaining a ratio of larger than 150 between bed height and adsorbent particle diameter (Inglezakis and Poulopoulos 2006). With increased bed heights of packed adsorbent between 2, 3 and 4 cm under constant flow rate (4 mL min^−1^) and chromium concentration (100 mg L^−1^), the feeding solution widened the mass transfer zone, prolonged breakthrough and saturation times from 300 to 1260 min and 2640 to 4740 min, respectively, and thus enhanced the adsorptive performance of biochar (Nithya et al. 2020).

##### The concentration of pollutant feeding solution

As is the case with batch (static) adsorption systems, the input concentration of the pollutant feeding solution is a critical process parameter that affects both mass transfer and treatment process performance. During the biochar-adsorbate interaction, the larger concentration gradient, which is associated with a higher concentration of pollutant feeding solution, acts as a driving force for mass transfer during the adsorption process. However, the residence time is insufficient to achieve the integral adsorption when the primary pollutant concentration is excessively high (Banerjee et al. 2018). Vidhya et al. (2020) investigated the effect of various nickel ion concentrations on the adsorption of nickel onto coir pith-based biochar. With increasing inflow pollutant concentrations, a decrease in the length of the adsorption zone, a steeper breakthrough curve, and an increase in the diffusion coefficient were observed. This is explained by the progressive concentration gradient between the modified biochar and nickel ions, which accelerates both biochar saturation and the breakthrough curve.

#### Biochar optimisation for water treatment processes

As demonstrated in the preceding section, numerous and interacting variables influence the performance of biochar in water remediation. Furthermore, it can be concluded that the adsorption process is a complicated one that cannot be viewed from a single perspective. Therefore, a comprehensive overview is required to balance the need to meet process objectives (maximum biochar yield, maximum adsorption capacity, and maximum percent removal) with the need to maintain the process sustainability by minimising chemical consumption, workforce effort, time, and resources. As a result, one of the primary challenges inherent in this process is the appropriate selection and optimisation of process variables in order to maximise biochar yield and performance. Therefore, the operation of predictive models serves to reduce the burden on the environment, improve method greenness, and contribute to the long-term goals of sustainability and circular bioeconomy (El-Azazy et al. 2020; El-Azazy et al. 2021a; Bhagat et al. 2020; Hiranjit 2015; Hodgson et al. 2016; Karimifard and Alavi Moghaddam 2018; Kostić et al. 2016; Lakshmi et al. 2021; Puccini et al. 2017).

Using a black-box model to design a process and optimise a response statistically was once common in the engineering field. Until now, considering such an approach to chemistry, environmental engineering, and the production and application of biochar in water treatment is still in infancy. Coupling environmental bioremediation techniques with artificial neural networks, design of experiments, response surface methodology, and multiple linear regression is currently trending, owing to the growing awareness of sustainability and green chemistry (Terayama et al. 2021). In general, coupling an analytical process to the competency and efficiency of these predictive models could be utilised constructively to better understand the complicated biochar-pollutant interactions. When fully developed, these models may enable the automation of biochar production and the commodification of biochar applications in water remediation.

Generally, the literature indicates that optimisation efforts are often focused on two distinct contexts: (i) biochar production and (ii) application in water treatment processes. Additionally, these efforts are contingent upon the intended application of the biochar and whether it will be used in a batch or continuous mode (Bhagat et al. 2020; Hodgson et al. 2016; Karimifard and Alavi Moghaddam 2018; Lakshmi et al. 2021). The optimisation of the pyrolysis process entails adjusting variables such as the pyrolysis temperature, solid residence time, vapour residence time, heating rate, and impregnation ratio in the case of chemically modified biochar (Puccini et al. 2017; Ameen Hezam Saeed et al. 2022; Batista and Gomes 2021; Te et al. 2021). Similarly, other efforts were focused on variables such as biochar particle size, the sample mass, and the gaseous atmosphere in which the biochar is prepared (Hodgson et al. 2016; Kwak et al. 2019; Manyà et al. 2018; Pradhan et al. 2020; Rojas-Mayorga et al. 2013; Saadat et al. 2018; Xu et al. 2019).

In batch mode operation, variables affecting the removal process such as pH, adsorbent dose, initial concentration of the contaminant, and contact time have also been optimised (El-Azazy et al. 2020; El-Azazy et al. 2021b; El-Azazy et al. 2021c; Bardestani et al. 2019; Beakou et al. 2017; Kalderis et al. 2017; Lenin Sundar et al. 2021; Roy et al. 2018; Turk Sekulic et al. 2018; Zhao et al. 2021d). Variables such as fixed-bed height, flow rate, the particle size of the biochar adsorbent, pH, and concentration of the inlet solution are optimised in continuous mode operation (Biswas et al. 2020; Blagojev et al. 2019; Levio-Raiman et al. 2021; Sivarajasekar et al. 2018; Suárez‐Vázquez et al. 2021). As a result, the target response (output) will vary according to the input variables assessed. The commonly optimised responses (output variables) are biochar yield, breakthrough time, contaminant removal rate, and adsorption capacity (Qe, mg g^−1^).

The following subsections will provide a concise overview of the most commonly used designs and black box models for optimising the use of biochar in water treatment strategies. This review excludes design theories, specifications, and their associated benefits and drawbacks (Karimifard and Alavi Moghaddam 2018; Lakshmi et al. 2021; Elazazy 2017).

##### Design of experiments and response surface methodology

The univariate analysis is the conventional method for determining the effect of various variables on a process. In this strategy, the effect of a single independent variable (input variable) on a single response (dependent variable) is simultaneously measured, while the remaining variables are held constant. Numerous researchers continue to use this method to produce biochar and its applications. Regardless of the cost, time, and effort involved in developing such a technique, the lack of a holistic view of the process and its variable-variable interactions presents a significant challenge. As a result, the motivation to use the design of experiments, also referred to as DoE, emerged (Karimifard and Alavi Moghaddam 2018). Combining the design of experiments with the production of biochar or its application in the removal of contaminants results in not only a significant reduction in the number of experimental runs but also a better understanding of variable interactions and thus, the generation of data with a high degree of reliability. The resulting mathematical model is used to create a comprehensive picture of the process, indicating which variables significantly affect it, their magnitude, and their interaction with others (Elazazy 2017).

The design of the experiment's approach is typically divided into two phases: screening and optimisation. The first phase mainly evaluates the process's input variables. The researcher typically proposes variable limits (upper and lower) based on preliminary experiments or prior knowledge. Numerous designs, including the definitive screening design, the full factorial design, the fractional factorial design, the Plackett–Burman design, and the Taguchi design, can be used for screening. The subsequent phase uses statistically significant variables in the first phase as input, with their limits finely adjusted. The most frequently used optimisation techniques are central composite design, Box–Behnken design, Doehlert design, and d-optimal design.

**Fig. 7:**
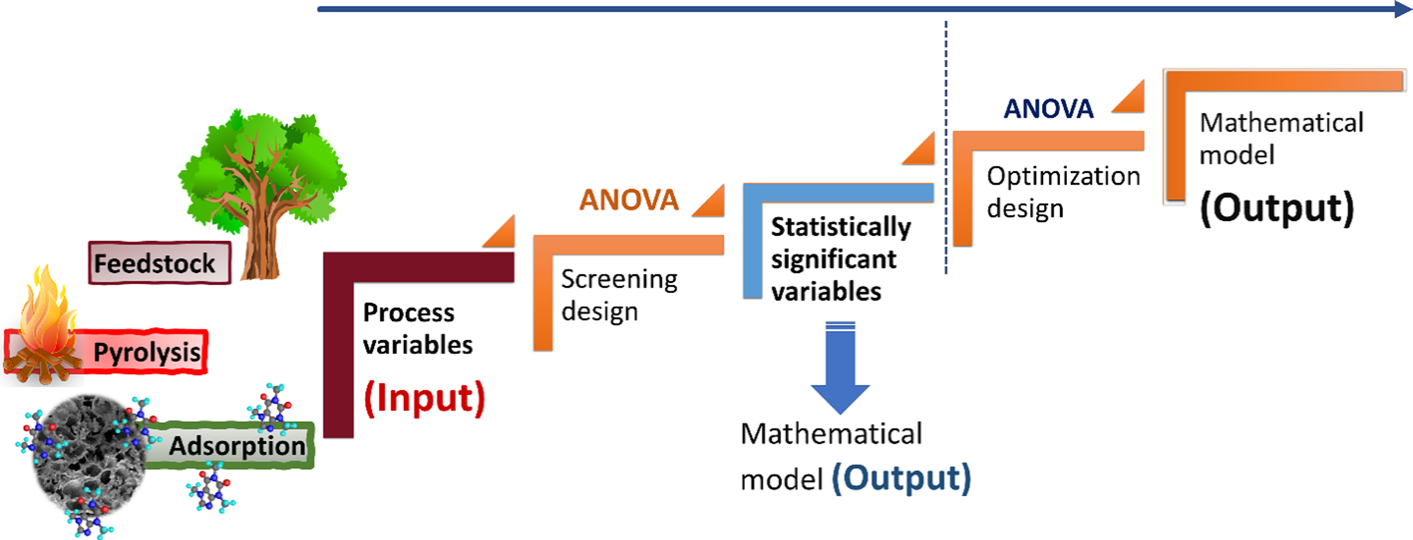
Phases of experiment design. The design of experiments begins with a screening of the input variables (related to feedstock, pyrolysis, or the adsorption process). The second stage is optimisation, which involves the use of statistically significant data to construct the mathematical model. Each phase is typically accompanied by statistical analysis using analysis of variance, also referred to as ANOVA

Response surface methodology is the approach that refers to the process of screening and/or optimisation followed by fitting the obtained mathematical models to the experimental data. The primary objective of response surface methodology is to determine the optimal operating requirements for the desired system or to obtain a zone that meets those requirements. Choosing one of these designs requires considering the experimental objective and the number of variables to be investigated. Each of these phases is accompanied by a set of quality indicators, including analysis of variance, probability testing, correlation coefficient, and coefficient of determination. Figure 7 illustrates the various phases of the design of experiments and the associated verification procedures.

The application of various design of experiments and response surface methodology approaches to optimise biochar production and utilisation for the removal of various organic and inorganic contaminants is shown in Table 14. Additionally, the variation in input/output variables based on the intended application is demonstrated. As shown in Table 14, response surface methodology-based optimisation approaches, Box–Behnken design, and central composite design were more frequently used in the literature. Furthermore, studies examining the contaminant adsorption process were more prevalent than those examining the effect of feedstock parameters and pyrolysis conditions on biochar yield and physicochemical properties of the resulting biochar. Additionally, a few studies have been conducted to optimise pollutant removal in the continuous mode of operation.

**Table 14:** Predictive models, independent and dependent variables, and performance indicators for applying biochar in removing different pollutants from water

Feedstock	Target pollutant(s)	Optimisation approach	Parameters optimised	Optimised response variable(s)	Design performance indicators	References
*Lolium perenne, Miscanthus x. giganteus, Fraxinus excelsior, Salix viminalis, Picea sitchensis*	Zinc (II)	Taguchi design—L9 orthogonal array	Temperature, residence time, particle size, and gaseous atmosphere	%Zinc adsorbed, Char yield and composition	Analysis of variance	Hodgson et al. (2016)
Sewage sludge feedstock	Phosphate	Central composite design	Pyrolysis temperature and time	% Removal of phosphate, and pH	Analysis of variance, Fisher test value (*F*-value), probability value (*P*-value), *R*^2^ and *R*^2^-adjusted	Saadat et al. (2018)
Cattail (*Typha latifolia*)	Phosphorous	Box–Behnken design	Lanthanum weight content in total biomass weight (feedstock parameters), pyrolysis temperature and maintenance time (pyrolysis parameters)	% Phosphate removal efficiency	*R*^2^ and Analysis of variance	Xu et al. (2019)
Shredded wood comprising 20% volume of spruce/fir and 80% pine (stem of *Pinus strobus* without bark)	Lead (II)	Central composite design	Acidity of aqueous solution (pH), agitation time, adsorbent mass, and initial concentration of lead (II)	Biochar extraction efficiency (*E* (%)) and adsorption capacity (*Q* (mg g^−1^))	*R*^2^ and *R*^2^-adjusted	Bardestani et al. (2019)
Paper sludge/wheat husks	2,4-dichlorophenol	Box–Behnken design	pH, temperature, initial contaminant concentration, and time	Adsorption%	*R*^2^ and *R*^2^-adjusted, Analysis of variance	Kalderis et al. (2017)
Tea waste	Fluoride	Central composite design	Adsorbent dose, contact time, and temperature	% Fluoride removal	*R*^2^, coefficient of variation, *F*-value, and *P*-value	Roy et al. (2018)
Cassava Rind	Malachite green	Box–Behnken design	Adsorption temperature, dose of absorbent, and initial dye concentration	Amount of malachite green adsorbed per unit mass of the adsorbent	*R*^2^, coefficient of variation, *F*-value, and *P*-value	Beakou et al. (2017)
*Prunus armeniaca* stones	Lead (II), cadmium (II), nickel (II), naproxen, chlorophenols		Adsorbent dosage, pH initial contaminant concentration	Yield%, percentage of adsorbate removal and equilibrium adsorption capacity, *q*_e_	The root mean square error and sum of the squares of the errors	Turk Sekulic et al. (2018)
Date pits	Tigecycline	Box–Behnken design	pH, adsorbent dose, initial drug concentration, and contact time	%Removal and adsorption capacity	*R*^2^ and *R*^2^-adjusted, *R*^2^ predicted, and analysis of variance	El-Azazy et al. (2021b)
Cow femur residues (bone char)	Fluoride	Fractional factorial design	Temperature, heating rate, and residence time	Yield%, and fluoride uptake (mg g^−1^)	Variance analysis	Rojas-Mayorga et al. (2013)
Pistachio nutshells and Aloe vera leaves	Sarafloxacin	Plackett–Burman design	pH, adsorbent dose, initial drug concentration, and contact time	%Removal and adsorption capacity	*R*^2^, *R*^2^-adjusted, *R*^2^-predicted, and analysis of variance	El-Azazy et al. (2020)
Olive stones	Clofazimine	Box–Behnken design	pH, adsorbent dose, initial drug concentration, and contact time	%Removal and adsorption capacity	*R*^2^ and *R*^2^-adjusted, *R*^2^-predicted, and analysis of variance	El-Azazy et al. (2021c)
Cow dung and sewage sludge	Methylene blue	Central composite design	Dose of biochar, initial methylene blue concentration, and the type of biochar	Breakthrough time	*R*^2^ and *R*^2^-adjusted, *R*^2^-predicted, and analysis of variance	Suárez‐Vázquez et al. (2021)
Soil, peat, and lignocellulosic material (straw)	Atrazine (pesticide)	Central composite design	The inlet atrazine concentration, flow rate and pH of the fixed-bed column packed with wheat straw, soil, and peat	% Removal and adsorption capacity	Analysis of variance, lack-of-fit	Levio-Raiman et al. (2021)
Sugar beet shreds	Copper (II)	Box–Behnken design	Concentration of the inlet solution, adsorbent dosage, and pH of the inlet solution	Breakthrough time	Analysis of variance and *R*^2^	Blagojev et al. (2019)
*Aegle marmelos Correa* fruits	Ranitidine HCl	Box–Behnken design	Fixed-bed height, initial drug concentration, flow rate, and adsorbent particle size	% Removal	*R*^2^, *R*^2^-adjusted, *R*^2^-predicted, analysis of variance, lack-of-fit	Sivarajasekar et al. (2018)
Sugarcane bagasse	Zinc (II), copper (II) and nickel (II)	Central composite design	Initial adsorbent bed height, initial solute concentration, liquid flow rate, and airflow rate	% Removal	*R*^2^, *R*^2^-adjusted, *R*^2^-predicted, analysis of variance, lack-of-fit	Biswas et al. (2020)
Seaweed *Ulva reticulata*	Reactive Red 120	Box–Behnken design	Biochar dose, initial dye concentration and pH	% Removal	*R*^2^, *R*^2^-adjusted, analysis of variance, lack-of-fit	Lenin Sundar et al. (2021)

This mainly includes the design of experiment-based approaches

##### Artificial neural networks

Artificial intelligence and machine learning are emergent statistical models that have been widely applied in various fields in recent years. These strategies are frequently based on the human brain's ability to process complex interactions between input and output variables. With the same objectives as the use of design of experiments in water bioremediation, the coupling of artificial neural network models to biochar production and utilisation is growing in popularity. However, with unprecedented advancements in data processing software and hardware, as well as the volume and quality of data generated, approaches based on artificial neural networks are more powerful. Indeed, using data generated by artificial neural networks-based models for comprehending input/output data, contaminant fate, and adsorption process is promising (Lakshmi et al. 2021; Medeiros et al. 2022).

As with design of experiments, the use of an artificial neural networks-based approach could be applied to any stage of the biochar's life cycle, from production to its application in water treatment, as well as recycling. The physicochemical properties of the biochar, in particular, are used as indicators during the decision-making phase. In general, the algorithm used (artificial neural networks, support vector machine) transforms the obtained data into an index that aids in selecting the most appropriate biochar for water treatment. The chosen biochar can be used directly if it meets certain standards for remediation, or it can be further functionalised or activated (physically or chemically). The latter will be re-characterised and evaluated further using artificial neural networks, and the process will be repeated until a biochar meeting water treatment standard is obtained (Medeiros et al. 2022). Additionally, the use of artificial neural networks and machine learning strategies can assist in determining the reusability and reapplication of spent biochar in water treatment. Table 15 illustrates sample applications of approaches based on artificial neural networks, either alone or hybrid.

**Table 15:** Predictive models, independent and dependent variables, and model performance indicators for the application of biochar in removing different pollutants from water

Feedstock	Target pollutant(s)	Predictive model	Parameters optimised	Optimised response variable (s)	Model performance indicators	References
Sawdust of white spruce (*Picea glauca*), canola (*Brassica napus*) and wheat (*Triticum aestivum*) straw	Lead (II)	Principal component analysis	Feedstock type, pyrolysis temperature, steam activation	Physical and chemical properties of the biochar	Multiple linear regression	Kwak et al. (2019)
Corn straw	Chlortetracycline HCl	Principal component analysis	pH, initial drug concentration, and coexisting anions	% Removal	Pearson’s correlation coefficient	Zhao et al. (2021d)
Antep pistachio *(Pistacia vera L.)* shells	Lead (II)	Artificial neural networks	Adsorbent dosage, initial concentration of lead (II) ions, initial pH, operating temperature, contact time	% Removal	Linear regression and minimum mean squared error	Yetilmezsoy and Demirel (2008)
Seaweed (*Ulva reticulata)*	Reactive red 120	Adaptive Neuro-Fuzzy Inference System	Bed depth, flow rate, initial dye concentration	% Removal of the dye	Error analysis, hybrid fractional error function, average relative error, Marquardt’s per cent standard error deviation, absolute average relative error, *R*^2^, *R*^2^-adjusted	Lenin Sundar et al. (2021)
Rice straw	Naproxen	Artificial neural networks	pH, adsorbent dose, contact time	% Removal of naproxen	Correlation coefficient (*R*^2^)	Bhattacharya et al. (2021)
Agricultural residues	Diclofenac, naproxen, and ibuprofen	Artificial neural networks	pH, concentration of the pollutants	% Removal	*R*^2^ values and mean squared error	Mojiri et al. (2019)
Date seed	Lead (II), copper (II), nickel (II)	Artificial neural networks—six machine learning approaches	pH, ionic strength, initial concentration of the pollutants, contact time	Adsorption capacity	*R*^2^ values and mean squared error	El Hanandeh et al. (2021)
Leaves of jackfruit, mango, and rubber plants	Cadmium (II)	Artificial neural networks in combination with genetic algorithm	Aqueous phase pH, initial cadmium (II) concentration, adsorbent dose, time, temperature	% Removal of cadmium (II)	*R*^2^ values, mean squared error, absolute average relative error, and standard deviation	Nag et al. (2018)
Rambutan (*Nephelium lappaceum*) peel	Copper (II)	Artificial neural networks, ANFIS and multiple MLR	Contact time, temperature, biochar discharge, initial copper (II) concentration,	Adsorption capability	Pearson’s correlation, root mean square error or mean absolute error	Wong et al. (2020)
Date palm	Ortho-cresol and phenol	Artificial neural networks and nonlinear regression generalised decay-function	Flow rate, bed depth, time, mass of the bed, initial concentration	Residual concentrations of both contaminants and breakthrough	*R*^2^ and root mean square error	Dalhat et al. (2021)

This mainly includes approaches based on principal component analysis and artificial neural networks

#### Mechanisms of pollutant biosorption

As previously stated, biochar can be produced via thermochemical conversion from a variety of carbonaceous materials under a variety of operating conditions such as reactor design, operating temperature, heating rate, residence time, flow gas type, and pre-/post-activation treatments (Chen et al. 2021c). Subsequently, the use of biochar may take on a variety of forms. Adsorption occurs when an adsorbate is adsorbed onto the surface of an adsorbent until an equilibrium (saturation) plateau is reached.

The process is divided into three distinct regions; the first, known as the clean zone, is devoid of adsorption. The second region is referred to as the mass transfer zone, which is where the adsorption occurs. The third zone is the exhausted zone, which is reached when equilibrium is achieved. The saturated region expands during the process, while the clean zone contracts. The mass transfer zone is impacted when the adsorbate concentration is increased; otherwise, it remains unaffected (Dai et al. 2019). This process is repeated until the adsorbent reaches saturation. Indeed, physisorption is a non-specific process characterised by mild attraction forces between the adsorbate and the biochar surface; in this scenario, multilayer adsorption is possible.

The physical mechanisms are mostly modulated by the polarity of the adsorbate molecule and the adsorbent surface. Surface adsorption, electrostatic interaction, hydrogen bonding, hydrophobic interaction, π–π stacking, and van der Waals forces are all examples of physical mechanisms (Sophia A and Lima 2018). Chemisorption, on the other hand, is distinct and is established by more expressive forces resulting from the formation of chemical bonds between the biochar and adsorbate via electron sharing. Monolayer adsorption can be demonstrated in this scenario (Deng et al. 2017). Tables 16 and 17 describe various adsorption mechanisms for removing inorganic and organic pollutants using various biochar-based materials.

**Table 16:** Loading capacities of different biochar-based materials towards different inorganic pollutants

Raw feedstock	Temperature	Target contaminant	Pre-treatment	Post-treatment	Maximum adsorption capacity (mg g^−1^)	Adsorption mechanism	Reference
Pomelo peel	250 °C	Silver (I)	Drying and impregnation with phosphoric acid	Pristine	137.4	Chemical adsorption	Zhao et al. (2018a)
Marine macro-algal	500 °C	Copper (II)	Immersing with iron (III) chloride	Pristine	69.37	Chemical adsorption	Son et al. (2018)
Corn straw	700 °C	Cadmium (II)	Washing and drying	Pristine	73.32	Cation-π interaction	Wang et al. (2018d)
Cauliflower leaves	600 °C	Copper (II)	Oven drying	Pristine	53.96	Electrostatic attraction, physisorption, ion exchange, and precipitation	Ahmad et al. (2018)
Corn Straw	800 °C	Cadmium (II)	Drying	Modification with ferric nitrate and calcination 800 °C	46.9	Chemisorption, Electrostatic interaction, and monolayer adsorption	Khan et al. (2020a)
Rice husk	450–500 °C	Chromium (VI)	Acid or alkali treatment	Modification with polyethyleneimine	435.7	Chemical reduction and adsorption	Ma et al. (2014)
Waste mangosteen shells	350–700 °C	Chromium (VI)	Washing and drying	Modification with zinc chloride	341.4	Electrostatic interaction, surface complexation, ion exchange, and physical adsorption	Shan et al. (2020)
Rice straw	700 °C	Cadmium (II)	Crushing	Pristine	65.4	Cation exchange, precipitation, physical adsorption, electrostatic attraction, and cation-π interaction	Deng et al. (2019)
Corn straw	300 °C	Chromium (VI)	Washing and drying	UV-radiation	20.04	Surface complexation	Peng et al. (2018a)
Alternanthera philoxeroides	600 °C	Lead (II)	Washing and drying	Pristine	257.12	Precipitation, complexation, and ion exchange	Yang et al. (2014)
Rice husk	600 °C	Cadmium (II)	Mixing with iron (III) nitrate	Soaking with potassium permanganate	79	Chemical adsorption, oxidation, π-interaction, and complexation	Sun et al. (2019)
		Lead (II)			148		
Bamboo wood	600 °C	Silver (I)	Oven drying	Treating with hydrogen peroxide + nanoparticles zero-valent iron	1217	Innersphere complexation and electrostatic attraction	Wang et al. (2017e)
Muskmelon peel	600 °C	Zinc (II)	Washing and drying	Pristine	72.99	Liquid-film, and intraparticle diffusions	Khan et al. (2017)
		Copper (II)			78.74		
Papermill sludge	720 °C	Arsenic (V)	Oven drying and acid washing	Pristine	34.1	Chemisorption	Cho et al. (2017)
Peanut shell	400 °C	Cadmium (II)	Washing, drying, and milling	Treating with hydrated manganese oxide	10	Non-specific outer-sphere surface complexation, and Specific innersphere complexation	Wan et al. (2018)
Willow wood	550 °C	Nickel (II)	Washing and drying	Pristine	9.8	Surface complexation	Wang et al. (2020b)
		Copper (II)			12.2		
		Cadmium (II)			35.2		

Different biomass-based feedstocks were studied against the targeted contaminants. Pre- or post-treatments were also investigated along with the temperatures at which the biochar was prepared. The maximum adsorption capacity in mg g^−1^ and adsorption mechanism are provided

**Table 17:** Loading capacities of different biochar-based materials towards different organic pollutants

Raw feedstock	Temperature	Production pathway	Target contaminant	Maximum adsorption capacity (mg g^−1^)	Adsorption mechanism	References
Orange peel waste	150–600 °C	Microwave pyrolysis via carbon dioxide and steam activation	Congo red	136.00	Electrostatic interaction	Yek et al. (2020)
Hickory chips	600 °C	Pyrolysis followed by impregnation with iron (II, III) oxide	Methylene blue	500.5	Electrostatic interaction and π–π interaction	Li et al. (2020e)
Switchgrass	900 °C	Pyrolysis	Orange G	38.2	Electrostatic interaction, and π–π interaction	Park et al. (2019)
Frass of mealworms	800 °C	Pyrolysis	Malachite green	1738.6	Electrostatic interaction, hydrogen bonding, and π–π interaction	Yang et al. (2019b)
Corncob	400 °C	Pyrolysis followed by impregnation with triethylenetetramine, and treatment with sulphuric acid	Sunset yellow	77.1	Electrostatic interaction	Mahmoud et al. (2020)
Macroalgae (*Undaria pinnatifida*)	800 °C	Chemical functionalisation with potassium hydroxide followed by pyrolysis	Rhodamine B	533.8	Electrostatic interaction, hydrogen bonding, van der Waals forces, and π–π interaction	Yao et al. (2020)
			Methylene blue	841.64		
			Malachite green	4066.96		
Corn straw	500 °C	Nitric acid treatment, sodium hydroxide activation, followed by pyrolysis iron (III) chloride modification	Malachite green	515.8	Electrostatic attraction	Eltaweil et al. (2020)
Swine manure and fly ash	700 °C	Pretreatment of fly ash with sodium hydroxide, mixing with swine manure and pyrolysis	Methylene blue	131.6	Electrostatic interaction, and π–π interaction	Wang et al. (2020c)
Tapioca peel waste	800 °C	Mixing of pyrolysed feedstock with thiourea and pyrolysis	Rhodamine B	33.1	Electrostatic interaction, and Hydrogen bonding	Vigneshwaran et al. (2021)
			Malachite Green	30.18		
Switchgrass	900 °C	Pyrolysis	Congo red	22.6	Electrostatic interaction, and π–π interaction	Park et al. (2019)
Wakame (macroalgae)	800 °C	Chemical functionalisation with potassium hydroxide followed by pyrolysis	Malachite green	4066.9	Electrostatic interaction, π–π stacking, hydrogen bonding, and van der Waals force	Yao et al. (2020)
Rice straw	500 °C	Pyrolysis followed by wet attrition	Methylene blue	90.91	Electrostatic interaction	Abd-Elhamid et al. (2020)
			Crystal violet	44.64		
Corncob	400 °C	Pyrolysis followed by partial oxidation and amination	Congo red	89.3	Chemisorption	Faheem et al. (2019)
Chicken manure	500 °C	Pyrolysis	Phenols	106.2	Electrostatic interaction, π–π interaction, and hydrogen bonding	Thang et al. (2019)
			2,4-dinitrophenol	148.1		
Alfalfa	700 °C	Pyrolysis followed by nitric acid treatment, acid pickling, and reheating	p-Nitrophenol	49.25	Hydrogen bonding, and π–π interaction	Chen et al. (2019)
Malt bagasse	500–900 °C	Pyrolysis followed by zinc chloride activation; pyrolysis followed by hydrochloric acid treatment	2-chlorophenol	150.0	Electrostatic interaction	Machado et al. (2020)
Alfalfa	650 °C	Pyrolysis	Bisphenol A	63.0	π–π interaction, and hydrophobic interaction	Choi and Kan (2019)
Rice husk	450 °C	Pyrolysis followed by potassium hydroxide functionalisation	Phenols	179.0	π–π interaction, and hydrogen bonding	Shen et al. (2020)
Furniture waste	700 °C	Pyrolysis followed by coating with fulvic acid	4-chlorophenol	133.0	Hydrogen bonding, and π–π interaction	Wu and Chen (2019)
Macroalgae	800 °C	Pyrolysis followed by hydrochloric acid treatment	Pyrene	0.19	Hydrophobic interaction	Qiao et al. (2018)
Corn straw	500–800 °C	Pyrolysis followed by mixing with potassium hydroxide, and then pyrolysis	Naphthalene	450.4	π–π interaction, and pore filling	Qu et al. (2021)
Rice husk	500 °C	Pyrolysis followed by iron (II) sulphate modification	Phenanthrene	97.6	π–π interaction, hydrophobic interactions, and pore filling	Guo et al. (2018a)
Macroalgae	800 °C	Zinc chloride and iron (III) chloride modification, followed by pyrolysis, and hydrochloric acid treatment	Naphthalene	90.0	π–π interaction, partitioning effect, and pore filling	Cheng et al. (2020b)
Alfalfa	650 °C	Pyrolysis	Sulphamethoxazole	90.0	π–π interaction, and hydrophobic interactions	Choi and Kan (2019)
Rice straw	550 °C	Pyrolysis followed by hydrochloric acid treatment	Benzoic acid	7.97	π–π interaction, and van der Waals attractions	Singh et al. (2020)
Microalgae	750 °C	Pyrolysis	Tetracycline	132.8	Hydrophobic interactions, and π–π interaction	Choi et al. (2020)
Grape pomace	350 °C	Pyrolysis	Cymoxanil pesticide	161.0	Hydrophilic interactions	Yoon et al. (2021)
Corn cob	600 °C	Pyrolysis followed by hydrofluoric acid treatment	2,4-dichlorophenoxyaceti acid (2,4-D) herbicide	34.4	Hydrogen bonding, pore filling, and π–π interaction	Binh and Nguyen (2020)
Lotus seedpod	500–650 °C	Pyrolysis followed by potassium hydroxide activation and pyrolysis	17β-estradiol hormone	183.6	π–π interaction, and electrostatic interaction	Liu et al. (2020b)

Different biomass-based feedstocks were studied against the targeted contaminants. Pre- or post-treatments were also investigated along with the temperatures at which the biochar was prepared. The maximum adsorption capacity in mg g^−1^ along with the adsorption mechanism are provided

As demonstrated, the adsorption process is not confined to a single mechanism; rather, multiple mechanisms can occur concurrently and be complementary to one another (Zhou et al. 2019a). Furthermore, it has been demonstrated that the physicochemical properties of biochar-based materials, such as specific surface area and oxygen-to-carbon ratio, play a critical role in the adsorption process. In general, specific surface area is critical for both inorganic and organic adsorption of pollutants, whereas a higher oxygen-to-carbon ratio favours inorganic adsorption due to the increased number of oxygen-containing functional groups and cation-exchange capacities (Jin et al. 2021). At the same time, higher aromaticity and hydrophobicity are associated with organic dye adsorption (Choudhary et al. 2020).

##### Surface adsorption

Chemical bonds are formed between the adsorbate and adsorbent surface in this mechanism as the adsorbate diffuses into the adsorbent's pores. Operationally, the pyrolysis temperature affects the volume of pores and surface area of the biochar (Ambaye et al. 2020b). Zhang et al. (2021b) investigated the process of cadmium adsorption onto engineered biochar prepared by impregnating raw bamboo residues with potassium phosphate at temperatures ranging from 350 to 950 °C. The results indicated that the aromatization and graphitization degrees of the potassium phosphate-biochar increased as the temperature increased. Additionally, the surface area and phosphorus-containing functionalities increased initially. They then decreased, indicating that the pyrolysis temperature of 550 °C obtained the best pore structure and surface chemical properties, resulting in optimal energy recovery and a maximum loading capacity of higher than 289.0 mg g^−1^.

##### Electrostatic interaction

This is the most critical mechanism involving the attractive electrostatic interaction between the positively or negatively charged biochar and the oppositely charged adsorbate. The pH, ionizable species of pollutants, ionic strength, zeta potential measurement, and point of zero charge values all contribute to determining the biochar's ability to attract or expel contaminants (Li et al. 2019d). For example, the initial pH values greatly impact the adsorptive performance of the developed microwave carbon dioxide-activated biochar and microwave steam-activated biochar. In the pH range of 3–7, the lower concentration of cations facilitated the binding of negatively charged Congo red dye molecules to the adsorbent surface. In contrast, anions in the alkaline environment competed with Congo red molecules, hindering their adsorption onto the modified-biochar surfaces. Under acidic conditions, the Congo red removal percentages of 89% (Qmax = 91 mg g^−1^) and 93% (Qmax = 136 mg g^−1^) for microwave carbon dioxide-activated biochar and microwave steam-activated biochar, respectively, were achieved (Yek et al. 2020). Additionally, Igalavithana et al. (2017) stated that electrostatic interaction was the primary mechanism governing the removal of potentially toxic metals via biochar.

##### Van der Waals forces

In this mechanism, the electronegative atoms (e.g. nitrogen, oxygen, and chlorine) found in the neutral molecules may attract the electron cloud via a covalent bond with a less electronegative neighbouring atom, thereby enhancing the molecule's dipolar nature (Roy et al. 2015). When the positive end of one molecule is aligned with the negative end of another, the partial positive (δ+) and negative (δ−) charges within the molecule are stimulated. Numerous factors affecting the structural composition of the biochar, the speciation of the adsorbate, and the surface properties of both biochar and adsorbate all contribute to the Van der Waals forces. Hydrogen bonding is a type of dipole–dipole interaction that occurs when an electronegative atom interacts with a hydrogen atom connected to another electronegative atom. It is established through the allocation of hydrogen atoms between electronegative atoms (Choi et al. 2019). The strength of hydrogen bonding is determined by the atoms' electronegativity. Zhou et al. (2019a) demonstrated that hydrogen bonding is involved in the adsorption of ciprofloxacin and sparfloxacin onto iron (II, III) oxide/graphene-modified citrus peel biochar. It was ascribed to the increased electronegativity of fluorine atoms in the structure of these molecules and the oxygen-containing groups on the modified biochar surface.

##### Ion exchange

The ion exchange process is defined as the reversible exchange of ions between biochar (an insoluble solid phase) and adsorbate (liquid phase). Inherently, biochar's cation/anion exchange capacity refers to the total deposition of cations or anions on the biochar surface via the displacing of corresponding amounts of other ions (exchangeable ions). This scenario is primarily based on the exchange of protons and ionised cations with dissolved salts on the surface of the biochar. Biochar's loading capacity is determined by its size and surface functional groups (Gurav et al. 2021). Wu et al. demonstrated that magnesium-coated coconut shell biochar significantly increased its adsorptive capacity for lead and cadmium ions by 20 and 30 times, respectively, when compared to pristine biochar. The recorded Qe values of the magnesium-coated biochar produced at 400 °C towards lead and cadmium ions were 49 and 59 times greater than those of pristine biochar produced at the same pyrolysis temperature, respectively. This was primarily attributed to mechanisms of ion exchange and precipitation (Wu et al. 2021).

##### Surface precipitation

Surface precipitation occurs when insoluble mineral precipitates form on the surface of an adsorptive material, most notably biochar, which is composed of cellulose and hemicelluloses degraded at temperatures greater than 300 °C and has an alkaline property. Changes in the solution pH or other components (e.g. mineral, enzymatic, and polymeric compounds) on the biochar surface can be used to simulate the reaction (Ambaye et al. 2020b). According to Puga et al. (2015), biochar produced from sugarcane and straw dust can enhance cadmium and zinc metal ion precipitation. However, the susceptibility of biochar surface precipitation may be largely dependent on the pyrolysis temperature, implying that additional research on optimising the pyrolysis temperature is strongly recommended. Zama et al. (2022) enhanced the adsorption of arsenate ions using rice husk/aspen wood-based biochar by beneficially precipitating calcium/arsenic compounds. This was accomplished by precipitating arsenic in solution using calcium and then supplementing with unmodified biochar to increase the yield of the removal process. Notably, arsenate concentration in the working solution was successfully reduced by 58.1%.

##### Complexation

This mechanism results in the formation of multi-atom structures when metal ligands interact with biochar to form complexes. The formation of complexes on the surface of the biochar demonstrates the adsorption of water pollutants as metal ions. A complex is a polyatomic molecule that contains one or more core atoms enveloped by ligands. Chemically, coordination reactions promote the formation of Lewis acid–base complexes, in which the metal ion acts as a Lewis acid (electron acceptor) and the ligands act as Lewis bases (electron donors) (Hosmane 2017). The oxygen-containing functional groups (e.g. carboxyl, lactonic, and phenolic) formed during low-temperature pyrolysis enhance the binding of metal ions to biochar.

Moreover, the presence of oxygen accelerates the oxidation of the biochar surface, which promotes enhanced metal complexation. Chelation is a specific aspect of complexation that involves the formation of complexes between organic molecules and a single central atom. It usually occurs when a metal ion reacts with a complexing agent, resulting in the formation of a ring structure. To summarise, surface complexation, including coordination and chelation, is viewed as a critical pathway for pollutant removal via biochar (Cui et al. 2016b; Ifthikar et al. 2017). Sun et al. concluded that the results of X-ray photoelectron spectroscopy confirmed the successful impregnation of manganese oxides on the potassium permanganate-treated magnetic biochar, which resulted in enhanced oxygen functional groups on its surface. This demonstrated that the adsorbed lead initially forms monodentate complexes (–O–Pb^+^), which are then tuned to form –O–Pb–OH in the solution, increasing the proportion of M–OH on the adsorbent surface (Sun et al. 2019).

##### Partitioning

Adsorption partitioning mechanisms are comparable to those used in solvent extraction, which involves the separation of molecules based on their relative solubility in two distinct immiscible liquids. As a result, nonpolar molecules, primarily organic pollutants, would dissolve in non-carbonised adsorbents or organic fractions of low-temperature pyrolysis biochar (Xiang et al. 2020b). The partitioning mechanism is dependent on the non-carbonised biochar (amorphous or crystalline carbon) and the carbonised crystalline and graphene fractions of the biochar (Ambaye et al. 2020b). Xiang et al. (2020b) investigated the mechanisms governing the adsorption of five volatile organic compounds (acetone, cyclohexane, chloroform, ethanol, and toluene) onto hickory wood-based biochar. They concluded that the partitioning mechanism is critical for removing organic species when biochar with organic fractions is used.

##### Pore-filling

Structurally, the high surface area associated with the presence of mesopores and micropores may enhance the biochar's adsorptive performance. The physicochemical properties of biochar, as well as the polarity of organic pollutants, are critical for the adsorption process to be effective (Mandal et al. 2017). According to the international union of pure and applied chemistry's structural classification, the pore widths are classified as follows: less than 2 nm, 2–50 nm, and greater than 50 nm for micropores, mesopores, and macropores, respectively (Thommes et al. 2015). Binh and Nguyen investigated the adsorption mechanisms of 2,4-dichlorophenoxyacetic acid onto corn cob biochar. They reported that the molecular dimensions of the target pollutant of 1.54 × 0.56 × 0.22 nm facilitated its adsorption onto the biochar through its narrow pores. This was strongly supported by the fact that the surface area and total pore volumes decreased significantly at the end of the adsorption process (Binh and Nguyen 2020).

##### Hydrophobic interactions

To reduce the overall interfacial area between hydrophobic pollutants and water molecules, it is necessary to emphasise non-covalent hydrophobic interactions. Nonpolar pollutants, distinctly, tend to aggregate in aqueous solutions. The hydrophobic interactions are triggered by a shift in the system's entropy. The ordered behaviour of water molecules is described by the system's reduced entropy. When nonpolar species enter the system, they impose a constraint on the water molecules, limiting their freedom of orientation. Therefore, the undesirable interaction is avoided by limiting the interaction of water molecules with the nonpolar surface, which results in the aggregation of nonpolar pollutants and hydrophobic biochar in an aqueous medium (Roy et al. 2015). According to Zhou et al., the removal of ciprofloxacin and sparfloxacin using iron(II, III) oxide/graphene-modified biochar was significantly attributed to hydrophobic interaction due to the increased hydrophobicity, which resulted in a strong interaction between the adsorbate and adsorbent surface (Zhou et al. 2019a).

##### π–π stacking and other π-interactions

These are the non-covalent attractive interactions that exist between aromatic rings due to the presence of π-bonds. In contrast to conventionally formed covalent bonds, the π-orbitals of two molecules do not interact. One of two organic molecules is aromatic, which means it contains π-electrons and forms a complex with the other molecule via non-covalent bonding forces. Other non-covalent bonding forces that compromise the π system include the n–π interaction, in which ions act as electron donors and the aromatic ring on the biochar surface acts as an electron acceptor (Roy et al. 2015). According to Dong et al. (2018), the aromatic sheets found in magnetic biochar nanoparticles facilitated the removal of 17β-estradiol via π–π interactions. Additionally, during the elimination of ciprofloxacin and sparfloxacin using the iron(II, III) oxide/graphene-modified biochar, the graphene on the modified biochar acted as π-electron donors, whereas the antibiotics acted as π-electron acceptors, due to the high propensity of the fluorine group of the benzene ring to pull electrons (Zhou et al. 2019a).

#### Summary

A proposal has been established and shared as part of the 2030 agenda for sustainable development, which has been approved by all United Nations member states since 2015. Fundamentally, this agenda entails adopting the 17 sustainable development goals by all countries in a comprehensive alliance. This alliance recognised that eliminating all deprivations requires a coordinated approach that prioritises well-being, education, equity, and economic development—all while addressing environmental concerns regarding water, food, and energy (Srivastav and Kumar 2021; Wilson and Velis 2015). Access to clean water and sanitation became a stand-alone sustainable development goal in 2015. As such, the sixth sustainable development goal, to ensure availability and sustainable management of water and sanitation, has been identified as critical (Brookes and Carey 2015; Juárez 2020; Gwenzi et al. 2017). According to the United Nations report, even though universal access to safe drinking water, sanitation, and hygiene is critical for coronavirus disease 19 recovery, billions of people still lack access to safe drinking water, sanitation, and hygiene, and 129 countries are not on track to managing water resources sustainably by 2030 (UN 2020). Additionally, the world health organization estimates that approximately 829,000 people will die each year from diarrhoea due to unsafe drinking water, sanitation, and hand hygiene (WHO 2007, 2019).

As previously stated, recycling biowaste into biochar is not only environmentally beneficial, but also aligns with the sustainable development goals in terms of waste management and thus contributes to the circular economy's advancement. Nonetheless, it is necessary to consider the potential health and environmental risks and restrictions associated with using these types of feedstocks. Despite the potential benefits of using biochar in various environmental applications, concerns about the production and fate of biochar are gradually increasing. To begin with, biochar production systems have the potential to release greenhouse gases into the atmosphere, with associated negative ecological and health consequences if not designed properly (Kamali et al. 2021; Harsono et al. 2013). Pyrolysis also produces volatile organic compounds, polycyclic aromatic hydrocarbons, dioxins, and furans. The utilisation of appropriate technology, as well as a thorough understanding of the dimensions involved in the production of biochar, are critical. Additionally, life cycle assessments must be conducted to determine the impact of each biochar system on the environment.

Another aspect of the process is the fate of the spent biochar, which is also questionable and could present problems if not managed properly. As previously demonstrated, pollutants can be adsorbed via chemi- or physisorption mechanisms, a potentially reversible process, particularly in physical adsorption processes. This reversibility may result in the re-release of pollutants into the treated water. Incinerating the adsorbent is not an option as it would re-emit carbon and other toxic pollutants into the atmosphere, losing the carbon sequestration objective and necessitating additional mitigation measures and costs. In this case, recovering and regenerating the biochar adsorbent over multiple sorption cycles may be a better option (Krasucka et al. 2021). Numerous methods for sorbent regeneration have been reported, including using solvents, acids and bases, microwaves, and thermal regeneration (Kamali et al. 2021).

However, for the purpose of carbon sequestration, any regeneration technique that subjects the biochar to further thermal degradation or oxidation cannot be utilised to prevent further release of the sequestered carbon. Furthermore, the safe disposal of spent biochar in dedicated landfills can serve as a long-term storage reservoir under certain conditions. Additionally, spent biochar involved in removing nutrition-based pollutants, such as ammonia and phosphorus, can be safely applied to soils with further agronomic benefits.

To conclude, our analysis shows that biochar represents an ultimate solution for several environmental concerns. The utilisation of biochar as an adsorbent in water remediation prior to long-term storage is a value maximisation strategy that can be technically viable and economically rewarding.

### Soil remediation

A growing body of research indicates that biochar amendments may be a viable strategy for alleviating soil contamination by immobilising organic and inorganic contaminants (Kumar et al. 2021b). Biochar's qualitative attributes as a soil amendment vary significantly depending on the feedstock sources and pyrolytic conversion parameters employed. Biochar materials derived from various sources have exhibited diverse capabilities and effectiveness for soil contaminant stabilisation. Soil organic pollutant remediation is often accomplished by sorption and degradation mechanisms, whereas inorganic pollutant remediation is accomplished through sorption and chemical precipitation (Ji et al. 2022). Biochar produced at a high temperature and with a high sorption capacity is more appropriate for the rapid fixation and adsorption of soil organic/inorganic contaminants. Low-temperature biochar with a suitable nutrient concentration, on the other hand, is preferred by soil microorganisms because it accelerates the biodegradation process (Li et al. 2020a; Ni et al. 2020b).

#### Remediating soil organic contaminants

Organic soil pollutants include pesticides, herbicides, various antibiotics, polycyclic aromatic hydrocarbons, polychlorinated dibenzofurans, polychlorinated dibenzodioxins, and polychlorinated biphenyls. These organic pollutants pose a serious threat to environmental preservation, as well as food and health safety. The addition of biochar to contaminated soils facilitates the direct interaction with organic pollutants and soil microorganisms (Guo et al. 2020b). Numerous studies have demonstrated the efficacy of biochar in stabilising organic pollutants via various physical and chemical sorption mechanisms. Electron donor and acceptor, pore filling, electrostatic and hydrophobic interactions, surface adsorption as well as hydrogen bonding all contribute to the biochar sorption capability of organic contaminants (Guo et al. 2020b; Abbas et al. 2018b). Khorram et al. explored the use of biochar to remediate soil that is contaminated with organic pesticide residues (Khorram et al. 2017). Lonappan et al. reported that biochar produced from pig manure (anionic biochar) is efficient for methylene blue sorption (cationic organic contamination) (Lonappan et al. 2016). According to Wathukarage et al., biochar produced from peanut shells is ideal for phosphate remediation due to its unique qualities of large surface area and microporosity (Wathukarage et al. 2019).

In addition to sorption as a mechanism, biochar facilitates soil remediation via promoting enhanced microbial mineralization of organic contaminants (Guo et al. 2020b). Under natural conditions, microorganisms take a long time to degrade organic pollutants (Ji et al. 2022). By incorporating biochar into the soil, the nutritional content is increased, which means that microbial activity enzymes (such as alkaline phosphatase, urease, and others) are increased (Pokharel et al. 2020b). This leads to a more diverse soil microbial community (Jaiswal et al. 2018).

Zhang et al. (2018c) stated that the composition of the microbial community most likely shifts from fewer fungi to a greater fungal-to-bacteria ratio and more gram-positive bacteria than gram-negative bacteria. Furthermore, the researchers investigated soil polycyclic aromatic hydrocarbon biodegradation using biochar derived from various plant residue sources and observed that the biodegradation effects were similar in terms of enzyme activity and microbial community composition. Another study conducted by He et al. (2018b) reported that the addition of pig manure biochar to low organic matter soil enhanced the biodegradation of phthalates by a greater percentage compared to bamboo wood biochar. Generally, biochar with higher organic carbon content and a larger surface area is preferable for facilitating the biodegradation of persistent organic pollutants and pesticide residues in soil (Ji et al. 2022). Notably, biochar may contain active organic compounds, which are hazardous to soil microorganisms; also, the adsorption and hydrolysis of signalling molecules by biochar may alter the soil microbial community (Luo et al. 2022).

#### Remediating soil inorganic contaminants

Soil contamination by heavy metals can occur from various sources, including mining activities, industrial effluents, and pesticides, and is regarded as one of the world's most pressing issues (Cao et al. 2022; Qiu et al. 2020). Most heavy metals end up in either water or soil and are mostly non-biodegradable, such as cadmium, chromium, lead, along with others (Liu et al. 2020c). Accumulation in the soil, water, as previously discussed, and plants, even at low concentrations, will not only harm humans and animals but will also damage the entire ecosystem (Wang et al. 2019c).

Heavy metals in contaminated soils are typically treated using conventional methods that focus on their removal via washing, leaching, and extraction. Unlike these strategies, biochar amendments stabilise heavy metals in the soil by converting them to a form that is less soluble and bioaccessible. The mechanisms that facilitate heavy metal stabilisation include electrostatic attraction, ion exchange, surface complexation as well as precipitation. When biochar is applied to contaminated soils, heavy metals are adsorbed onto the biochar surface and potentially are converted to hydroxide, phosphate and carbonate precipitates. As the fraction of heavy metals that is water-soluble is minimised, the potential for uptake and bioaccumulation by soil microorganisms is significantly reduced (Guo et al. 2020b).

Biochar application in soil contaminated with chromium (VI) has been investigated for many years, where several recent studies have demonstrated its remediation efficacy (Rajapaksha et al. 2018; Huang et al. 2020; Rafique et al. 2019; Khan et al. 2020b). Murad et al. recently reported that when they used biochar derived from peanut shell waste for the removal, immobilisation, and/or adsorption of chromium (VI) from soil and water, the removal/remediation effectiveness reached 79.35% (Murad et al. 2022). In a pot experiment, manganese oxide-modified biochar derived from corn straw was used to remediate soil for arsenic as well as plant roots for iron-manganese plaque through adsorption. The researchers reported that manganese oxide-modified biochar increased rice growth and decreased the arsenic concentration in brown rice (Yu et al. 2017).

It is important to note that biochar application may be ineffective for inorganic pollutant remediation in particular circumstances, such as when high acidity soil contains high amounts of various heavy metal pollutants. In such extreme cases, biochar treatment could be used in conjunction with, or subsequent to, another management strategy or approach to resolving the issue (Jiang et al. 2021). A combination of biochar produced from rice straw and lime was used to treat lead-contaminated soil; the mixture increased soil pH and decreased the lead content in brown rice (Li et al. 2018a). Penido et al. (2019) conducted a greenhouse experiment to determine the effect of adding sewage sludge to biochar derived from eucalyptus wood. The researchers reported a rise in soil pH and a decrease in lead, cadmium, and zinc bioavailability. Several recent investigations have indicated that a promising, highly reactive material known as nano-zero-valent iron may be exploited for inorganic pollution remediation (Diao et al. 2020a, b, 2021; Li et al. 2018b). Qian et al. (2022) combined the latter material with biochar for cadmium and lead immobilisation in contaminated soil, achieving a stabilisation efficiency of 80%.

#### Summary

This subsection provided a concise overview of the role of biochar in promoting soil remediation from organic and inorganic contaminants. Overall, our analysis demonstrated the efficacy of biochar in eliminating soil contaminants in most tests and research, the majority of which were conducted in the laboratory and a few in the field. Short- and long-term field tests should be conducted to gain a better understanding. Models should be constructed to simulate field environmental conditions that vary according to soil type, soil texture, pH, salinity, and region climate, among other variables. Enhancing the performance of biochar in terms of sorption, surface area, and nutrient content by optimising  processing conditions as well as mixing it with other highly reactive materials, such as nanoscale compounds, could be a viable way of making it more effective, economical and environmentally friendly. There is still much to explore, and additional research is necessary, particularly when employing biochar as a soil additive for contaminated soils.

## Construction

**Fig. 8:**
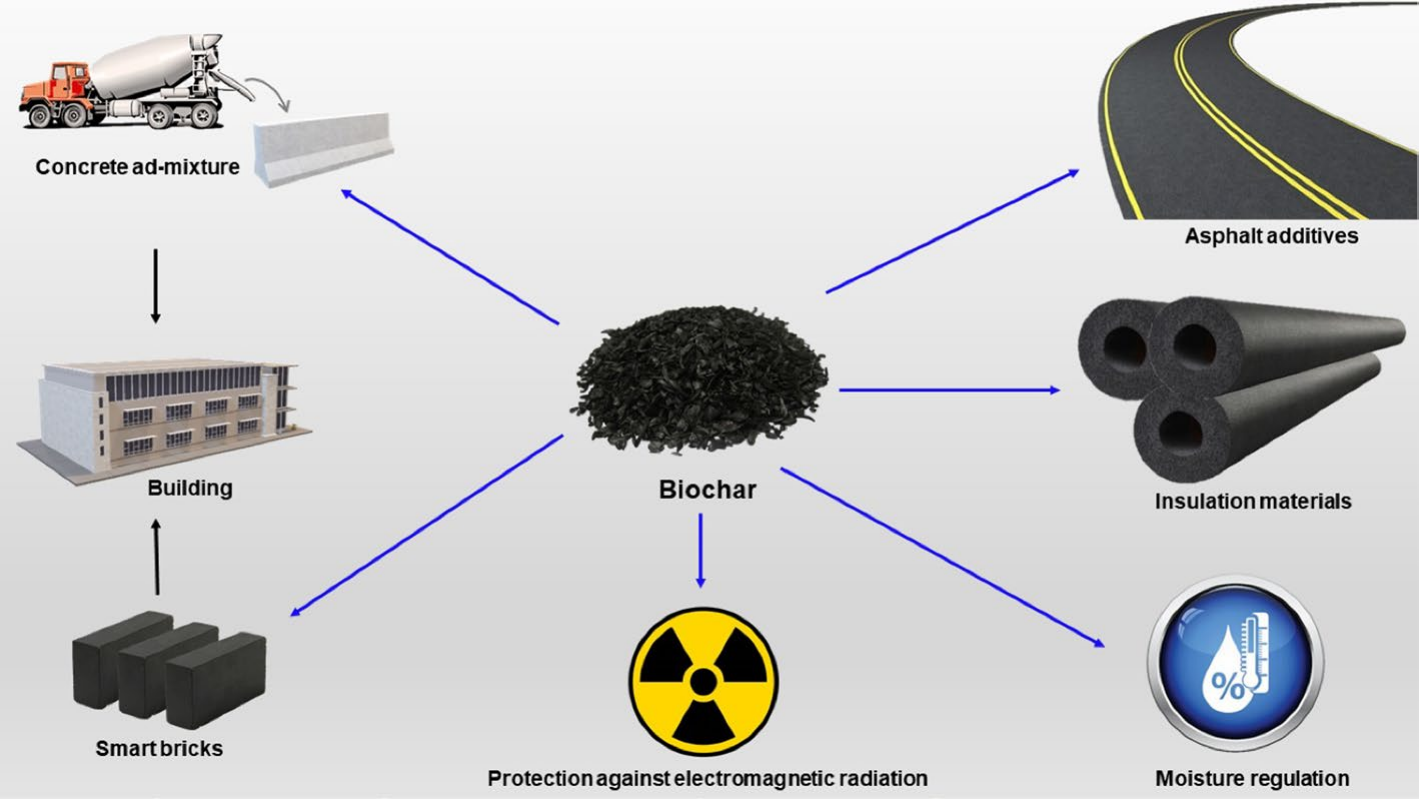
The various uses of biochar in construction as a potential carbon sink application. Biochar can be used as a cementitious additive in concrete and mortar. The potential for incorporating biochar into bricks has been recognised due to biochar's thermal insulating qualities when used in place of sand and aggregates, as well as its direct impact on weight reduction and carbon dioxide removal from the atmosphere. Finally, biochar has the potential to be used as an asphalt additive and to be incorporated in biocomposites for enhanced insulation, moisture regulation and electromagnetic radiation protection

In response to the growing carbon footprint of the construction industry, there is a greater demand for carbon dioxide emission management and reduction solutions. The construction industry's carbon dioxide emissions are influenced by several factors, including raw material processing, cement manufacturing, and, most importantly, construction. The potential use of biochar in construction, as shown in Fig. 8, has been thoroughly researched in the literature. Three primary qualities have been identified as indicators of its viability for use in construction: its high chemical stability, low thermal conductivity, and low flammability.

Chemical stability is critical to ensuring that no adverse chemical reactions occur when biochar is combined with other components in an asphalt or concrete mix. In general, concrete is known to be attacked by chemicals, reducing its durability. Additionally, asphalt degrades due to oxidation, which significantly impacts the longevity and stability of roads and pavements. Once combined with concrete or asphalt, biochar's chemical stability eliminates the potential of such damaging chemical reactions and provides long-term durability. Additionally, the low thermal conductivity increases the insulating capacity of buildings and structures. The primary component affecting this attribute is porosity, specifically the pore size distribution. Finally, reduced flammability is a critical safety requirement (Fawzy et al. 2021).

Furthermore, biochar's water-holding capacity has been demonstrated to provide appropriate hydration in cementitious admixtures, hence enabling better internal curing. This enhances durability, shrinkage resistance, crack resistance, and improves mechanical characteristics. Additionally, the literature generally indicates structural improvements associated with the introduction of biochar into cement-based composites, including improvements in mechanical properties such as compressive and flexural strength, ductility, as well as toughness (Fawzy et al. 2021). Biochar has shown great potential in being used in construction, offering several structural and functional advantages. Furthermore, the potential of incorporating biochar into the built environment for extended storage reinforces the notion that civil infrastructure serves as a solid carbon reservoir. This section will present the potential ways biochar can be utilised within construction.

### Biochar as an additive in cementitious materials

To save natural resources and promote the use of waste or recycled materials in the construction industry, governments recently established standards for the use of sustainable materials. Construction and industrial activities are primary sources of greenhouse gas emissions globally. Numerous approaches have been proposed in the literature to address this issue, including the utilisation of biomass and waste materials, as well as ashes (Mao et al. 2020), and the development of alkali-activated materials to replace Portland cement in concrete and mortars. Incorporating bio-based materials, in raw or ash forms, into the primary composition of mortar or concrete is currently limited to economically viable options.

Numerous studies have highlighted concern about the adverse effect of biomass on the durability of cementitious materials due to the alkaline nature of such materials. Another drawback of using biomass in cement composites is that the presence of hemicellulose, a source of sugars such as glucose and fructose, retards cement hydration. Furthermore, contaminants in rice husk ash have been identified as negative characteristics in mortar. Biochar is unique among bio-renewable resources in that it can address all of the challenges mentioned above concurrently. Table 18 highlights the most significant findings from previous research on biochar's use in building materials, particularly cement mortar and concrete.

**Table 18:** Biochar as an additive in cementitious materials. Various feedstock materials along with the pyrolysis operating conditions are reported

Feedstock	Pyrolysis conditions	Biochar as	Dosage	Adopted tests in mortar	Curing condition	Main outcomes	References
Mixture of woodchips of local forest	900 °C	Filler	**1**%, 2.5%	Density, flexural and compressive test, fracture energy	Water-cured	Slight reduction in compressive strength with increasing biochar dosage, an increase in flexural strength and fracture energy	Sirico et al. (2020)
Poultry litter, rice husk, PPI sludge	450, 500 °C , residence time: 20 min	Cement replacement	**0.1**–1%	Compressive, flexural, and splitting tensile tests, water absorption, Fourier transform infrared spectroscopy, X-ray diffraction, Scanning electron microscope	Moist curing	Effective at an early age, strength development of concrete	Akhtar and Sarmah (2018a)
Sugarcane bagasse	200 °C , slow pyrolysis, residence time: 2 h	Filler	2, 4, 6%	Apparent porosity, thermal conductivity, isothermal calorimetry, flexural test	50% relative humidity	Improvement of hydration, reduction in thermal conductivity by 25% due to the addition of 4% biochar	Yang et al. (2019c)
Softwood	700 °C	Filler	0.8, 1%	Flexural test, fracture energy	Water-cured	Promising improvement in strength and toughness of cement composite	Cosentino et al. (2019)
Dried distillers’ grain	500 and 600 °C	Sand and aggregate replacement	1, 2, 3, 5, 10, 12, 15%	Density, compressive test, sound absorption test, thermal conductivity	Ambient condition	10 and 15% of biochar leads to better sound absorption properties, at 2% biochar addition, thermal conductivity shows the highest reduction	Cuthbertson et al. (2019)
Rice husk	450 °C	Cement replacement	**0.1**–0.75%	Compressive and splitting tensile tests, water absorption, Fourier transform infrared spectroscopy, X-ray diffraction, Scanning electron microscope	Fog-cured	17% reduction in water absorption, enhancement of mechanical properties at optimum biochar dosage	Akhtar and Sarmah (2018a)
Wheat straw	650 °C, residence time: 4 h	Magnesium oxide and ammonium dihydrogen phosphate replacement	0.5, 1, 1.5%	Compressive and flexural tests, water absorption, porosity, water resistance, X-ray diffraction, the scanning electron microscope, Fourier transform infrared spectroscopy	Room temperature	Development of mechanical properties with increasing biochar content	Ahmad et al. (2020)
Grey borgotaro	700 °C	Filler, Cement replacement	0.8, 1, 1.5, 2, 2.5%	Mechanical tests, fracture energy	Water-cured	Increase in flexural strength and fracture energy in the optimum biochar percentage	Restuccia et al. (2020)
Masson pine wood	500, 700 °C heating rate of 10 °C min^−1^	Filler	1, 2, 5%	Isothermal calorimetry, X-ray diffraction, thermal gravimetric analysis, scanning electron microscope, compressive strength	Air-cured, carbon dioxide-cured	The incorporation of biochar as a green additive promotes the generation of hydration products, carbon dioxide curing of newly formed blocks accelerate cement hydration and carbonation	Wang et al. (2020d)
Wood sawdust	500 °C	Filler	0.25, 0.5, 1, 2%	Rheology, isothermal calorimetry, mechanical tests, water absorption	Moist cured, Air-cured	Effect of coarse biochar on the rheology of mortar, reduction in capillary absorption in air-cured condition acceleration of cement hydration,	Gupta and Kua (2019)
Wood sawdust	300 and 500 °C	Filler	1, 2, 5, 8%	Mechanical tests, density, porosity, drying shrinkage, water absorption, scanning electron microscope	Fog-cured	Improvement of early age compressive strength, 1% biochar shows comparable drying shrinkage	Dixit et al. (2019)
Wood sawdust	300 and 500 °C	Filler	2%	Internal RH, thermal gravimetric analysis, mechanical tests, water sorptivity, scanning electron microscope	Fog-cured, Air-cured	A higher degree of hydration by adding pre-soaked biochar, Improvement of strength and water tightness	Paiva et al. (2019)
Corn stover	550 °C, a heating rate of 15 °C min^−1^, residence time:25 m	Filler	2, **4**, **6**, 8%	Compressive test, X-ray diffraction, Fourier transform infrared spectroscopy, scanning electron microscope	Carbon dioxide curing	Increase in carbon dioxide uptake due to the addition of biochar	Praneeth et al. (2020)
Wood sawdust	500 °C heating rate of 10 °C min^−1^	Cement replacement	2, 5, 8%	thermal gravimetric analysis, isothermal calorimetry, compressive	Ambient curing	Increase in hydration degree and the heat of hydration due to biochar addition	Dixit et al. (2019)
Rice husk	500 °C, a heating rate of 1 °C s^−1^	Silica fume replacement	40%	Rheology, isothermal calorimetry, density, compressive test, capillary test	Water-cured at 26 °C	Plastic viscosity and yield stress of cenosphere mortar increased, acceleration of cement hydration, 15–20% higher compressive strength retention by mortar exposed to 450 °C	Gupta and Kua (2020)
Coconut shell	500 °C, a heating rate of 10 °C min^−1^	Cement replacement	5%	Rheology, isothermal calorimetry, thermal gravimetric analysis, density, compressive test, capillary test, shrinkage		Biochar with higher permeability and lower pore tortuosity resulted in a higher reduction in autogenous shrinkage	Gupta et al. (2020a)
Sorghum, manure, cotton stalk, algae	210–600 °C	Filler	2%	Compressive test, isothermal calorimetry, density, water absorption	Fog-cured	The high carbon content of biochar contributes to the strength development, reduction in capillary absorption, improvement of long-term strength by32%	Gupta et al. (2020b)

The biochar is used as a filler and a replacement for cement, sand and aggregates, silica fume, and others. The adopted tests are provided along with the curing conditions and the main outcomes. The bold percentage represents the optimum biochar dosage

#### Impact on properties of fresh mixtures

Gupta et al. (2018a) reported that adding 8% biochar to cement mortar can result in an 11% reduction in flow rate (workability). This study indicated that adding a high dosage of biochar (5–8%), which is produced at high temperatures, results in a firmer mortar mix. Furthermore, according to Cuthbertson et al. (2019) as the biochar content increases, the amount of water required to keep cementitious materials workable should increase. When 1.5 grammes of biochar are mixed with 10 grammes of cement in a concrete mix, the water-to-cement ratio should be increased from 0.4 to 0.48 to provide free water. The water holding capacity of the added biochar tends to retain higher amounts of free water in its porous structure, which explains the higher reduction in flowability of mortar (Gupta et al. 2018b). Due to the low density and porous structure of biochar particles, it has been shown that the density of fresh mortar decreases as the biochar content increases. On the other hand, the air content of fresh mortar increases as the amount of biochar added increases (Gupta et al. 2018b).

Several studies have been conducted on the effect of biochar on the rheological properties of cement mortar. Gupta et al. examined the early age characteristics of biochar-cement composites. The authors investigated the yield stress and plastic viscosity of mortar at various times after mixing water. In comparison with plain mortar and mortars containing coarser biochar particles (particle sizes of 2–10 μm), the incorporation of ground biochar (particle sizes of 0.1–2 μm) facilitated cement paste application into formwork due to its decreased viscosity (Gupta and Kua 2019). Biochar particle size had an effect on the yield stress of cement mixes, with finer biochar grains exhibiting a lower value (Gupta and Kua 2019). At a concentration of up to 3% by weight, biochar affects the workability of fresh mixtures, as indicated by a decrease in flowability and an increase in yield stress and plastic viscosity of the fresh paste. Gupta and Kashani attribute this effect to biochar's angular shape, fine particle size, and water-absorbing capabilities (Gupta and Kashani 2021).

#### Impact on transfer properties

Water absorption is critical for the durability and long-term functioning of cementitious materials. The effect of adding biochar to cement composites, either as a filler or as a cement substitute, on the sorptivity of cement composites has been extensively investigated in the literature. An increased void fraction in mortar results in an increase in capillary water absorption. Furthermore, increased capillary absorption results in the deterioration of the mortar/concrete structure (Akhtar and Sarmah 2018a) and/or the proliferation of germs in the indoor environment (Gupta et al. 2018a). Furthermore, resistance to external fluid penetration into the mortar mix is critical for enhancing the material's longevity, as porous networks facilitate easy entry of corrosive chemicals into the structure when hydraulic pressure is applied.

Gupta et al. investigated the effect of incorporating biochar produced at various temperatures on the capillary absorption and water penetration depth of mortar. The study was based on adding 1–8% (by weight) of biochar produced at 300 and 500 °C. The results indicate a reduction in water absorption, compared to the control, by approximately 33% by the addition of 1% of biochar produced at both temperatures, while a 2% dosage resulted in a reduction of 17 and 14% for the biochar produced at 300 and 500 °C, respectively. Higher doses resulted in increased water absorption rates for biochar produced at both temperatures. The findings indicate that a 1–2% dosage effectively reduces water absorption by mortar. This implies that using biochar-based mortar in construction can reduce water seepage via capillary absorption, thereby restricting mould and germ growth in the indoor environment (Gupta et al. 2018a). In terms of depth of water penetration, the results indicate a significant reduction compared to plain mortar under all scenarios, in terms of dosage and biochar production temperatures. However, the highest reduction is observed at a dosage of 1%, where a reduction of approximately 65 and 61% is achieved for biochar produced at 300 and 500 °C, respectively (Gupta et al. 2018a).

#### Impact on cement hydration

Hydration is required for the microstructural development of cementitious materials. Hydration products include non-crystalline hydrated phases such as calcium-silicate hydrate gel, hydrated crystalline phases such as calcium hydroxide, and non-hydrated crystalline phases with varying chemistry, microstructure, and size scale. Calcium-silicate hydrate deposits around cement grains and binds mortar components together to form a stiff structure, whereas calcium hydroxide forms in water-filled pores. Depending on the type and characteristics of the biochar, the creation of a dense structure, due to a filler effect, during the initial stage of development inside the cement product may hinder chemical enhancement, thereby prohibiting the formation of calcium-silicate hydrate and delaying pozzolanic activity (Akhtar and Sarmah 2018b).

Gupta and Kashani reported that at 3- and 7-day ages, adding 3% biochar (produced from waste peanut shell at 500 °C) to cement paste and cement-fly ash paste enhanced the degree of hydration by 15–23%. Increased hydration resulted in the formation of a solid network and a reduction in capillary porosity, both of which contributed to the development of compressive strength in biochar-cement mortar when compared to control (Gupta and Kashani 2021). Moreover, Gupta and Kua demonstrated that biochar could have a significant effect on the hydration of cement paste. The cumulative heat of hydration (a proxy for the degree of hydration in cement mixtures) was higher in cement pastes containing 1% mixed wood biochar than in plain paste. Coarser biochar generated more heat in this experiment than finely ground biochar (Gupta and Kua 2019).

Furthermore, Gupta et al. (2020a) reported increases in cumulative hydration heat of 10, 9.1, and 9% after 7 days of hydration, when 5% cement was replaced with processed coconut shell biochar, commercial biochar, and wood waste biochar, respectively. Additionally, regardless of the type of feedstock used, adding biochar to the cement paste accelerated hydration and increased the rate of hydration heat. In another study, replacing cement with 5% coarse biochar (250–500 μm) from mixed wood sawdust resulted in a 30% increase in heat of hydration after 96 h (Dixit et al. 2019). Gupta et al. (2021), in another study, reported that after 7 days, adding 1–2% biochar derived from rice husk and mixed wood generated a cumulative heat of hydration comparable to that of the control cement mortar. In another investigation, cumulative hydration heat was reduced by 15% in cement pastes incorporating 2% dairy manure biochar. This was attributed to the high level of ash (84%), which contains a substantial quantity of calcium, phosphate, and magnesium and hence retards hydration. Furthermore, the addition of sorghum biochar to cement paste was associated with a lower heat of hydration and a slower hydration reaction in the same study (Gupta et al. 2020b).

#### Impact on mechanical properties

The characteristics of biochar incorporated into cementitious materials influence their mechanical properties. When employed as a filler in cement mortar, independent of the water-cement ratio, biochar has a greater effect on the early strength of the mortar. Biochar particles are frequently smaller in size than both the average grain size of cement and the macropores found in most cement pastes. Fine particles have a critical role in filling macropores in mortar, resulting in increased compactness and stress transfer performance under load. Additionally, incorporating biochar into mixtures with a lower water-to-cement ratio results in a stiffer texture and affects the compactness of the mortar (Gupta et al. 2018a).

The optimal biochar content for increasing the hardened density of cement mortar was determined to be between 1–2% (Gupta et al. 2018a). Increased biochar dosages may result in the deterioration of the system performance. It was reported that mortar containing 5–8% biochar has a considerably reduced hardened density (Gupta et al. 2018a). The ductility of concrete containing more than 10% biochar was shown to be similarly influenced (Cuthbertson et al. 2019). Several studies have demonstrated that adding up to 2% biochar increases compressive strength. However, in some cases, there was a slight reduction in compressive strength at 7 and 28 days of age (Gupta et al. 2018a, b). Additionally, it has been established that using 2% wood sawdust biochar as a filler increases the splitting tensile strength of cement composites (Gupta and Kua 2018).

Cosentino et al. (2019) reported that specimens containing 0.8 and 1% biochar created from softwood biomass at 800 °C had greater flexural strength and fracture energy values than specimens made entirely with cement. Moreover, Suarez-Riera et al. (2020) studied the effect of biochar on the strength of cement paste during the mixing process. In the first set, biochar was immediately added to the water and superplasticizer mixture and then mixed with the dry ingredients, whereas, in the second set, all dry cement and biochar particles were combined prior to adding the water and superplasticizer. In the former state, adding 2% biochar enhanced flexural strength by 15%, whereas combining it with the second approach resulted in an 8% decrease in strength.

#### Impact on insulating properties

When biochar was utilised in place of sand and coarse particles in concrete, the noise reduction coefficient of the concrete containing 10 and 15% biochar by weight was 0.45, exceeding the threshold for materials with good sound absorption capabilities. Furthermore, in comparison with ordinary concrete, the application of biochar improved the thermal insulating properties of the concrete. Thermal conductivity was significantly decreased at 1 and 2% biochar by weight, with temperature-dependent conductivities ranging from 0.208 to 0.230 W mK^−1^ and 0.192–0.197 W mK^−1^, respectively. While this does not classify the material as a building insulating material, it does improve the thermal insulating properties of the concrete and, as a result, the energy efficiency of structures made of the material. According to the results of numerous concrete tests, it appears that including modest quantities of biochar into concrete may result in ideal material properties. The concrete's heat conductivity was improved between 1 and 2% by weight, and water usage was kept to a minimum in this range (Cuthbertson et al. 2019).

### Biochar in asphalt

The application of biochar in asphalt has been investigated within the literature. The addition of straw-derived biochar significantly improved the high-temperature performance of asphalt. Biochar performed comparably to commercial coal in this investigation, and it was determined that 6% of straw-derived biochar powder should be used in asphalt applications (Gan and Zhang 2021).

Additionally, biochar was investigated as an asphalt binder. Kumar et al. (2018) examined the feasibility of using biochar generated from mesua ferrea seed cover waste as a binder modifier/extender for asphalt. Five different biochar contents (0, 5, 10, 15, and 20% by weight of binder) and two distinct sources of basic asphalt binders were used to make bio-asphalt binders. The flow behaviour, permanent deformation, fatigue, and ageing properties of biochar-modified binders were evaluated and compared to those of control binders, without biochar.

The addition of biochar enhanced the viscosity of asphalt binders and exhibited Newtonian behaviour up to 20 wt.% biochar concentration used in the study. The use of biochar enhanced the permanent deformation resistance of the superpave rutting parameter (G*/sin) at high service temperatures. The addition of biochar reduced the sensitivity of bio-asphalt binders to ageing as measured by the rheological ageing index. Multiple stress creep and recovery experiments demonstrated that biochar reduced accumulated strain and non-recoverable compliance, thereby enhancing the rutting resistance of the binders. The stress sensitivity of all binders was determined to be within the specified limits, and it decreased as biochar concentration increased. The study's findings indicated that biochar generated using mesua ferrea seed cover waste could be a viable material for enhancing the performance of asphalt binders (Kumar et al. 2018).

Zhang et al. (2018d) investigated the use of biochar as a potential modifier for enhancing the rheological properties of asphalt binders. In this experiment, biochar with particle sizes ranging from 75 to 150 μm and less than 75 μm was used, with concentrations of 2, 4, and 8%. For comparison, flake graphite with a particle size distribution of less than 75 μm and a content of 4% was utilised as a modifier. The binder performance was evaluated using scanning electron microscopy, rotating viscosity testing, dynamic shear rheometer, and bending beam rheometer tests.

The results indicated that adding both biochar and graphite can improve the rotational viscosity of asphalt binders. The viscosities of the biochar-modified binders with smaller particles were greater than those with larger particles. All binders met the rotating viscosity criteria of less than or equal to 3000 mPas at 135 °C. Furthermore, the porous structure and rough surface of the biochar resulted in a greater adhesive interaction in the binder as compared to flake graphite. Consequently, the biochar-modified asphalt outperformed the graphite-modified asphalt in terms of high-temperature rutting resistance and anti-ageing qualities. Moreover, the asphalt binder modified with smaller particles and a larger biochar concentration showed obvious improvements. Finally, it was observed that binders modified with biochar particles of less than 75 μm and 4% concentration offer enhanced low-temperature crack resistance, as compared to other modified binders (Zhang et al. 2018d).

Zhou et al. (2020) evaluated the chemical structural properties and phase separation of biochar-modified bio-asphalt using molecular dynamics and numerical modelling. They reported that biochar improved the high-temperature performance of the bio-asphalt system and enhanced its oxidation resistance. Furthermore, biochar facilitated enhanced resistance to ageing. On the other hand, biochar did not affect the low-temperature hardening of the chemical composition in the bio-asphalt system.

### Biochar in biocomposite materials

Numerous biocomposite materials using biochar have been developed to perform a variety of purposes in construction, including insulation, moisture regulation, and protection from electromagnetic radiation. The unique physicochemical characteristics of biochar enable these materials to have enhanced functionality.

#### Insulation

Jeon et al. (2019) developed a latent heat storage biocomposite using coconut oil as phase change material and pinecone, pine sawdust, and paper mill sludge biochar. The biocomposite was developed using a novel vacuum impregnation method. Differential scanning calorimetry, scanning electron microscopy, thermogravimetric analysis, Fourier transform infrared spectroscopy, specific heat, and bulk density measurements were used to determine the thermophysical characteristics of the material.

The differential scanning calorimetry analysis revealed that pine sawdust biochar pyrolysed at 550 °C and subsequently impregnated with coconut oil had the highest latent heat storage capacity of 74.6 J g^−1^. The latent heat performance of pinecone biochar pyrolysed at 200 °C, pinecone biochar pyrolysed at 500 °C, and steam-activated pine sawdust biochar pyrolysed at 550 °C was also remarkable, achieving values of 61.9, 62.6, and 62.8 J g^−1^, respectively. The thermogravimetric analysis demonstrated that all developed biocomposites exhibited enhanced thermal stability, whereas the Fourier transform infrared spectroscopy examination revealed no chemical interaction between the biochar and coconut oil. The thermal conductivities of the biocomposites were found to range between 0.030 and 0.098 W mK^−1^. Four out of seven samples were found to have excellent heat-insulating properties, achieving a second or higher grade in the heat insulating material examination. The bulk density analysis yielded minimum values ranging from 0.43 to 0.91 g cm^−3^ (Jeon et al. 2019).

After evaluating all of the data, the biocomposite was determined to be suitable for use as a latent heat storage insulating material. The core properties of the biochar served as a phase change support material, allowing the biocomposite to store more phase change material per mass unit. When utilised as an insulation material, the low thermal conductivity of the developed biocomposite appeared to be sufficient to provide adequate insulation. Additionally, the latent heat storage biocomposite was made entirely using eco-friendly materials, which is more sustainable (Jeon et al. 2019).

#### Moisture regulation

Wood is a robust and sustainable material used in construction, facilitating good thermal insulation properties. However, structural faults may develop due to decay from moisture, reducing dimensional stability. Furthermore, when the moisture level is constantly high, this might lead to mould development, posing a health risk to occupiers. Jeon et al. investigated the development of a wood-based biocomposite incorporating biochar to compensate for the shortcomings of wood in terms of moisture stability. The wood-derived biocomposite samples were prepared by a hot-pressing process, and their hygrothermal performance was evaluated using experimental methods and simulations (Jeon et al. 2021).

The thermal conductivity of the biocomposite with a 10% biochar concentration was found to be 7.98% lower than that without the biochar. The high porosity and microstructure of biochar contributed to its low heat conductivity. The bending strength of the wood-derived biocomposite decreased with increasing biochar concentration, which was attributed to the pore structure of the biochar and the poor compatibility of the biochar surface with chemical functional groups and adhesives. However, the samples with the lowest bending strength of 7.67 Mpa could be considered strong, and the samples could be used as finishing materials. When biochar was applied, the water vapour resistance factor increased, indicating that the hydrophobic functional groups on the surface of the biochar had a greater effect on moisture permeability than the pore structure. According to the findings, the wood-derived biocomposite is appropriate for use as a building material due to its high insulating properties, ability to manage moisture, as well as resilience to climate change (owing to the carbon storage capacity of its raw materials). Given that biochar properties can be controlled, such as pore characteristics and surface functional groups, by varying the raw materials and pyrolysis conditions, it is necessary to investigate which characteristics have a greater impact on the moisture control of wood using different biochar types, in addition to the one used in this study (Jeon et al. 2021).

#### Electromagnetic radiation protection

Due to the rapid advancement of wireless communication and electronic technology in the modern period, electromagnetic radiation and electromagnetic interference have posed a serious threat to the proper operation of precision electronic devices and to human health. A simple preparation process was used by Yin et al. (2020b) to create a hybrid material using sorghum straw biochar with iron and nickel metals. Due to its high performance, this eco-friendly hybrid biochar can be employed as a new low-frequency microwave absorbent. Owing to the combined effect of interface polarisation, dipole polarisation, natural resonance, eddy current and conduction loss, multi-reflection and scattering, and better impedance matching, the as-prepared hybrid material exhibited outstanding low-frequency microwave absorption capabilities. At a preparation temperature of 600 °C, the highest reduction level attained was 44.18 decibels at 0.49 gigahertz, with an effective absorption bandwidth of 0.17–0.99 gigahertz for 2 mm and a board effective absorption bandwidth of 0.53–2.49 gigahertz for 1 mm thickness. Meanwhile, the highest reduction level at a preparation temperature of 700 °C was 46.36 decibels at 0.81 gigahertz with an effective absorption bandwidth of 0.49–1.43 gigahertz and a board effective absorption bandwidth of 0.89–2.81 gigahertz for the same thicknesses, respectively. This study offered a novel way for developing an eco-friendly material for high-efficiency, low-frequency microwave absorption using sorghum straw biochar.

Furthermore, a fluorine-doped biochar-based carbon material was synthesised and employed by Sutton et al. as an additive in producing several materials for electromagnetic radiation protection. Overall, by developing biochar with high electron density and stability, the material could absorb and buffer ionising radiation in excess of and/or equal to industry standards. The carbon–fluorine bond strength, as well as fluorine intercalation within the pores, demonstrated that the developed biochar is an appropriate option for radiation protection. As a result, biochar was able to replace several radiation-protective materials while also providing environmental benefits (Sutton et al. 2021).

Moreover, bamboo-derived biochar that had been pyrolysed at 1100 °C was used by Li et al (2018c) as a conductive filler in an ultra-high-molecular-weight polyethylene/linear low-density polyethylene composite. Biochar with a dense nano-porous structure, a large specific surface area, and high electrical conductivity was produced through high-temperature pyrolysis. The addition of that biochar significantly improved the electrical conductivity and electromagnetic interference shielding efficacy of the composites. The composites with 80% biochar had a conductivity of 107.6 S m^−1^ and a very high electromagnetic interference shielding efficiency of 48.7 decibels at 1500 megahertz, both of which are among the highest values ever reported for conductive polymer composites fabricated by melt processing and exceed the electrical conductivity and electromagnetic interference shielding requirements of many applications.

### Biochar in smart bricks

Because bricks are a common building material and a critical component of the construction process, it is required to replace conventional bricks with a more sustainable option. The potential for incorporating biochar into bricks has been recognised due to the thermal insulating qualities of biochar when used in place of sand and aggregates in concrete, as well as its direct impact on weight reduction and carbon dioxide removal from the atmosphere, which have been highlighted in previous research. As a result, the production of sustainable bricks, also referred to as smart bricks, which include biochar, can reduce permanent loads on buildings and consequently reduce the amount of steel reinforcement and concrete used in facilities, in addition to its thermal insulation, which results in energy savings through reduced use of air conditioners and heaters. Above all, its potential for carbon sequestration significantly contributes to climate change.

### Summary

This section demonstrated the potential utilisation of biochar within construction. To conclude, our analysis showed that biochar could be utilised as a cementitious additive, conferring various structural and functional advantages. Additionally, the inclusion of biochar into asphalt and the manufacture of sustainable bricks exhibited several advantages. Furthermore, the literature has demonstrated the potential for biochar to be included in biocomposites for improved insulation, electromagnetic radiation protection, and moisture control, all of which have demonstrated positive results.

Biochar's long-term storage capacity in civil infrastructure makes it a viable carbon reservoir. However, it is critical to keep in mind that the usable life of civil structures varies significantly. When such structures reach the end of their useful lives, they can be recycled further, or the material can be disposed of sustainably in landfills for long-term storage. Civil infrastructure, in general, is a robust carbon reservoir, and additional research is needed to optimise and enhance the potential use of biochar for value maximisation in the building sector.

## Energy storage

The long-term storage of pyrogenic carbon within advanced bio-based materials has been suggested by Schmidt et al. (2019a) as a valid strategy, as long as the material is not subjected to thermal degradation or oxidisation throughout its lifetime, once recycled or at termination. The utilisation of biochar in energy storage applications can present a very interesting route to creating considerable value while serving the ultimate purpose of prolonged carbon sequestration. However, synthesising biochar-based materials appropriate for energy storage applications involves advanced knowledge and engineering. The deployment of various biochar functionalisation approaches necessitates a careful examination of each technique to ensure that the desired material features, as well as carbon stability, are achieved while minimising the environmental impact of the process in order to enhance the carbon removal potential of this approach. This section will further explore the literature on synthesising advanced biochar-based material for supercapacitor and battery applications.

### Biochar-based materials for supercapacitors

**Fig. 9:**
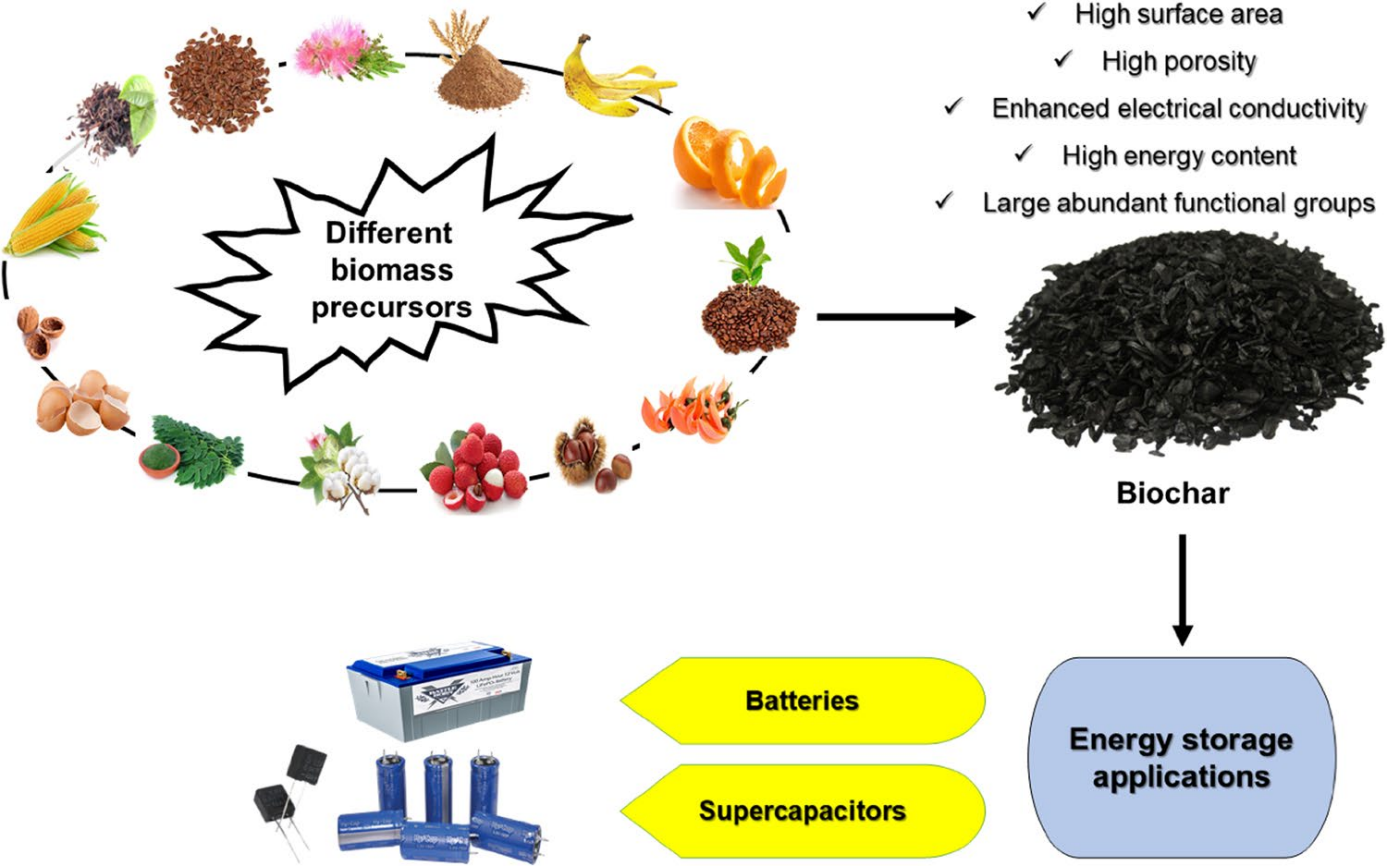
Biochar derived from biomass is illustrated schematically for use in energy storage applications. Biochar is first produced from a variety of biomass feedstocks, including fruit peel, eggshells, seeds, dedicated crops, and biomass waste precursors. Subsequently, various modification treatments are carried out to synthesise a biochar-based material with a high surface area, high porosity, improved electrical conductivity, high energy content, and a large number of abundant functional groups. This biochar-based material has the potential to be used in energy storage applications such as batteries and supercapacitors due to its low cost, efficiency, and active energy storage properties

Globally, there has been a rise in research into sustainable and renewable energy sources during the last few years. The advantages of supercapacitors, among other energy storage technologies, have been thoroughly investigated in the literature, including their rapid charge–discharge capabilities, high power density, and cycle stability. Compared to other carbon compounds, biochar materials have attracted considerable interest due to their low cost, efficiency, and active energy storage properties (Fig. 9).

Li et al. synthesised biochar with a hierarchical porous structure from the flowers of *Cotinus coggygria* as a supercapacitor using a new composite activator. The produced biochar had a surface area of 959.04 cm^2^ g^−1^. After 36,000 cycles, the biochar demonstrated a reversible capacitance of 279.9 F g^−1^ (6 M potassium hydroxide) through a current density of 5 A g^−1^. In the same context, the biochar demonstrated an exceptional specific capacitance of 413.5 F g^−1^ in 1 M sulphuric acid. The composite activator’s synergism contributed to its enhanced specific capacitance and stable cycling performance. Several advantages of adopting hierarchical porous biochar include ease of preparation, low cost, and superior performance (Li et al. 2020f).

Biochar, produced by pyrolysing biomass at low temperatures, has a low conductivity and is therefore rarely employed in energy storage. Zhang et al. (2019b) developed a compact biochar material based on sodium lignosulfonate, graphene oxide, and p-phenylenediamine through evaporation and pyrolysis at low temperatures, which was enriched with dinitrogen and exhibited high conductance. Sodium lignosulfonate was used as the carbon source for biochar, while the reduced graphene oxide provided excellent conductivity. P-phenylenediamine was then mixed with graphene oxide and sodium lignosulfonate to produce compact char via polymerisation through the process of evaporation and pyrolysis.

Nitrogen content (14.3%), density (1.98 g cm^−3^), yield (69.5 weight %), and conductivity (0.078 S. m^−1^) of the prepared biochar were all high. The biochar single-electrode had an excellent volumetric and gravimetric specific capacitance of 950 F cm^−3^ and 480 F g^−1^ at 1 A g^−1^ in 1 M sulphuric acid, respectively, with excellent cycle stability. Furthermore, the constructed symmetric supercapacitor exhibited a remarkable energy density in 1 M sulphuric acid, both volumetrically (26.5 Wh L^−1^) and gravimetrically (13.4 Wh k^−1^). With a 1 M sodium sulphate electrolyte, significantly improved performance was observed (21.6 Wh kg^−1^ and 42.8 Wh L^−1^). This novel concept demonstrates a feasible method for producing materials combined with biochar for use as supercapacitor electrodes in sustainable energy systems (Zhang et al. 2019b).

Jiang et al. employed pyrolysis and subsequent potassium hydroxide/potassium nitrate activation to produce porous carbon-based materials derived from cotton, keeping the fundamental hollow-tubular fibre structure. The carbon compound developed had a suitable specific capacitance of ~ 2.8 × 10^2^ and ~ 2.1 × 10^2^ F g^−1^ at 1 and 100 A g^−1^, respectively. A long cycle life is exhibited, which may be ascribed to its active porous structure and unique hollow tubular form. (Jiang et al. 2019).

Biochar is well recognised as a multifunctional material capable of generating high-performance carbon compounds for energy and environmental applications. Gao et al. (2020) used the inner shell of *Torreya grandis*, a typical lignocellulosic biomass waste, to fabricate hierarchically porous carbon composites via a planned pyrolysis/activation approach. This work presents an effective and economically viable process for carbonising/activating waste biomass in order to create hierarchical porous biochar for sustainable energy storage and environmental applications. The synthesised biochar exhibited exceptional energy storage performance in 1 M sodium sulphate, with high capacitance and stability (3% loss after 5000 cycles).

Cai et al. (2017) employed the stems of *Moringa oleifera* to synthesise carbon nanosheets in a hierarchically porous form via a one-step pyrolysis procedure. The synthesised material exhibited a unique porous nanosheet structure, a large pore volume of 23 × 10^–1^ cm^3^ g^−1^, a high surface area of approximately 2.3 × 10^3^ m^2^ g^−1^, sufficient porosity, and doped heteroatoms (N and O). It had a 283 F g^−1^ specific capacitance at 0.5 A g^−1^ and a 28% capacitance loss at 50 A g^−1^. Additionally, the three-electrode arrangement displayed superior long-term cycle stability.

Sun et al. (2018) created nitrogen-doped biochar materials with high concentrations of mesopores obtained from waste corn silks. It had a pore volume and a surface area of approximately 2.0 cm^3^ g^−1^ and 1764.8 m^2^ g^−1^, respectively. The examined electrode maintained an exceptional capacitance of 3.5 × 10^2^ F g^−1^ during 5 × 10^3^ cycles, reflecting a loss of only 0.8% of its original value. Despite this, the biochar electrode acquired a high energy density of 17.8 Wh kg^−1^ and an outstanding capacitance of 2.6 × 10^2^ F g^−1^ by using an upgraded electrolyte composed of alizarin red solution sulphuric acid and bromoamine acid.

Tea leaves were employed as a carbon source in pyrolysis with potassium hydroxide activators to produce functionalised carbon. At 1200 °C, the functionalised carbon's pore volume and surface area were 0.512 cm^3^ g^−1^ and 911.92 m^2^ g^−1^, respectively. Asymmetric supercapacitors made of this material exhibited a specific capacitance of 167 F g^−1^ at a current density of 1 A g^−1^ (Song et al. 2019a).

Wu et al. (2019b) proposed employing potassium hydroxide activation to synthesise hierarchical microrods of albizia flower-derived biochar. After activation with electrolyte (potassium hydroxide) at 1173 K, the self-nitrogen-doped microrod biochar-based material demonstrated a distinctive porous structure. The material showed a total pore capacity of 1.47 cm^3^ g^−1^ and an exceptional surface area of approximately 2.8 × 10^3^ m^2^ g^−1^. As a result of these improvements, a 390 F g^−1^ specific capacitance and exceptional cycle stability were achieved, with a 3% loss after 5000 cycles at 1 A g^−1^. Similarly, the 1 M sodium sulphate electrolyte had a power density of 42 Wk g^−1^ and a specific energy density of 26.3 Whk g^−1^.

According to Lu et al. (2020), a biochar material derived from lotus leaves has been developed with exceptional electrochemical properties. They first pyrolysed the lotus leaves at 700 °C in a tube furnace to obtain biochar. Furthermore, the resulting biochar was activated at 700 °C using potassium hydroxide (1:3, wt). The pore volume of the biochar-based material produced was 1.37 cm^3^ g^−1^, and the specific surface area was 2350.8 m^2^ g^−1^. An exceptional capacitance of 478 F g^−1^ was attained when employed as a supercapacitor electrode. Additionally, after 5 × 10^3^ cycles in a two-electrode arrangement, the created supercapacitor lost around 10.9% of its initial capacitance.

Zhou et al. (2018b) used bristlegrass seeds to make a honeycomb-like biochar material. Due to its hierarchical pore interconnected three-dimensional honeycomb-like structure, multi-heteroatom doping, and large surface area, the built electrode exhibited a high capacitance of 3.9 × 10^2^ F g^−1^. Additionally, it possessed a high-rate capability, a long-life cycle, and a loss of around 2.8% of its initial value over 1 × 10^4^ cycles. Additionally, the supercapacitor electrode displayed a 20.15 Wh k g^−1^ energy density at a 500 Wk g^−1^ power density in sodium sulphate (1 M).

Su et al. (2018) synthesised an advanced biochar-based material from mango stone biowaste using a two-step chemical etching process. The material's energy density and specific capacitances were 27.6 Whk g^−1^ and 358.8 F g^−1^, respectively, at 0.5 A g^−1^.

Li et al. (2020g) developed a biochar material from flaxseed waste. Pyrolysis of the precursor was carried out in an inert environment at temperatures ranging from 600 to 800 °C. Furthermore, the material was mixed (1:4) with potassium hydroxide and activated at 700 °C in an argon atmosphere. The resulting material had a large surface area of 3.2 × 10^3^ m^2^ g^−1^. Capacitance of 398 and 369 F g^−1^ were obtained in sulphuric acid and potassium hydroxide electrolytes, respectively, and retained 98.1% of their capacitance after 10,000 cycles (Li et al. 2020g).

In another study, Zhao et al. (2019) used a potassium hydroxide activation approach to directly pyrolyse litchi shells in order to create porous carbon nanosheets/particle composites. Additionally, by balancing the potassium hydroxide activating agent to the litchi shells at a ratio of 3:1, a large surface area of around 1.1 × 10^3^ m^2^ g^−1^ was obtained, and the material exhibited an extraordinary capacitance of 2.20 × 10^2^ F g^−1^.

In a one-step pyrolysis process, another study used chestnut shells and melamine as an activating agent to produce porous carbon materials doped with heteroatoms. Porous carbon derived from chestnut shells had a specific surface area of 691 m^2^ g^−1^ and contained several heteroatoms such as nitrogen, oxygen, and sulphur. Carbon electrodes, as prepared, had a 402.8 F g^−1^ specific capacitance at 0.5 A g^−1^ in potassium hydroxide electrolyte (Wan et al. 2019a).

Potassium carbonate and potassium bicarbonate are increasingly being employed in research to develop materials for electrode applications. Mu et al. synthesised hierarchical porous carbons from bean curd and potassium carbonate. After a single activation stage, the resulting specific surface area was 2514 m^2^ g^−1^. At 0.1 A g^−1^, this material displayed a specific capacitance of 486 F g^−1^ in 1 M sulphuric acid and ~ 400 F g^−1^ in 6 M potassium hydroxide in a three-electrode configuration. Additionally, a ~ 12 Wh kg^−1^ energy density was reported when a sulphuric acid solution (1 M) was used as the electrolyte, as well as a continuous capacitance efficiency of up to 1 × 10^4^ cycles (Mu et al. 2019).

Additionally, Karnan et al. synthesised porous carbon from corncob using a one-step potassium hydroxide activation method, providing a specific surface area of 800 m^2^ g^−1^. Due to the porous and disordered structure of the derived material, it displayed a high capacitance of approximately 3.9 × 10^2^ F g^−1^, a high energy density of around 25 Wh kg^−1^, and a high power density of approximately 175 kW kg^−1^ (Karnan et al. 2017).

Yuan et al. (2019) synthesised hybrid carbon materials from chitosan by using potassium bicarbonate. The gelatin-modified chitosan-derived biochar exhibited a distinctive tangerine pith-like structure with a specific surface area of 927.2 m^2^ g^−1^. The as-prepared biochar electrode material showed a high specific capacitance of 330 F g^−1^ and a loss of around 10% of its initial capacitance after 1 × 10^4^ cycles at 10 A g^−1^ in a 6 M potassium hydroxide electrolyte. With a power density of 900 Wk g^−1^ and 34 Wh kg^−1^ energy density, chitosan-derived biochar material outperformed most commercial devices.

Vijayakumar et al. (2019) reported using cotton-based biochar materials as supercapacitor electrodes. Waste cotton was pyrolysed at approximately 873 K in an inert atmosphere and then activated at 1123 K in a nitrogen environment using potassium hydroxide at a mass ratio of 1:3. At a voltage window of ~ 3.2 V, the synthetic biochar had a specific surface area of 1.9 × 10^3^ m^2^ g^−1^ and volumetric capacitance of 0.87 × 102 F cm^−3^ at a current density of 1 A g^−1^. Additionally, the synthetic electrode exhibited a higher volumetric energy density of 31.0 Wh L^−1^, resulting in a greater active mass loading and a superior voltage window. Furthermore, the cotton-based biochar materials demonstrated exceptional durability after 1.5 × 10^4^ cycles at a current density of 4 A g^−1^.

Zhu et al. (2019) proposed nitrogen-doped biochar materials via pyrolysis and subsequent activation utilising egg white. The honeycomb structure of the biochar material generated from egg white resulted in a surface area of 2.9 × 10^3^ m^2^ g^−1^. The structure of connected micro-and mesopores enhanced electrolyte ion diffusion channels and increased the area of contact between the electrolyte and the electrode. Additionally, a three-electrode configuration exhibited a capacitance of approximately 3.4 × 10^2^ F g^−1^ and an 8.3% capacitance loss after 10,000 cycles.

Zhang et al. (2019c) synthesised biochar material from wheat bran that had been activated with sodium hydroxide. When the mass rate of the used precursor and sodium hydroxide was 1:4, the produced biochar-based material with hierarchical micropores and mesopores had a high surface area of 2.6 × 10^3^ m^2^ g^−1^. Additionally, it possessed an extraordinary capacitance of 2.9 × 10^2^ F g^−1^ and a capacitance loss of 6% over 5 × 10^4^ cycles when used as a supercapacitor electrode.

Khan et al. (2020c) produced a biochar-based material with a hierarchically porous structure from a naturally withering rose petal using potassium hydroxide/potassium nitrate activation. The resulting carbon material has a large surface area of approximately 2 × 103 m^2^ g^−1^, excellent conductivity, and a hierarchical pore structure. It performed exceptionally, with a capacitance of 0.35 × 103 F g^−1^ at a current density of 1 A g^−1^, a rate ability of 0.17 × 103 F g^−1^ at a current density of 150 A g^−1^, and a 4.6% loss of original capacitance after 140,000 cycles at 100 A g^−1^.

Zheng et al. (2017) employed water hyacinth to synthesise porous hierarchical carbon as a biomass precursor through pyrolysis and subsequent activation. It showed a large surface area of 2.3 × 103 m^2^ g^−1^ and a hierarchical structure. When utilised as a supercapacitor electrode, it exhibited an astounding capacitance of 3.4 × 102 F g^−1^, leading to enhanced energy and power densities.

Several investigations have demonstrated that adding heteroatoms to the surface of carbon materials increases the capacitance of supercapacitors. For instance, adding nitrogen, phosphorus, and oxygen atoms to the surface may enhance the interaction of ions with the electrode leads, hence increasing the wettability of the electrolyte solution. Due to the presence of functional groups of oxygen and nitrogen atoms on the surface, the porous carbonaceous material exhibits acidic or alkaline properties, which promote electrode–electrolyte interactions. Wan et al. (2019a, 2019b) demonstrated the production of melamine-activated biochar containing nitrogen, oxygen, and sulphur heteroatoms in a hierarchical structure from chestnut shells. The biochar materials were created using an adequate graphitisation degree and a heteroatom content of 3.79% nitrogen, 13.35% oxygen, and 0.52% sulphur. In a three-electrode arrangement, the synthesised biochar had a large surface area and a high specific capacitance of around 6.9 × 10^2^ m^2^ g^−1^ and 4 × 10^3^ F g^−1^, respectively. It also demonstrated good stability, with a 3.4% of capacitance over several 20,000 cycles.

Wang et al. (2020e) developed biochar materials produced from durian shells and doped with phosphorus, oxygen, and nitrogen atoms. The biochar material exhibited a limited surface area, a pore structure dominated by micropores, and abundant heteroatoms. The resulting composite performed effectively as an electrode in a supercapacitor, with a capacity of 1.8 × 10^2^ F g^−1^ at a current density of 0.5 A g^−1^. It demonstrated cycle stability, with only a 12% loss after 10,000 cycles. Furthermore, in sulphuric acid and potassium iodide aqueous electrolyte solutions, the studied biochar had a capacitance of 5.6 × 10^2^ F g^−1^.

Liu et al. developed nitrogen, oxygen, and sulphur co-doped biochar material using kraft lignin as a precursor for use in supercapacitors that did not require an activation process. The preparation method was simple, cost-effective, and environmentally friendly. In addition to their hierarchically porous structures, the kraft lignin-based biochar exhibited a notable surface area of 1.3 × 10^3^ m^2^ g^−1^. The biochar electrode produced showed exceptional specific capacitance of approximately 2.4 × 10^2^ F g^−1^ at 0.2 A g^−1^, good conductivity, 18.2% capacitance loss of its original capacitance at 40.0 A g^−1^, and 91.6% cycling stability over 10,000 cycles. In an aqueous electrolyte, the examined supercapacitor had an energy density of 0.67 × 10^2^ Wh kg^−1^ and a remarkable power density of 1.80 kW kg^−1^. It was also kept at 0.32 × 10^2^ Wh kg^−1^ at an ultrahigh power density of 0.4 × 10^2^ kW kg^−1^ (Liu et al. 2019c; Liu et al. 2019d).

Fu-Qiang et al. (2018) proposed a one-step approach for synthesising nitrogen-doped cotton as an environmentally friendly carbon source. The 750 °C biochar had a large surface area of 0.48 × 10^3^ m^2^ g^−1^ and a significant nitrogen concentration of 6.80%. Despite having a lower specific surface area than earlier materials, this material achieved a maximum capacitance of around 2.5 × 10^2^ F g^−1^ in a 1 M sulphuric acid electrolyte and lost approximately 6% of its original capacitance after 1 × 10^4^ cycles at a current density of 15 A g^−1^.

Another approach is to dope polymeric compounds on porous carbon surfaces. Polyaniline is a polymer molecule that can be used to increase pseudo-capacitance. These materials have attracted much interest due to their availability, ease of processing, as well as chemical and environmental stability.

Lyu et al. (2018b) examined composites made from yeast-derived nitrogen-doped carbon and polyaniline. The porous nature of biomass-based carbon materials and their large surface area provide active sites for polyaniline, which may help alleviate the pseudo-capacitance loss. The examined composite had a remarkable specific capacitance of 0.5 × 10^3^ F g^−1^ at a current density of 1 A g^−1^ and a high-rate capability. Meanwhile, the composite symmetric supercapacitor device demonstrated an excellent capacitance of 0.1 × 10^3^ F g^−1^ at a current density of 1 A g^−1^ and 4.6% loss of original capacitance after 0.5 × 10^4^ cycles. Supercapacitor electrodes based on biochar derived from biomass and its composites are presented in Tables 19 and 20, respectively.

**Table 19:** Supercapacitor electrodes produced using synthesised biochar derived from various biomass materials

Type of activation	Type of biomass	Electrolyte	Conditions of measurements (A g^−1^)	Specific capacitance (F g^−1^)	Surface area (m^2^ g^−1^)	Reference
Pyrolysis	Tea leaves	Potassium hydroxide	1	167	911.92	Song et al. (2019a)
Potassium hydroxide/Pyrolysis	Coffee ground	1-Ethyl-3-methylimidazolium bis(trifluoromethylsulfonyl)imide	0.5	404	–	Choi et al. (2018)
Potassium hydroxide	Hyacinth	Sulphuric acid	0.5	344.9	2276	Zheng et al. (2017)
Potassium hydroxide	Garlic skin	Potassium hydroxide	0.5	427	2818	Zhang et al. (2018e)
Potassium hydroxide	Litchi shell	Potassium hydroxide	0.1	222	1122.6	Zhao et al. (2019)
Zinc chloride/ potassium hydroxide	Mango stone	–	0.5	358.8	1497.8	Su et al. (2018)
Pyrolysis	Moringa leaves	Potassium hydroxide	50	234	1327	Peng et al. (2018b)
Pyrolysis/ Melamine	Chestnut shell	Potassium hydroxide	0.5	402.8	961	Wan et al. (2019a)
Potassium carbonate solution/ hydrothermal	Walnut shell	Potassium hydroxide/PVA	0.5	255	62	Xu et al. (2017)
Potassium hydroxide	Crab shell and rice husk	6 M potassium hydroxide	0.5	474	3557	Peng et al. (2019)
Potassium hydroxide	Sisal	6 M potassium hydroxide	0.5	415	2889	Liu et al. (2019d)
Potassium hydroxide/potassium nitrate	Rose flower	6 M potassium hydroxide	1	350	1980	Khan et al. (2020c)
Potassium hydroxide	Poplar anthers	6 M potassium hydroxide	0.5	361.5	3639	Song et al. (2018)
Potassium hydroxide	Shaddock endothelium	1 M 1-Butyl-3-methylimidazolium tetrafluoroborate (BMIMBF_4_/AN)	0.2	550	1265	Yang et al. (2019d)
Potassium hydroxide	Rice husk	6 M potassium hydroxide	0.5	315	3263	Luo et al. (2019)
Potassium hydroxide	Castor shell	6 M potassium hydroxide	1	365	1527	Okonkwo et al. (2020)
Hydrated potassium oxalate	Cornstalk	1 M sodium sulphate	0.5	461	2054	Li et al. (2020h)
Sodium hydroxide	Cellulose	6 M potassium hydroxide	0.5	288	1588	Song et al. (2019b)
Potassium hydroxide	Cottonseed husk	6 M potassium hydroxide	0.5	1694.1	1694.1	Chen et al. (2018c)
Potassium hydroxide	Cotton stalk	1 M sodium sulphate	0.2	254	1964.46	Tian et al. (2017)

The type of activation method along with the biomass feedstock are reported. Furthermore, the electrolyte used and measurement conditions, along with the obtained specific capacitance and surface area, are provided

**Table 20:** Composite supercapacitor electrodes based on biochar derived from biomass

Composite materials	Biomass material	Current density (A g^−1^)	Number of cycles	Capacitance (F g^−1^)	Stability (%)	Electrolyte used	References
Hierarchically porous carbon/nitrogen	Houttuynia	1	10,000	473.5	95.74	6 M potassium hydroxide	Shang et al. (2020)
Nanofibers/nickel–cobalt oxides	Typha domingensis	1	5000	142	92.1	6 M potassium hydroxide	Golmohammadi and Amiri (2020)
Porous carbon/phosphorus	Elaeocarpus tectorius	0.2	1000	385	96	1 M sulphuric acid	Nirosha et al. (2020)
Porous carbon/ iron (III) oxide	Hemp straw	1	5000	256	77.71	6 M potassium hydroxide	Jiang et al. (2020)
Carbon nanosheet/ nickel (II) hydroxide	Peach gum	1	5000	350	83.9	6 M potassium hydroxide	Yu et al. (2019b)
Activated mesoporous carbon/nitrogen/sulphur	Datura metel seed pod	1	3000	340	95.24	1 M sulphuric acid	Raj et al. (2020)
Hierarchically porous carbon/N/N	Carboxymethyl cellulose ammonium	1	10,000	465	86.3	3 M potassium hydroxide	Meng et al. (2020)
Activated carbon/ copper (II) chloride	Lotus pollen	1	10,000	496	90.8	1 M sodium sulphate	Wan et al. (2021)
Hierarchically porous carbon/nitrogen/sulphur	Rape pollen	1	20,000	361	94.5	6 M potassium hydroxide	Wang et al. (2019d)
Porous carbon/nitrogen	Ginkgo leaves	0.5	12,000	323.2	99	6 M potassium hydroxide	Wang et al. (2019e)
Porous Carbon three-dimensional honeycomb structure	Cotton Seed Husk	0.5	5000	238	91	6 M potassium hydroxide	Chen et al. (2018c)
Hierarchical porous carbon/copper(I) oxide/copper(II) oxide	Bamboo leaves	1	10,000	147	93	3 M potassium hydroxide	Wang et al. (2019e)
Bio- carbon xerogel/graphene	Bamboo	1	10,000	189	100	6 M potassium hydroxide	Yang et al. (2018)
Biochar/nitrogen	Peanut shells	0.2	10,000	447	91.4	1 M sulphuric acid	Gandla et al. (2021)
Porous carbon hollow-sphere/nitrogen/sulphur	Puffball spores	0.5	5000	285	80.3	2 M potassium hydroxide	Shang et al. (2021)
Three-dimensional porous carbon/ manganese dioxide	Banana peel	10	3000	170	98	1 M sodium sulphate	Yang and Park (2018)

The composite materials and underlying biomass feedstocks utilised are reported. Furthermore, the operating conditions, such as the current density (A g^−1^), capacitance (F g^−1^) and the percentage stability, are presented

### Biochar-based materials for batteries

Lithium-ion batteries are the most widely used in energy storage due to their high working voltage, exceptional energy density, and compact size. Significant attempts have been made to develop low-cost, environmentally friendly carbonaceous materials with improved charge storage capacity. Biochar derived from biomass has recently attracted considerable interest as anodes for lithium-ion batteries due to their large surface area, porous nature, and potential for lithium-ion storage.

Zhang et al. (2018f) synthesised nitrogen-doped carbon with a porous structure using soybeans as a carbon and nitrogen source. The resulting carbon honeycomb-like structure featured a connected porous network with a significant surface area of 0.109 × 10^4^ m^2^ g^−1^ and a range of pore sizes (meso- and macropores). After multiple cycles, the carbon produced from soybeans exhibited reversible capacities of 0.275–0.31 × 10^3^ mAh g^−1^.

Murali et al. (2019) utilised a microwave-based conversion technique for synthesising a hierarchically porous structured carbon material derived from peanut shells. A material with a high specific surface area of 0.2099 × 10^4^ m^2^ g^−1^ was produced. At a current rate of 0.1 C, the examined anode exhibited an exceptional specific capacity of 0.142 × 10^4^ mAh g^−1^, which was attributed to the large surface area and the presence of unstructured monolayer graphene forms in the synthesised material.

Domestic food waste, specifically cooked rice, was employed to produce microporous and heteroatom-doped carbon using pyrolysis and subsequent activation. Cooked rice-derived biochar was produced at various temperatures, and the effect on electrochemical performance was investigated by using it as a lithium-ion battery material. The produced anodes had a remarkable specific capacitance of 0.1 × 10^3^ mAh g^−1^ at 100 mA g^−1^ after 100 cycles (Packiyalakshmi et al. 2019).

Prasanna et al. developed a novel method for enclosing milled silicon particles in nitrogen-doped carbon to minimise major volume changes in silicon during lithium intercalation and to increase its electrical conductivity. As a carbon source, chitosan was employed, which is the only naturally known alkaline polysaccharide biomaterial containing nitrogen. The researchers utilised a conventional hydrothermal process followed by pyrolysis. The silicon and nitrogen modified electrode outperformed milled silicon in terms of electrochemical performance, with a discharge capacity of 0.940 × 10^3^ mAh g^−1^ and a columbic efficiency of 97% over 50 cycles. After 50 cycles, the nitrogen modified electrode outperformed conventional graphite electrodes in discharge capacity and columbic efficiency, with a discharge capacity of 0.49 × 10^3^ mAh g^−1^ and a columbic efficiency of 99.8% (Prasanna et al. 2019).

Several studies have recommended biomass-derived carbon as an electrode material for lithium-ion batteries due to its simple ion movement, high conductivity, and ability to buffer volume changes throughout the electrochemical process. Meanwhile, a better understanding of biochars' physical characteristics and electrochemical activity is necessary to enhance their performance in lithium-ion battery applications (Qi et al. 2017).

Zhang et al. developed a high-performance carbon synthesis method using pinecones as a promising biomass source. Pinecone-derived hard carbon exhibited a specific capacity of 0.334 × 10^3^ mAh g^−1^ at a current density of 30 mA g^−1^ over 120 cycles (Zhang et al. 2017a).

Additionally, Damodar et al. demonstrated that hard carbon could be formed by pyrolysing palmyra fruit palms at temperatures ranging from 500 to 900 °C. In terms of electrochemical performance, hard carbon treated at 700 °C outperformed soft carbon, exhibiting a reversible capacity of 0.3970 × 10^3^ mAh g^−1^ at a current density of 30 mA g^−1^. Due to their excellent microstructural characteristics and high interlayer spacing, carbon layers performed better electrochemically in battery applications (Damodar et al. 2019). Wang et al. (2019f) created hard carbons using caltrop shells in a simple pyrolysis technique. With a reversible capacity of 285.2 mAh g^−1^ at a current rate of 0.1 C, the studied carbon produced at 1300 °C exhibited a significant sodium-storage capacity.

Romero-Cano et al. (2019) demonstrated how to extract hard carbons from grapefruit peels and how to use them in sodium-ion batteries. After pyrolysis at low temperatures, a decrease in sodium reversibility was detected, which was addressed using the functionalisation process. At a current density of 0.180 × 10^3^ mAh g^−1^, grapefruit hard carbons had a reversible capacity of 0.180 × 10^3^ mAh g^−1^ at a current rate of 0.1 C. It was hypothesised that the functionalisation resulted in less reactive surface groups, allowing sodium to diffuse more easily.

Yang et al. (2020) developed a functionalised hard carbon synthesis technique using walnut shells. The carbons studied had a reversible capacity of 0.245 × 10^3^ mAh g^−1^ at 100 mA g^−1^. The increased electrochemical performance was attributed to the regulation of pore size, namely, mesopores with diameters ranging from 2 to 20 nm, which enabled a more favourable charge storage behaviour on the larger carbon surface.

Gaddam et al. (2017) established a low-temperature pyrolysis procedure for producing hard carbon from spinifex nanocellulose. At a current density of 20 mA g^−1^, the studied carbon exhibited a reversible capacity of 0.39 × 103 mAh g^−1^. The improved electrochemical performance is attributed to the significant distance between the interlayers of spinifex-derived carbon, measuring 0.39 nm.

Wang et al. (2017f) used dandelions as precursors in a simple pyrolysis process to create hard carbon. The carbons developed had a reversible capacity of 0.36 × 10^3^ mAh g^−1^ at 50 mA g^−1^. Additionally, when the pyrolysis temperature increased, the average number of graphitic layers, their breadth and thickness, as well as their effect on electrochemical properties, increased.

Fu et al. (2018b) utilised apple pomace to synthesise a networked carbon material that can be used as an anode in battery applications. When used as an anode material, the three-dimensional networked carbon with shell-like structure and macropores with a notable diameter of 0.5 µm demonstrated a reversible capacity of 0.208 × 10^3^ mAh g^−1^ over 2.0 × 10^2^ cycles.

Wu et al. (2019c) synthesised hard carbons via lotus seedpod pyrolysis and then evaluated their performance as sodium-ion battery anodes. Hard carbon from lotus seedpods outperformed the control sample at 1200 °C, with a reversible capacity of 0.329 × 10^3^ mAh g^−1^ at a current density of 50 mA g^−1^ and an acceptable rate capability of 0.78 × 10^2^ mAh g^−1^ at a current density of 1 A g^−1^.

Zhang et al. (2018g) synthesised hard carbon in a honeycomb structure using pine pollen. The hard carbon exhibited a capacitance of 0.37 × 10^3^ mAh g^−1^ at a current density of 0.1 A g^−1^ and a proper and reversible capacitance of 0.203 × 10^3^ mAh g^−1^ after 200 cycles, which might be attributed to their 0.41 nm interlayer spacing, which reduces the resistance caused by the sodium ions.

Xiang et al. (2017a) synthesised a hard carbon material using orange peels via pyrolysis and subsequent potassium hydroxide activation. After 100 cycles, the resulting carbon demonstrated acceptable electrochemical properties as anodes for battery applications, with reversible capacities of ~0.3 × 10^3^ and ~0.16 mAh g^−1^ at 0.5 and 1 A g^−1^, respectively.

Zhu et al. (2017) identified sorghum stalks as a biomass precursor to produce ecologically friendly and cost-effective hard carbon. After 50 cycles, the obtained samples demonstrated a superior reversible capacity of 0.245 × 10^3^ mAh g^−1^ at a current density of 20 mA g^−1^ and a rate capability of 0.172 × 10^3^ mAh g^−1^ at a current density of 200 mA g^−1^.

*Typha*, a perennial freshwater plant popularly referred to as cattail, was used by Shen et al. (2019) to aid in the synthesis of hard carbon. *Typha* hard carbon is produced in a simple method, utilising phosphoric acid activation and a low pyrolysis temperature of 500 °C. Numerous sodium ion sites are provided by the *typha*-derived hard carbon, which comprises small pores with nitrogen and oxygen functional groups. It exhibited a reversible capacity of 0.205 × 10^3^ mAh g^−1^ after 400 cycles at a current density of 100 mA g^−1^.

Wang et al. (2018e) established a simple acid pretreatment followed by temperature-controlled pyrolysis to produce rice husk-derived hard carbon. The study examined the effect of pyrolysis temperature on the electrochemical properties of synthesised rice husk-derived hard carbon. The samples exhibited a significant reversible capacity of 0.372 × 10^3^ mAh g^−1^ at a current density of 25 mA g^−1^.

Additionally, Li et al. (2019e) reported the high-temperature pyrolysis and subsequent potassium hydroxide activation processes that were used to produce oatmeal-derived hard carbon. Interlayers with a thickness of 0.39 nm were observed in the resulting material, as were graphitic micro-crystallites with a low specific surface area and mesoporous properties. At current density of 20 mA g^−1^, the produced carbon had a significant initial reversible capacity of 0.272 × 10^3^ mAh g^−1^.

Benítez et al. (2018) produced microporous carbon using almond shells as a biomass by-product via pyrolysis and a subsequent activation treatment. This carbon had a significant surface area of 0.97 × 10^3^ m^2^ g^−1^ and a specific capacitance of 0.91 × 10^3^ mAh g^−1^ at a current density of 100 mA g^−1^.

Hencz et al. (2017) investigated the electrochemical performance of porous hierarchical carbon produced from seaweed biomass for use as a cathode material. The produced samples had a surface area of 0.151 × 10^4^ m^2^ g^−1^ and a porous volume of 1.48 cm^3^ g^−1^, which were available in microporous, mesoporous, and macroporous forms. They determined a reversible capacity of 826.4 mAh g^−1^ after 70 cycles as cathodes at a 0.2 C rate.

Mangosteen peels were also used to create hierarchically porous carbon with an exceptional surface area of 0.324 × 10^4^ m^2^ g^−1^ and a pore volume of 1.58 cm^3^ g^−1^; at current rates of 0.5 and 2 C, the produced carbon exhibited outstanding specific capacities of 0.87 and 0.569 × 10^3^ mAh g^−1^, respectively (Xue et al. 2017).

Yuan et al. (2017b) developed mesoporous lamellar carbon from bagasse using a single-step high-temperature pyrolysis approach. With a surface area of 0.545 × 10^3^ m^2^ g^−1^, increased reversibility of ~ 0.49 × 10^3^ mAh g^−1^ at a current rate of 1 C and significant rate capability.

Additionally, Xiang et al. (2017b) used ferric chloride and zinc chloride as activators to synthesise a porous carbon doped with nitrogen and capable of forming nanosheets with an outstanding pore volume of 1.85 cm^3^ g^−1^ and a large specific surface area of 0.154 × 10^4^ m^2^ g^−1^. The prepared cathodes functioned effectively, reaching an initial specific capacitance of 0.876 × 10^3^ mAh g^−1^ at a current rate of 0.5 C and was maintained at 0.594 × 10^3^ mAh g^−1^ after 100 cycles. The increased electrochemical characteristics may be attributed to the large pore volume, hierarchically porous structure, and large surface area, all of which allow for more efficient ion and electron transport channels.

Carbon derived from biomass could be used as an interlayer in lithium-sulphur batteries to reduce polysulfide shuttling. For example, virgin cellulose paper was used to produce a carbon material with fewer functional groups. When the material was used as an interlayer, discharge capacities of 0.83 and 0.71 × 10^3^ mAh g^−1^ were reached at current rates of 0.2 and 1 C, respectively. Electrochemical studies indicated that pyrolysed paper successfully collected and utilised dissolved polysulphides to enhance the performance of lithium-sulphur batteries (Li et al. 2017b).

Furthermore, soybean shell was used as a dual source to synthesise carbon doped with a high concentration of nitrogen (1.7%). After functionalisation, the resultant carbon had a notable surface area of 0.844 × 10^3^ m^2^ g^−1^, and when employed as an air cathode in a zinc-air battery, it exhibited a high current density of 0.111 × 10^3^ mA cm^2^ and a high power density of 150 mW cm^2^ (Zhao et al. 2018b).

Zhang et al. (2017b) used banana peel to synthesise microporous carbon doped with nitrogen, providing an exceptional surface area of 0.1097 × 10^4^ m^2^ g^−1^. When used in zinc-air batteries, the produced carbon surpassed the platinum loaded on carbon material in terms of discharge performance and power density. Table 21 shows the electrodes for batteries based on biochar derived from biomass.

**Table 21:** Electrodes for batteries based on biochar derived from biomass

Treatment type	Type of biomass	Conditions and measurements	Specific capacity (mAh g^−1^)	Surface area (m^2^ g^−1^)	Reference
Pyrolysis, activation	Marine	0.1 mA g^−1^	640	535–1488	Guo et al. (2018b)
Potassium hydroxide/pyrolysis	Pinecone	30 mA g^−1^	334	239	Zhang et al. (2017a)
Pyrolysis	Soybean	2/4 C	310/275	1089.8	Zhang et al. (2018f)
Potassium hydroxide	Cooked rice	100 mA g^−1^	1000	1899	Packiyalakshmi et al. (2019)
Microwave	Peanut shell	0.05 C	680	525	Murali et al. (2019)
Pyrolysis and phosphoric acid activation	Almond shell	100 mA g^−1^	915	967	Benítez et al. (2018)
Pyrolysis	Seaweed	0.2 C	826.4	1510.71	Hencz et al. (2017)
Curing and annealing	Prawn shell	100 mA g^−1^	1735	–	Lian et al. (2018)
Pyrolysis	Bagasse	1 C	494	545	Yuan et al. (2017b)
Pyrolysis/ potassium hydroxide activation	Mangosteen	0.5/2 C	870/569.2	3244	Xue et al. (2017)
Potassium hydroxide/pre-pyrolysis	Crab shell	0.1 C	971.3	1298.2	Shao et al. (2017)
Pyrolysis	Caltrop shell	0.1 C	285.2	9.58	Wang et al. (2019f)
Pyrolysis	Cellulose	0.2 C	830	–	Li et al. (2017b)
Phosphoric acid/pyrolysis	Typha	100 mA g^−1^	204.8	559.73	Shen et al. (2019)
Pyrolysis	Rice husk	25 mA g^−1^	372	0.27	Wang et al. (2018e)
Pyrolysis	Grapefruit peel	0.1 C	180	10.9	Romero-Cano et al. (2019)
Pyrolysis	Cherry petal	20 mA g^−1^	300.2	1.86	Zhu et al. (2018)
Pyrolysis	Argan shell	25 mA g^−1^	300	380	Dahbi et al. (2017)
Pyrolysis	Spinifex	20 mA g^−1^	386	154	Gaddam et al. (2017)
Pyrolysis	Dandelion	50 mA g^−1^	361	9.9	Wang et al. (2017f)
Pyrolysis	Lotus seedpod	50 mA g^−1^	328.8	751–108	Wu et al. (2019c)
Pyrolysis	Pine pollen	0.1 A g^−1^	370	171.54	Zhang et al. (2018g)
Potassium hydroxide/pyrolysis	Orange peel	0.5/1 A g^−1^	301/156	638	Xiang et al. (2017a)
Pyrolysis	Sorghum stalk	20/200 mA g^−1^	245/172	234.55	Zhu et al. (2017)

Different types of biomass feedstocks were used and the operating conditions along with the measurements are reported. The obtained specific capacities in mAh g^−1^ and the surface area in m^2^ g^−1^ are presented

### Summary

This section extensively reviewed the literature on synthesising advanced biochar-based materials for energy storage applications. In conclusion, the manufacture of such material requires a sophisticated functionalisation procedure to provide the desired characteristics. Biochar was used as a precursor for subsequent functionalisation in almost all the studies presented. Biochar production as a first stage was commonly carried out via pyrolysis. This was usually followed by an activation or modification step carried out in the presence of various reagents.

The main challenge observed along this path is the high temperatures employed throughout the process to attain the desired physical and chemical properties. In most of the investigations, the synthesised material exhibited an exceptional performance; however, the production process required operating temperatures of 700 °C and above, sometimes reaching 1200–1300 °C. Operating at such high temperatures is not advantageous from a carbon yield standpoint; however, temperatures around 700 °C facilitate excellent carbon stability. Therefore, it is important to assess the stable carbon yield achieved by the process. Additionally, the employment of various chemical reagents during the functionalisation stage adds an environmental burden that must be carefully evaluated. Life cycle analyses must be conducted to have a better understanding of the carbon sequestering potential of advanced biochar-based materials used in energy storage applications.

Another aspect to consider is the fate of such material upon completing its useful life. As previously discussed, thermal regeneration is not applicable from a carbon removal perspective. Therefore, the ultimate end-of-life destination is the disposal of such materials in landfills for long-term storage.

Despite the high energy requirements, chemicals utilised, and low carbon yield that might be associated with the synthesis of advanced biochar-based products, an apparent perceived benefit is the direct replacement of fossil-based carbon materials. In moving forward, it is recommended that research should focus on developing production processes that employ lower temperatures with a maximum of 700–800 °C and use low-cost and environmentally friendly functionalisation techniques, in order to maximise the carbon removal potential of this application as well as provide an economically viable product that can be applied at a large scale. Finally, research should also focus on investigating biomass sources that are abundantly available for large scale deployment.

## Conclusion

Given the current state of climate emergency, developing technically and commercially viable carbon removal systems is critical. Biochar-based carbon removal systems have recently received much attention, which has prompted a greater understanding of the complete value chain. The final application of biochar is crucial to its validation as a carbon sink and should be carried out as sustainably as possible while adhering to regulatory and technical standards in order for biochar technology to serve its carbon removal purpose. As previously stated, biochar can be used in various applications as long as it is not used for energy production. Furthermore, the biochar must not be subjected to thermal degradation or oxidisation during or after its service life. In this context, we thoroughly evaluated the key biochar-based carbon sink applications, including agronomy, animal farming, anaerobic digestion, composting, environmental remediation, building materials, and energy storage. As demonstrated, exceptional value can be extracted in each of the applications discussed, in addition to the carbon removal potential achieved.

Despite the numerous advantages and benefits associated with the production and application of biochar, it appears that large-scale biochar deployment faces certain challenges. Generally, the major challenges to the process are technical, economic, and socioeconomic in nature. In addition, government bodies, particularly in developing countries, lack knowledge about the benefits and applications of biochar, resulting in a lack of supporting legislation and policies, as well as a lack of public awareness about the financial benefits of investing in biochar-related projects. Social acceptance of the biochar production process is also critical. Awareness of the available opportunities, including job creation, associated with an investment in biochar production is vital. In developed countries, pyrolysis plants are well-established. However, poorly built plants in underdeveloped nations (e.g. stoves and drum kilns) may negatively impact greenhouse gas emissions and the release of highly toxic compounds. Furthermore, additional costs and energy are required for feedstock pre-treatment/drying, which adds to the existing constraints.

In addition to the challenges outlined above, biochar production in developing countries has additional challenges, such as advancing technical competency and knowledge at both the individual and institutional levels. Furthermore, there is a need to address some misconceptions concerning biomass utilisation as a feedstock. Fears about the use of biochar, as well as the consequences on the competition for labour, land, and food crops, are another impediment in Sub-Saharan Africa (Gwenzi et al. 2015). Such challenges could be addressed by raising public awareness of biochar and its benefits at the community and governmental levels.

Moreover, global standardisation of biochar production is required in terms of gaseous emissions, stability, and large-scale applications. As the biochar industry advances, there is a rising demand for universal standards to govern biochar production and ensure its quality. There are now two recognised standards: the European biochar certificate (also referred to as EBC) and the international biochar initiative (referred to as IBI). While the primary objective of developing these standards is to improve the quality control and assurance of the emerging biochar industry, and while several nations have aligned their biochar industries to these standards, they remain a voluntary option for producers. As a result, new legislation is currently required. However, the emerging carbon removal economy is already integrating such standards within market participation requirements. Furthermore, these voluntary standards will highly influence market regulations and dynamics moving forward.

As demonstrated, the use of pristine biochar in various applications is viable; however, to significantly improve the performance of the biochar, further functionalisation and engineering techniques may be necessary for certain applications. This usually requires further physical and chemical treatments, which need to be carefully examined for each process. The impact of such treatments on carbon stability as well as the environment should be thoroughly assessed. Furthermore, it is also important to consult the certification bodies in order to ensure that any modification methods employed are in line with certification requirements. Finally, detailed life cycle assessments (cradle to grave) of biochar systems must be performed for a wide range of feedstocks and production processes in order to determine the true potential for carbon removal depending on the carbon sink application for which the biochar is optimised.
